# Fish Biodiversity of the Vitória-Trindade Seamount Chain, Southwestern Atlantic: An Updated Database

**DOI:** 10.1371/journal.pone.0118180

**Published:** 2015-03-04

**Authors:** Hudson T. Pinheiro, Eric Mazzei, Rodrigo L. Moura, Gilberto M. Amado-Filho, Alfredo Carvalho-Filho, Adriana C. Braga, Paulo A. S. Costa, Beatrice P. Ferreira, Carlos Eduardo L. Ferreira, Sergio R. Floeter, Ronaldo B. Francini-Filho, João Luiz Gasparini, Raphael M. Macieira, Agnaldo S. Martins, George Olavo, Caio R. Pimentel, Luiz A. Rocha, Ivan Sazima, Thiony Simon, João Batista Teixeira, Lucas B. Xavier, Jean-Christophe Joyeux

**Affiliations:** 1 Departamento de Oceanografia e Ecologia, Universidade Federal do Espírito Santo, Vitória, ES, Brazil; 2 Programa de Pós-Graduação em Ecologia e Conservação da Biodiversidade, Universidade Estadual de Santa Cruz, Ilhéus, BA, Brazil; 3 Instituto de Biologia and SAGE/COPPE, Universidade Federal do Rio de Janeiro, RJ, Brazil; 4 Instituto de Pesquisas Jardim Botânico do Rio de Janeiro, Rio de Janeiro, RJ, Brazil; 5 Fish Bizz Ltda., São Paulo, Brazil; 6 Departamento de Ecologia e Recursos Marinhos, Universidade Federal do Estado do Rio de Janeiro, Rio de Janeiro, RJ, Brazil; 7 Departamento de Oceanografia, Universidade Federal do Pernambuco, Recife, PE, Brazil; 8 Departamento de Biologia Marinha, Universidade Federal Fluminense, Niterói, RJ, Brazil; 9 Departamento de Ecologia e Zoologia, Universidade Federal de Santa Catarina, Florianópolis, SC, Brazil; 10 Centro de Ciências Aplicadas e Educação, Universidade Federal da Paraíba, Rio Tinto, PB, Brazil; 11 Laboratório de Biologia Pesqueira, Universidade Estadual de Feira de Santana, BA, Brazil; 12 California Academy of Sciences, San Francisco, California, United States of America; 13 Museu de Zoologia, Universidade Estadual de Campinas, Campinas, SP, Brazil; ufrj, BRAZIL

## Abstract

Despite a strong increase in research on seamounts and oceanic islands ecology and biogeography, many basic aspects of their biodiversity are still unknown. In the southwestern Atlantic, the Vitória-Trindade Seamount Chain (VTC) extends ca. 1,200 km offshore the Brazilian continental shelf, from the Vitória seamount to the oceanic islands of Trindade and Martin Vaz. For a long time, most of the biological information available regarded its islands. Our study presents and analyzes an extensive database on the VTC fish biodiversity, built on data compiled from literature and recent scientific expeditions that assessed both shallow to mesophotic environments. A total of 273 species were recorded, 211 of which occur on seamounts and 173 at the islands. New records for seamounts or islands include 191 reef fish species and 64 depth range extensions. The structure of fish assemblages was similar between islands and seamounts, not differing in species geographic distribution, trophic composition, or spawning strategies. Main differences were related to endemism, higher at the islands, and to the number of endangered species, higher at the seamounts. Since unregulated fishing activities are common in the region, and mining activities are expected to drastically increase in the near future (carbonates on seamount summits and metals on slopes), this unique biodiversity needs urgent attention and management.

## Introduction

Despite the general perception that seamounts are small isolated spots scattered in remote areas, this habitat is one of the most extensive of all oceanic environments [1]. There are hundreds of thousands of seamounts [2] comprising an estimated area of approximately 28.8 million km² [1]. The largest contiguous area of seamounts is found in the central portion of the Pacific Plate, where most studies have been conducted [3]. The number of ichthyological surveys on seamounts has increased, and recent data from fishing [4–6] and SCUBA sampling [7–9] have been incorporated into an extensive database for seamount fishes [10–12]. This database has provided the opportunity to study several aspects of seamount fish biodiversity and ecology [10,13], as well as connectivity, biogeography and speciation [11,14–16]. However, biological surveys of seamounts remain sparse [1], mainly due heavy logistics and costs, and consequently extensive marine areas still remain poorly known [17].

**Fig 1:**
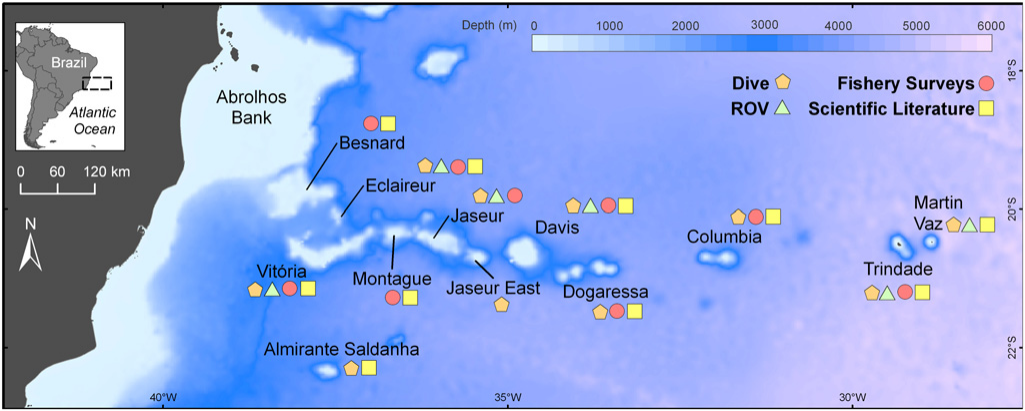
Vitória-Trindade Chain, Southwestern Atlantic. Sites surveyed in this study are named. Bathymetric data from Smith and Sandwell [105].

Data on south Atlantic seamounts is best described as patchy and of variable quality [18]. For a long time, most of the biological information available on the Vitória-Trindade Seamount Chain (VTC) (19°- 21°S, 28°- 38°W, Fig. 1) solely referred to the islands. Ichthyological surveys at Trindade Island date back to the early 1900’s [19–21]. Present knowledge depicts a rich reef fish fauna [22–26] connected to the continental coast through a stepping-stone process across the VTC seamounts [22,23,27]. However, the high number of endemic species at the islands indicates that genetic connectivity between the continent and islands is limited, although it could have been more effective during low sea levels [25,26,28]. Only two ichthyological surveys had been previously conducted on the VTC seamounts: a Brazilian-French expedition in 1987 [29,30], with use of bottom trawling and dredging, and the 1990’s Program of Evaluation of the Sustainable Potential of Living Resources in the Brazilian Exclusive Economic Zone (REVIZEE), with use of midwater trawls [31,32], surface and bottom longlines [33,34]. Despite constrained by the limited sampling methods, results from these studies allowed an initial biogeographical analysis in which the VTC was indicated as a Brazilian zoogeographical transitional zone [34].

The VTC is composed of volcanic mounts disposed in an E-W alignment, from 200 to 1,200 km off the Brazilian coast. Trindade and Martin Vaz Archipelago, the farthest locations from the continental shelf, are the only islands of the chain, and, therefore, the sole areas able to support species restricted to very shallow habitats. The remainder of the VTC is composed of 17 seamounts with height up to 2,500 m above the sea bed [35], where at least ten seamounts have summits with depths varying from 50 to 120 m below water surface. The VTC lays over the South American Plate, between 19° and 21°S, along a fracture zone disposed transversely to the Mid-Atlantic Ridge. The chain was formed by the activity of the Trindade hotspot mantle plume [35–37], with the plate moving westward at a rate of 23.1 km My^-1^ [36], but the development of its central segment may have been synchronous, involving an event associated with the lateral spreading of the plume over weaker mantle zones [38]. Despite this controversy, it is widely accepted that the VTC emerged during the Cenozoic, starting in the Tertiary (60–40 Mya) [39]. The oldest mounts are those nearer to the Brazilian continental shelf [40], while the islands emerged more recently, between 3 and 0.5 Mya [36]. Columbia is the seamount closest to the islands (250 km west of Trindade, Fig. 1) and is also the youngest seamount, with nearly 10 My [37].

Oceanic circulation in the western part of the VTC is dominated by the Brazil Current, which flows south from about 13° to 38° S [41]. This superficial current mostly follows the continental shelf edge and may form a barrier to larval movements and faunal migration from the adjacent coastline [42]. On the other hand, eddies, Taylor cones, dynamical uplifts and amplification of tidal movements are common oceanographic features associated with seamounts [43–45] and can contribute to water mass and biological connectivity. Upwelling events driven by topographical complexity and oceanographic features are also frequent and promote nutrient enrichment of the oligotrophic oceanic surface waters of the VTC region [45–47].

**Fig 2:**
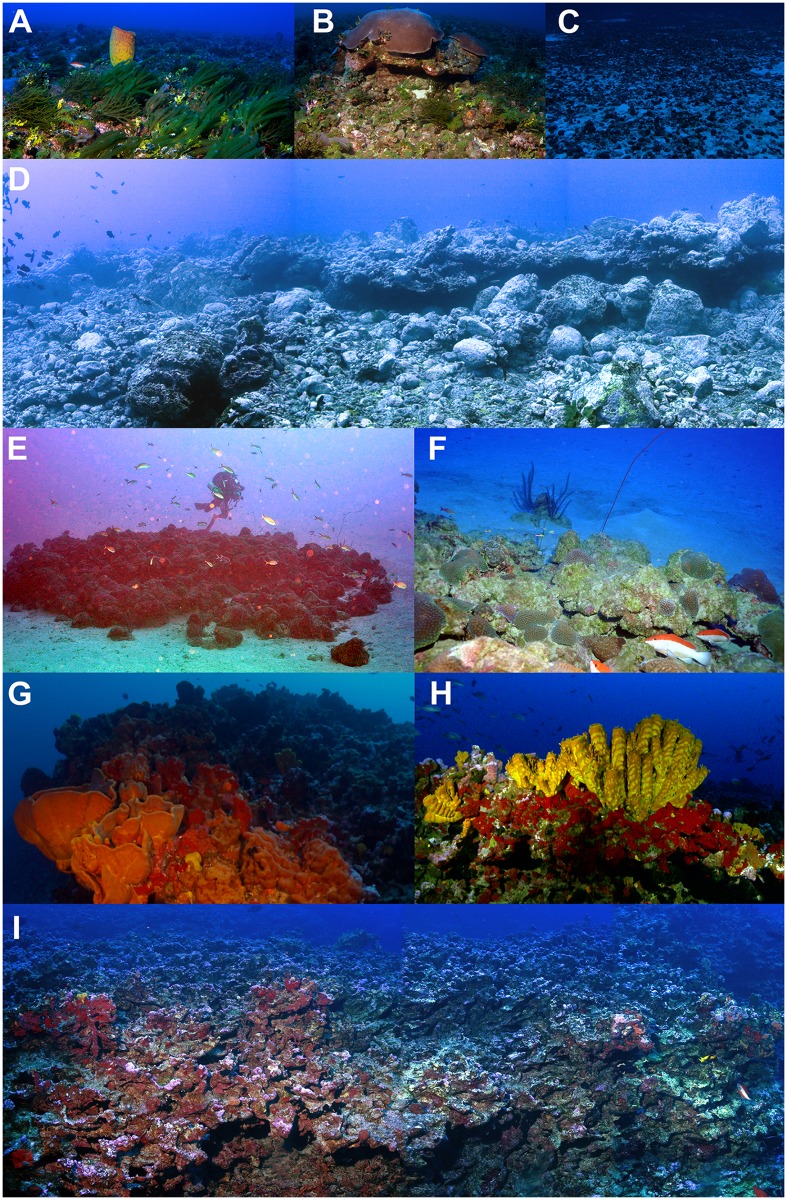
Diversity of habitats on the VTC. (A) rhodolith beds, extensively found on seamount summits and island’ shelves, (B) rocky reefs from Trindade and Martin Vaz islands, (C) patch reefs from Trindade Island, (D) Coralline reef structures covered of sponges at Davis Seamount; (E) High relief and complex reef structures that reaches depths of 17 m at Davis Seamount. Photos by R.M. Macieira, R. Francini-Filho, R.L. Moura, H.T. Pinheiro, PANGEA expedition.

Rhodolith beds are the main benthic habitat found at mesophotic depths (30–120m) of the VTC, with the calcareous algae nodules associated with many invertebrate species and frequently covered by macroalgae (Fig. 2; [48,49]). Calcareous algae that compose the rhodoliths are major benthic primary producers delivering substantial amounts of dissolved carbon in the oligotrophic waters of the VTC region [49]. Coralline and rocky reefs are common in the shallow zones of the islands (Fig. 2), but sparse and patchy biogenic reef structures are also found at mesophotic depths on seamount summits, with some high-relief structures reaching depths as shallow as 17 m and sheltering rich shallow water reef fish communities [50]. These biogenic reefs are predominantly built and covered with encrusting coralline algae, besides important contributions from sponges and corals (Fig. 2). Thirteen hermatypic coral species are known to occur in the VTC mesophotic zone [51].

Although sheltering a high diversity of habitats and species, the fragility of seamount ecosystems is widely recognized [33,52,53]. Worldwide, they have been targeted by intense fishing activities [54,55], leading to over-exploitation and habitat damage [11,34,56]. The VTC is targeted by Brazilian and foreign fishing vessels using surface and bottom longlines, hand lines and trawling [57]. Trawling by foreign vessels has been allowed in the continental slope and at seamounts off the N-NE Brazilian Economic Exclusive Zone (EEZ) [58]. On these habitats, overexploitation is generally followed by drastic reduction (boom-and-burst cycle, [52]) or even extinctions [59]. This occurs because seamounts and oceanic islands have similar features, such as low carrying capacity due to isolation and limited population size. Processes such as larval input from continental shelves or other oceanic sources are generally unable to sustain high fishing levels in these relatively small and isolated systems.

In order to better understand the biodiversity and distribution of species in the VTC, this paper presents and analyzes an extensive database about the composition of the fish assemblages associated to the VTC seamounts and islands (Fig. 1), highlighting the main biogeographical and macroecological implications from this new and updated database [17]. This assessment provides a comprehensive coverage of the VTC ichthyofauna built on the use of remotely operated underwater vehicles and mixed-gas technical diving with standard open circuit and rebreather apparatus, as well as from a compilation of unpublished information from scientific fishing, museum vouchers and literature records. This database includes new records and depth range extensions, and provides insights upon the structure of assemblages. The present paper also calls attention to the conservation of these unique ecosystems, and comments on human impacts that are already reaching these seamounts.

## Results

### Scientific Diving Contribution

The scientific diving expeditions yielded 128 fish species on the seamounts, 119 of them (93%) being new records, and 113 species at the two islands (12 new records) (see S1 Annotated Checklist). Known depth range was extended for 49 species, six to shallower and 43 to deeper waters (see S1 Annotated Checklist). Two new species belonging to the genera *Opistognathus* and *Lythrypnus* were found at seamounts and Trindade Island. Five species previously considered endemic to Trindade and Martin Vaz islands were recorded on seamounts [*Elacatinus pridisi*, *Halichoeres rubrovirens*, *Hypleurochilus brasil*, *Lythrypnus* sp.2 (as in [60]) and *Sparisoma rocha*]. However, the islands still shelter endemic fishes that were not found on the seamounts (*Acyrtus* sp., *Entomacrodus* sp., *Malacoctenus brunoi*, *Scartella poiti*, and *Tomicodon* sp.).

### REVIZEE and Fishery Surveys Contribution

The REVIZEE Program and our fishery surveys yielded 102 species over the VTC seamounts and 46 in the islands. These captures added 72 new records for the seamounts and 11 new records for the islands (see S1 Annotated Checklist). Known depth range was extended for 15 species, nine to shallower and six to deeper waters (see S1 Annotated Checklist).

### VTC Fish Diversity

A total of 273 fish species (26 elasmobranchs and 247 bony fishes) were recorded on the VTC (see S1 Annotated Checklist). The fish fauna of the VTC is composed of 21 orders and 82 families, with dominance of Perciformes (39 families, 145 species), followed by Anguilliformes (6 families, 23 species) and Tetraodontiformes (6 families, 22 species). Labridae was the most speciose family (22 species), followed by Epinephelidae (17), Carangidae (16), Myctophidae (14), Muraenidae (12), Carcharhinidae (11), Scorpaenidae (9), Gobiidae (8) and Pomacentridae (8). The most speciose genus was *Carcharhinus*, with 9 species, followed by *Diaphus* (8), *Gymnothorax* (7), *Sparisoma* (6), *Halichoeres* and *Scorpaena* (5), and *Chromis*, *Mycteroperca* and *Thunnus* (4). One hundred and eighty-nine species are primarily associated with reef environments, whereas 87 species have pelagic or bathydemersal habits. Most species have a wide geographic distribution; 58% are western or amphi-Atlantic and 22% are circumglobal. Twenty-two species occur only in the Brazilian Province (*sensu* [61]) (8% of the total or 14% of the reef fish fauna) and 11 species are endemic to the VTC: *Acyrtus* sp., *Elacatinus pridisi*, *Entomacrodus* sp., *Halichoeres rubrovirens*, *Hypleurochilus brasil*, *Lythrypnus* sp. 1, *Lythrypnus* sp. 2, *Malacoctenus brunoi*, *Scartella poiti*, *Sparisoma rocha* and *Tomicodon* sp.

Macro-carnivores composed the richest trophic guild (117 species), followed by macro-invertivores (58), planktivores (47) and roving herbivores (14). Most of the species are pelagic spawners (192) and the remainder lay demersal eggs (27) or are viviparous (28). Twenty-four species are considered endangered: 20 of them are listed in the IUCN Red List as critically endangered (CR; n = 2), endangered (ED; n = 2) or vulnerable (VU; n = 16). Eight species are listed as endangered in the Brazilian Red List [62]. Additional 13 species are considered near threatened (IUCN Red List) and nine are over-exploited (Brazilian Red List; see S1 Annotated Checklist). Habitats with the highest number of species were reefs, with 160 species, followed by rhodolith beds (130 species), water column (100) and sandy bottoms (28). The water column had the highest number of exclusive species (70 species only occur in this habitat), followed by reefs (59), rhodolith beds (20) and sand (7).

### Comparison between seamounts and islands

Two hundred and eleven fish species (67 families) were recorded on the seamounts and 171 (63 families) at the islands. One hundred and ten species (40%) were widely distributed across the VTC on both seamounts and islands, whereas 101 (37%) occurred exclusively on seamounts and 61 were exclusive to the islands (23%). Only six species were recorded at all sampled sites: *Balistes vetula*, *Cephalopholis fulva*, *Coryphopterus thryx*, *Holocentrus adscencionis*, *Malacanthus plumieri* and *Stegastes pictus*. Trindade Island features the richest fauna, followed by Vitória and Davis seamounts (Table 1).

**Table 1:** Number of species recorded in each sampling site of the Vitória-Trindade Chain, southwestern Atlantic.

	Vitória	Al Saldanha	Besnard	Montague	Jaseur	Jaseur East	Eclaireur	Davis	Dogaressa	Columbia	Trindade	Martin Vaz
Total number	108	28	44	45	48	71	38	103	70	43	173	73
Reef fishes	94	22	24	19	47	65	35	88	68	42	137	67
ACANTHURIDAE	3	0	0	0	3	3	2	3	1	0	2	2
BALISTIDAE	3	3	3	1	4	3	2	3	4	3	4	3
CHAETODONTIDAE	4	1	0	0	1	2	0	3	3	0	3	2
EPINEPHELIDAE	6	2	4	1	7	7	5	7	7	7	11	6
HAEMULIDAE	0	0	0	0	0	0	0	0	0	0	1	0
LABRIDAE	11	6	0	0	6	5	4	14	7	3	16	10
LUTJANIDAE	2	0	3	0	3	1	2	4	1	2	3	0
POMACANTHIDAE	3	1	0	1	2	3	3	3	2	1	2	2
POMACENTRIDAE	5	1	0	0	2	6	3	6	2	2	7	6
SERRANIDAE	4	0	0	0	3	1	1	1	2	0	2	1

**Fig 3:**
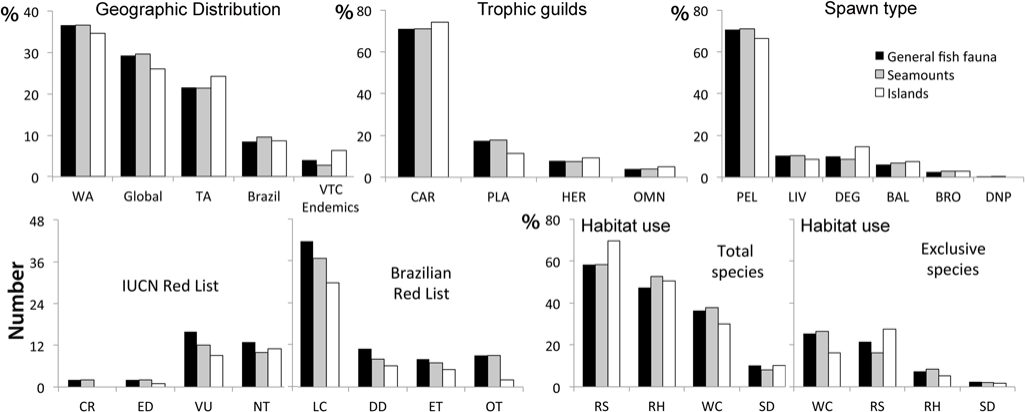
Summary of the fish assemblage characteristics found along the VTC. Geographic distribution (WA = Western Atlantic; TA = Trans Atlantic). Trophic guilds (CAR = carnivores; PLA = planktivores; HER = herbivores; OMN = omnivores). Spawn type (PEL = Pelagic eggs; LIV = Live birth; DEG = Demersal egg; BAL = Balistid-type demersal eggs; BRO = Brooded egg; DNP = Demersal eggs no pelagic phase). Endangered status following IUCN (CR = Critically Endangered; ED = Endangered; VU = Vulnerable; NT = Near threatened; LC = Least concern; DD = Data deficient) or Brazilian Red List (ET = Threatened of extinction; OT = Over-exploited). Habitat use (total species = proportion between the number of species that use one habitat on the total number of species found in the VTC; exclusive species = proportion between the number of species that use exclusively one habitat on the total number of species found in this habitat) (RS = reefs; RH = rhodolith beds; WC = water column; SD = sand).

Fish assemblages did not differ significantly between seamounts and islands in regards to geographic distribution of the species (Chi-squared test; p = 0.568), trophic habit (Chi-squared test; p = 0.257) or spawning mode (Chi-squared test; p = 0.536) (Fig. 3). However, the islands shelter almost twice the number of endemic species than the seamounts, whereas seamounts showed a higher number of endangered species (Fig. 3).

Reef habitats showed higher species richness than other habitats, sheltering 70% of all species at islands and 58% at seamounts. The number of exclusive species found in each habitat differed significantly between islands and seamounts (Chi-squared test; p = 0.003). At seamounts, exclusive species for the water column were three times more numerous than that of rhodolith beds, while at islands reefs held six times more exclusive species than rhodolith beds (Fig. 3).

## Discussion

Seamounts of the VTC have a relatively high fish diversity that is, overall, similar or higher than those at several oceanic islands in the Atlantic Ocean [63,64] or in other biogeographical provinces such as Caribbean Sea [9,65,66], Tropical Eastern Pacific [67], Southwestern Indian Ocean [68] and the northwestern Hawaiian seamount chain [69]. The recent increase in the number and scope of scientific diving expeditions, which take advantage of breathing-gas mixes and rebreathers, is improving the biodiversity assessment of mesophotic reefs at remote oceanic spots and is leading to many important discoveries. So far, scientific diving on the VTC seamounts increased the list of known fish species by 80% (an increase of 85% when considering fishery data) and extended the known depth range for 64 species. Additionally, almost all species recorded on the VTC seamounts have not been listed in worldwide reviews of seamount fish fauna [70] and the present database increases by more than 25% the number of fish species known to inhabit seamounts [71].

**Fig 4:**
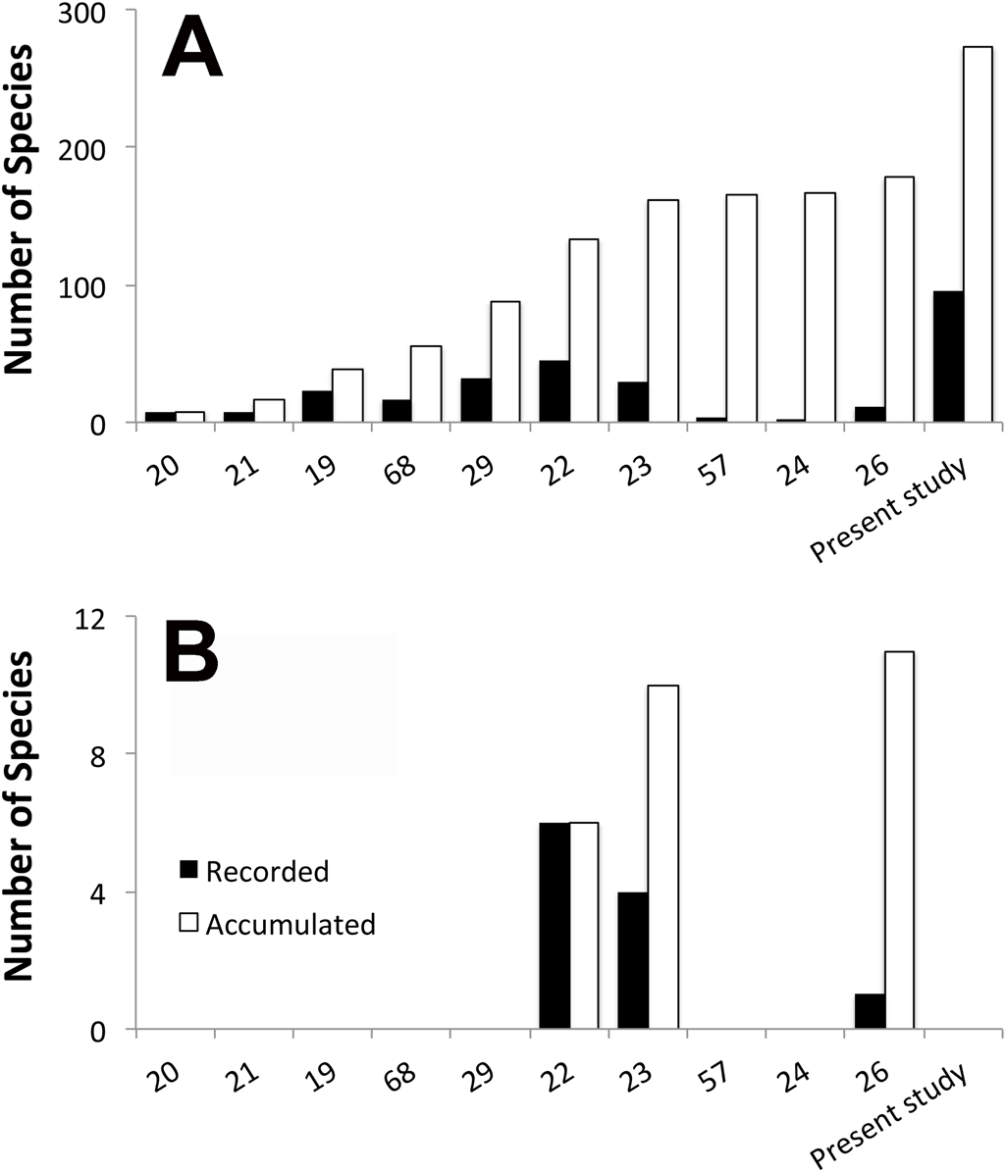
Number of species presented per published manuscripts about Vitória-Trindade Chain ichthyofauna. A) General fish species. B) Endemic fish species.

The endemism level of reef fishes at the VTC (7% for the entire chain and 9.6% for the islands only) is high compared to other Atlantic oceanic localities [63]. VTC endemics are also important for southwestern Atlantic, since they represent about 11% of the total number of endemic reef fishes found in the Brazilian Province. Thus, the VTC can be considered a biodiversity hotspot where the number of known endemic species is still increasing with additional collections and taxonomic studies (Fig. 4) [23,26].

Increase in maximum depth and presence of fishes previously considered as Trindade endemics on several seamounts raise some interesting evolutionary hypothesis about adaptation and speciation processes for reef fishes in this region. Populations of typically shallow-water herbivores (*e.g*., *Acanthurus bahianus* and *Stegastes fuscus*) and invertivores fish (e.g., *Halichoeres poeyi* and *H. penrosei*) which were unexpectedly found on mesophotic seamount’s summits (55–70 m deep) can be evolving in isolation since the last oceanic transgression 20 Ky B.P. or suffering strong selection towards life in deeper habitats. Moreover, before the present study, endemism at Trindade and Martin Vaz islands was considered to be a result of allopatry between insular and continental populations [22]. As the islands are positioned at the extreme of the ridge and hold the only strictly-shallow habitats of the chain (tidepools, sandy beaches and rocky shores), a considerable portion of their species, especially the strictly-shallow water dwellers (*e.g*., Blenniidae, Gobiesocidae and Labrisomidae) could actually have colonized these islands via stepping stones in regressive periods of low sea level [22,25,26,28].

The presence of endemic species over the VTC seamounts also calls attention for a strong ecological barrier between the continental shelf and the westernmost oceanic mounts, a barrier that can bolster ecological and/or parapatric speciation [72]. Environmental differences among continental shelf, seamounts and islands may be strong drivers for natural selection and speciation and, in addition, the Brazil Current (BC), which flows south along the outer shelf and slope, may also intensify this ecological barrier, mainly constraining larval flow between continental coast and seamounts. Most of species hindered by such constraint are dependent on specific shallow-water habitats (*e.g*. tidepools, seagrass beds, mangroves) or on demersal connectivity such as cross-shelf gradients [73]. For instance, several fish groups, such as haemulids, gobiids, and lutjanids, do not readily cross this environmental barrier. On the other hand, despite differences in habitat diversity and fish composition between seamounts and islands (only 40% of compositional overlap), their similarities in assemblage structure (regarding geographic distribution of species, trophic habit and spawning mode) suggest similar equilibrium mechanisms for community organization and maintenance.

Genetic analyses supported the remarkable singularity of the VTC environments, showing that some of those VTC endemics, such as *H. rubrovirens* and *S. rocha*, are relict [74,75], or paleo-endemic species [76]. This suggests that old evolutionary lineages may have been preserved on the VTC seamounts and islands while continental lineages evolved in different species or became extinct. A recent study shows that such refugia contributed to current patterns of biodiversity distribution in the Indo-Pacific region [77]. Brazilian seamounts are hypothesized as refuges for scleractinian corals during the last ice ages, with further re-colonization of the continental shelf during the last transgression [78,79]. Conversely, the paleoendemic reef fishes seem to have remained isolated on the VTC. Such relict lineages deserve special attention for conservation efforts since they carry important and unique genetic and evolutionary information [80].

Despite the ubiquity of large carnivorous fishes such as groupers, jacks and barracudas on the VTC seamounts (authors’ personal observations), indications of overfishing are very evident, especially at the Trindade and Martin Vaz islands [25,57]. Unmanaged fishing activities done by domestic and foreign fishing vessels have been regularly recorded at VTC [57] albeit the vulnerability of oceanic islands and seamounts to fishing activities is well known [56,59]. On seamounts, little regeneration is observed even after trawling over deep-coral ecosystems has been discontinued, and full habitat regeneration is estimated to require centuries [81]. Apparently, highly destructive trawling activities have not yet occurred in the VTC like those conducted at seamounts off N-NE Brazil [58], but VTC seamount chain is presently lacking legal protection.

Carbonate’s extraction is an emergent and highly destructive activity threatening the VTC seamounts, and has been already conducted at Davis Seamount between 2009 and 2011 [82]. This industry aims at extracting the slow-growing rhodoliths to produce fertilizers for sugar cane and other agricultural commodities upon which Brazil’s economy is dependent [82]. This activity thus directly threatens almost half of the species listed in the present study. Besides mining of carbonates, other possible threats to VTC biodiversity are the extraction of iron-manganese [83] and cobalt-rich crusts in deeper areas of the slope and seabed [84]. These mining activities tend to destroy the sea bed and its associated biodiversity [85], representing major threats to the VTC, similarly to the situation in some areas of the Pacific [86–88].

While hindering seabed mining based on National-level permits, the fact that some VTC seamounts are still Areas Beyond National Jurisdiction (ABNJ) challenges the management of fisheries and other natural resources. However, UN General Assembly call upon states and Regional Fisheries Management Organizations (RFMOs) to protect Vulnerable Marine Ecosystems (VMEs) in ABNJ—including seamounts—from destructive fishing practices. The area of the VTC outside the Brazilian EEZ is presently within the area requested by Brazil for continental shelf extension. If accepted by the Commission on the Limits of the Continental Shelf (CLCS) of the United Nation Convention of the Law of the Sea (UNCLOS), Brazil would not only have the full right to exploit living and non-living (mineral) resources, but also the duty of protecting its unique biodiversity. The establishment of Marine Protected Areas is a recommended measure for the region, following the example of many countries that have already set aside seamounts within their EEZs for protection (such as Australia, New Zealand and UK—Chagos Archipelago [54,89,90]). Additionally, programs and actions for monitoring, evaluating and managing fishery resources in the VTC region are urgently needed [91]. One option is the application of the Brazilian National Satellite Tracking Project (PREPS), which monitors fishing boats over 15 m of length. This program should be expanded to include the smaller 10 m-vessels that operate in critical areas such as the VTC and elsewhere in Brazil [57].

The VTC is possibly among the most endangered and important oceanic regions of the world (based in criteria detailed in [92,93]), and is an important ecological corridor and an evolutionary hotspot that has a vital role in the maintenance of the biodiversity of the remote Trindade and Martin Vaz islands. However, if not managed effectively, it is possible that several peculiarities of this diverse and extraordinary oceanic system will soon be permanently lost. Immediate action at the VTC must be included in the priority agenda for environmental conservation in Brazil, the country that owns and claims additional rights and duties over the unique Vitória-Trindade Seamount Chain.

## Methods

### Fish database

Primary data was acquired during three scientific diving expeditions to the VTC seamounts and islands, in 2009 (12–26 March) and 2011 (3–26 February and 1–18 April). These expeditions covered the photic and upper mesophotic zones (0–120 m depth) of the two islands and eight seamounts: Almirante Saldanha, Vitória, Eclaireur, Jaseur, “Jaseur East” (Columbia Bank in [35]), Davis, Dogaressa and Columbia (Fig. 1). Sampling included visual, video and photo records, as well as collection of voucher specimens by divers (hand nets and spear-guns in April 2011) using technical open-circuit SCUBA or closed-circuit rebreathers (Megalodon) with mixed-gases (TRIMIX and EAN). Fish collection at all localities along the VTC seamounts and islands and collection of the protected species *Elacatinus figaro* at the same sites were authorized by the Brazilian Environmental Agency [Instituto Chico Mendes de Conservação da Biodiversidade (SISBIO 12786–1 and 20880–2)]. Ten hours of video from two remotely operated underwater vehicles (ROVs) (Seabotix LBV 150S2 and Video Ray SCOUT) were used for habitat descriptions and provided extra faunal records.

Primary data from fishery surveys (surface longline, bottom longline, midwater trawling and angling activities; see [31–34,91,94]) were incorporated in the database. Fishery sampling was performed over eight volcanic mounts (Vitória, Eclaireur, Besnard, Montague, Jaseur, Davis, Dogaressa, Columbia and Trindade) during scientific cruises of the REVIZEE Program and to a much lesser extent TAMAR/ICMBio monitoring assessments. REVIZEE stands for Program for the evaluation of the sustainable potential of living resources of the exclusive economic zone, a government-supported program conducted between 1994 and 2006. Only records in waters shallower than 120 m were used here. Information about sampling effort and general characteristics of the sites surveyed are provided in Table 2.

**Table 2:** Summary of sampling effort, data sources and sampling site characteristics of the Vitória-Trindade Seamount Chain, southwestern Atlantic.

Site	Summit area (km^2^)	*Substrate type*	*Sampling techniques*	*Dive depth range (m)*	*Number of dives*	*Primary data type*	*References*
**Vitória**	1184	RH/RS/PR/SD	DIV/ROV/ ZEE/CF	35–120	38	VO/PHO/VID/VIS/ OB/ZEE	Primary data; [94]
Almirante Saldanha	37	RH/RH/SD	DIV	66	3	PHO/VID/VIS/	Primary data; [94]
Besnard	1978	Unknown	ZEE	-	-	ZEE	Primary data; [33,94]
Montague	124	Unknown	ZEE/CF	-	-	ZEE/OB	Primary data; [29,94]
Jaseur	89	RH/RS/SD	DIV/ROV/ ZEE	62	5	PHO/VID/VIS/ ZEE/OB	Primary data
Jaseur East	99	RH/RS/SD	DIV	62	20	VO/PHO/VID/VIS/	Primary data
Eclaireur	6,4	RH/SD	DIV/ROV	71	6	PHO/VID/VIR	Primary data; [33]
Davis	1002	RH/RS/SD	DIV/ROV/ ZEE	17–57	46	VO/PHO/VID/VIR/ UVC/ZEE	Primary data; [33]
Dogaressa	80,5	RH/SD	DIV/ZEE	65	14	VO/PHO/VID/VIR/ ZEE	Primary data; [29]
Columbia	36,5	RH//PR/SD	DIV/ZEE	84	3	PHO/VID/VIR/ZEE	Primary data; [29,33]
Trindade	85	RH/RR/PR/SD	DIV/ROV/ ZEE	0–85	200	VO/PHO/VID/VIR/ UVC/ZEE	Primary data; [19–24,26,28,57,104]
Martin Vaz	24	RR/SD	DIV/ROV	0–30	20	VO/PHO/VID/VIR/ UVC	Primary data; [23,24,26,57]

Type of substrate: RS—Reef structure (carbonatic); RR—Rocky reef; PR—patch reef; RH—Rhodolith bed; SD—Sand and unconsolidated substrate. Sampling techniques: DIV—Diving; ROV—Remote operated vehicle; CF—Commercial fishing; ZEE—REVIZEE scientific fishing. Primary data type: VO—Voucher specimen; PHO—photo record; VID—video record; VIS—*in situ* visual record; UVC—underwater visual census; ZEE—REVIZEE project record; OB—onboard observer record during commercial fishing.

Publications on the fish fauna of the seamounts are limited to the results of the 1987 Brazilian-French expedition MD55 Brazil [29,30] and REVIZEE reports [31–34,91,94]. For the islands, all earlier published material was recently reviewed by [26]. This later study includes a checklist of Martin Vaz, cited as “H.T. Pinheiro pers. comm.” that originated from a three-day, 15 diving hours expedition in February 2007. New records for species not covered in [26] were obtained by ACF (pers. comm.) and the above-mentioned recent scientific expeditions.

A species list, with comments on selected biological features was built using all available records. Information was broken down by seamount/island and was given in order of record reliability: deposited vouchers, literature, photo/video records, unpublished records (REVIZEE and fishery surveys) and visual records (S1 Annotated Checklist). The VTC fish database is also available at https://marinebiodiversity.lncc.br (access number knb.9.2), a public and easily accessible online database for marine biodiversity.

Traits of each species (spawning mode, trophic guilds, depth range, geographic distribution and conservation status) were compiled from the literature [62,95–102] and were complemented by the authors´ observations. The habitats in which species were found (reefs, rhodolith beds, water column or sand) were assigned for each recorded occurrence. A short video entitled “Fishes of the Vitória-Trindade Chain”, showing the various habitats of VTC seamounts, is available at http://youtu.be/ZsV3AkDvvvE (a trailer of the movie is also available as S1 Movie). Differences between assemblages composition at seamounts and islands were tested by Chi-squared tests in respect to species traits [103]. Summit area, displayed in Table 2, was calculated in the program ArcGis based on the 150 m isobaths from nautical charts (Diretoria de Hidrografia e Navegação—DHN: 20 and 21).

### Ethics Statement

The collection of fishes during the April 2011 expedition is in accordance with the ethical principles for animal experimentation and approved by the Ethics Committee for the Use of Animals of the Universidade Federal do Espírito Santo (CEUA-UFES 017–2009). There were no collections in the March 2009 and February 2011 expeditions. Fish collection at all localities along the VTC seamounts and islands and collection of the protected species *Elacatinus figaro* at the same sites were authorized by the Brazilian Environmental Agency, Instituto Chico Mendes de Conservação da Biodiversidade (SISBIO 12786–1 and 20880–2 to JCJ).

## Supporting Information

S1 Annotated Checklist Annotated checklist of the fishes from the Vitória-Trindade Chain, southwestern Atlantic. (PDF)

S1 Movie Trailer of the movie “Fishes of the Vitória-Trindade Seamount Chain”. (MP4)
