# Supplementary material for: Fish Biodiversity of the Vitória-Trindade Seamount Chain, Southwestern Atlantic: An Updated Database
Source: PLoS One. 2015 Mar 4;10(3):e0118180. doi: 10.1371/journal.pone.0118180 (PMC4349783; doi:10.1371/journal.pone.0118180)
Supplement: S1 Annotated Checklist — (PDF) [file pone.0118180.s001.pdf]

PLoS ONE - Supporting Information - Accepted: December 2014

Fish biodiversity of the Vitória-Trindade Seamount Chain, southwestern Atlantic: an updated database

Hudson T. Pinheiro, Eric Mazzei, Rodrigo L. Moura, Gilberto M. Amado-Filho, Alfredo Carvalho-Filho, Adriana C. Braga, Paulo A. S. Costa, Beatrice P. Ferreira, Carlos Eduardo L. Ferreira, Sergio R. Floeter, Ronaldo B. Francini-Filho, João Luiz Gasparini, Raphael M. Macieira, Agnaldo S. Martins, George Olavo, Caio R. Pimentel, Luiz A. Rocha, Ivan Sazima, Thiony Simon, João Batista Teixeira, Lucas B. Xavier, Jean-Christophe Joyeux

Annotated checklist of the fishes from the Vitória-Trindade Chain, southwestern Atlantic

Below is an annotated checklist of the fishes known to occur at VTC. Orders and families follow Nelson [1], Epinephelidae follows Craig & Hastings [2] and Craig et al. [3], and Labridae follows Westneat & Alfaro [4]. Species nomenclature follows Eschmeyer [5]. Genera and species are listed in alphabetical order. New records for VTC seamounts and islands are marked with § and for global depth range extensions with ‡ after the species' name. Information for each species includes: **habitat type.** indicating the habitat(s) where the species has been recorded at VTC [reef structure (RS), rhodolith beds (RH), sand/mud (SD) and water column (WC)]; **global distribution.** [Brazilian province (Br) (according to [6]), Central Atlantic (CA), Circumglobal (CG), Circumtropical (CT), Eastern Atlantic (EA), Eastern Pacific (EP), Indo-Pacific (IP), Mediterranean (M), Southern Caribbean (SCa), Southwestern Atlantic (SW), Trans-Atlantic (TA), Trindade endemic (TE), Vitória-Trindade Chain endemic (VTC), Western Atlantic (WA), Western Central Atlantic (WC); **conservation status.** according to IUCN Red List [Least concern (LC), Not appraised (NA), Near threatened (NT), Data deficient (DD), Critically endangered (CR), Endangered (ED), Vulnerable (VU)], and/or Brazilian Red List [Threatened of Extinction (ET), Over-exploited (OT)], and economic importance [Commercial target (CT)]; **depth range.** from information available in the literature (L) and based on our records (O) (presented in meters); **regional distribution.** (Islands, Seamounts, Islands-Seamounts); **location record.** [Almirante Saldanha Seamount (ALSAL), Besnard Seamount (BESN), Columbia Seamount (COL), Davis Seamount (DAV), Dogaressa Seamount (DOG), Eclaireur Seamount (ECL), Jaseur Seamount (JAS), Jaseur East Seamount (JAE; *i.e.*, Columbia bank in [7]), Martin Vaz Archipelago (MAR), Montague Seamount (MON), Trindade Island (TRI), Vitória Seamount (VIT)]. In the species accounts, seamounts and islands are listed from closest (Vitória) to farthest (Martin Vaz) from the continental shelf; **Type of record.** voucher specimens in ichthyologic collections (CIUFES, MBML-Peixes, MZUSP and ZUEC-PIS), Literature records [numbered], Fishing operation (FIS); REVIZEE unpublished data (ZEE), Photo records (PHO), Video records (VID), Visual sighting (VIS).

Orectolobiformes

Ginglymostomatidae

Ginglymostoma cirratum (Bonnaterre 1788) §

RS; CG; DD/ET; L (0-130); O (5-67); Seamounts-Islands: VIT (VID), JAE (PHO), DOG (ZEE), TRI [8], MAR (PHO); Figure S. 1.

The only shark species recorded during dive surveys on VTC seamounts, and often caught during fishing activity at Trindade Is., which includes recreational angling fishing and spearfishing [9].

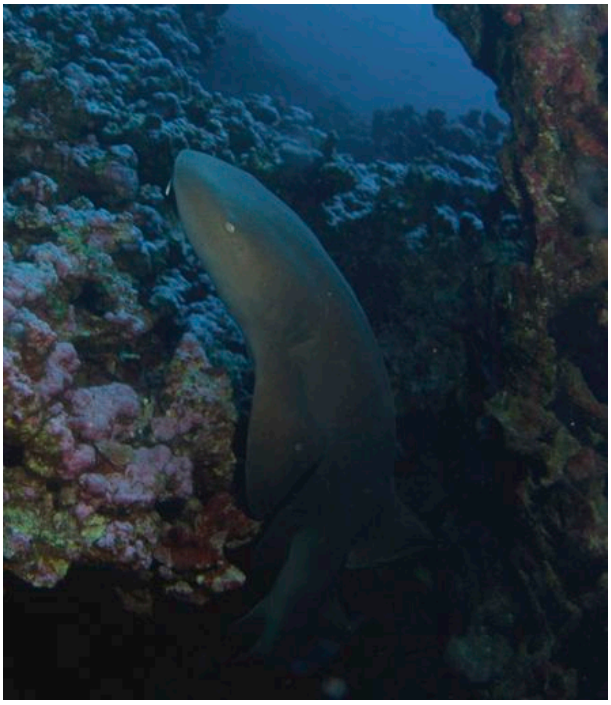

Figure S. 1. *Ginglymostoma cirratum*, underwater photo at Davis seamount, by R. L. Moura.

Rhincodontidae

Rhincodon typus Smith 1828

WC; CG; VU/ET; L (0-700); O (8); Islands: TRI [10].

Lamniformes

Alopiidae

Alopias superciliosus Lowe 1841 §

WC; CT; VU; L (1-500); O (56); Seamounts: BESN (ZEE).

Lamnidae

Carcharodon carcharias (Linnaeus 1758)

RS/RH/WC; CG; VU; L (0-1280); O (—); Islands: TRI [11].

Isurus oxyrinchus Rafinesque 1810 §

WC; CG; VU; L (1-740); O (41-82); Seamounts: VIT (ZEE), BESN (ZEE).

Isurus paucus Guitart 1966 § ‡

WC; CG; VU; L (200), O (41-66); Seamounts: BESN (ZEE).

Carcharhiniformes

Triakidae

Mustelus canis (Mitchill 1815) §

RS/RH; WA; NT; L (800); O (72-350); Seamounts: BESN (ZEE), ECL (ZEE), JAS (ZEE), MONT (ZEE).

Carcharhinidae

Carcharhinus falciformis (Müller & Henle 1839) §

WC; CG; NT; L (18-4000); O (56); Seamounts-Islands: VIT (FIS), BESN (ZEE), MONT (FIS), TRI (FIS); Figure S. 2.

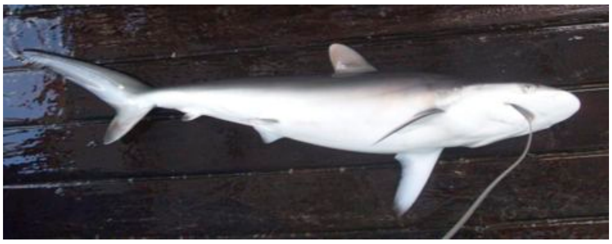

Figure S. 2. *Carcharhinus falciformis* from Montague seamount, photo by E. Mazzei.

Carcharhinus galapagensis (Snodgrass & Heller 1905)§

WC; CT; NT; L (1-286); O (—); Islands: TRI (FIS).

Carcharhinus leucas (Müller & Henle 1839) §

WC; CG; NT; L (1-152); O (—); Seamounts-Islands: MONT (FIS), TRI (FIS).

Carcharhinus limbatus (Müller & Henle 1839)

RS/RH/WC; CG; NT; L (0-64); O (—); Islands: TRI [12].

Carcharhinus longimanus (Poey 1861) §

WC; CG; VU/ET; L (0-230); O (—); Seamounts-Islands: VIT (FIS), TRI (FIS); Figure S. 3.

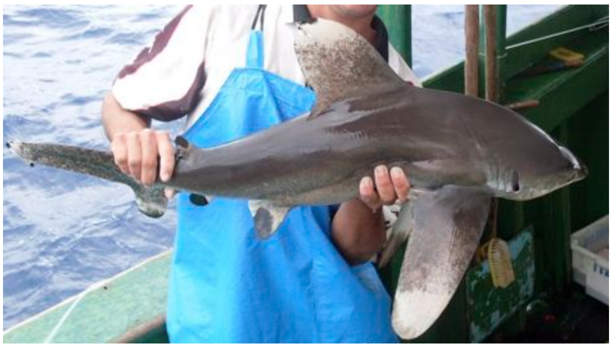

Figure S. 3. *Carcharhinus longimanus* from Vitória seamount, photo by E. Mazzei.

Carcharhinus obscurus (Lesueur 1818)

RS/RH/WC; CG; VU; L (0-400); O (—); Islands: TRI [12].

Carcharhinus perezi (Poey 1876)

RS/RH/WC; WA; NT; L (1-65); O (1-35); Islands: TRI (ZUEC-PIS 2766, 2767) [8], MAR [13]; Figure S. 4.

Large individuals were recorded regularly in the 80s and 90s. They apparently became rare in the 2000s, probably due to recreational and commercial fishing activities [9], which include commercial and recreational ones. Still commonly sighted as single individuals, but a group of ~15 individuals was sighted on 23 March 2007 by H. T. Pinheiro, at 2-8 m depth at the Noroeste Bay, Trindade Island.

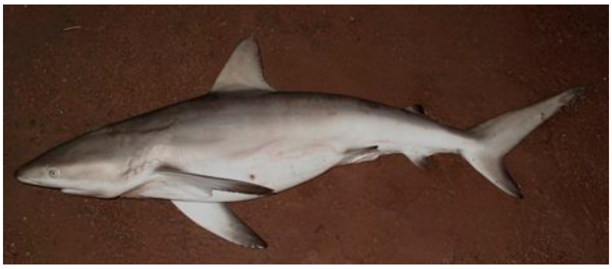

Figure S. 4. *Carcharhinus perezii* from Trindade Island, photo by H. T. Pinheiro.

Carcharhinus plumbeus (Nardo 1827) §

WC; CG; VU; L (1-280); O (56); Seamounts-Islands: BESN (ZEE), TRI (FIS).

Carcharhinus signatus (Poey 1868) §

WC; TA; VU/ET; L (40-600); O (56); Seamounts: BESN (ZEE).

Galeocerdo cuvier (Péron & Lesueur 1822) §

RS/RH/WC; CG; NT; L (0-370); O (71); Seamounts-Islands: VIT (PHO), BESN (ZEE), ECL (ZEE), DOG (CT-CIUFES 0159), TRI [12], MAR (FIS).

Prionace glauca (Linnaeus 1758)

WC; CG; NT/ET; L (1-350); O (50); Seamounts-Islands: ALSAL [14], VIT [14], BESN [14], MONT [14], TRI [9].

The blue shark is one of the most commonly targeted species by commercial fisheries with surface longlines [9].

Sphyrnidae

Sphyrna lewini (Griffith & Smith 1834) §

RS/WC; CG; ED/OT; L (0-512); O (45); Seamounts-Islands: BESN (ZEE), TRI (FIS), MAR (PHO); Figure S. 5.

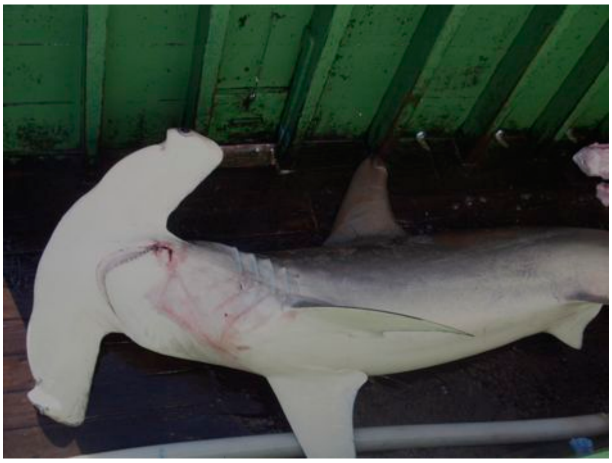

Figure S. 5. *Sphyrna lewini* from adjacent to the Vitória seamount, photo by E. Mazzei.

Sphyrna zygaena (Linnaeus 1758) §

WC; CG; VU; L (20-200); O (45-71); Seamounts: BESN (ZEE).

Squaliformes

Squalidae

Squalus mitsukurii Jordan & Snyder 1903

RS; CG; DD; L (20-495); O (246); Seamounts: BESN [15].

Myliobatiformes

Dasyatidae

Dasyatis centroura (Mitchil 1815) §

RS/RH; TA/M; LC; L (3-270); O (45); Seamounts: VIT (VID).

Dasyatis guttata (Bloch & Schneider 1801) §

RS/RH; TA/M; LC; L (3-270); O (55); Seamounts: JAE (VIS).

Pteroplatytrygon violacea (Bonaparte 1832) §

WC; CG; LC; L (1-381); O (48-77); Seamounts: VIT (ZEE).

Myliobatidae

Aetobatus narinari (Euphrasen 1790) §

WC; CT; NT; L (1-80); O (15-55); Seamounts-Islands: JAE (PHO), TRI [10].

Mobula japanica (Müller & Henle 1841) §

WC; CT; NT; L (0-200); O (-); Seamounts: MONT (FIS); Figure S. 6.

There are only two published records for *M. japanica* in the western Atlantic off Brazil, one of them based on specimens caught off eastern and southern Brazil [16], and the other based on sightings at Saint Paul and Saint Peter Archipelago in central equatorial Atlantic [17]. Thus, this is the first record for the spinetail mobula near Trindade Island. Off Brazil, this ray occurs mostly at oceanic and coastal islands [16].

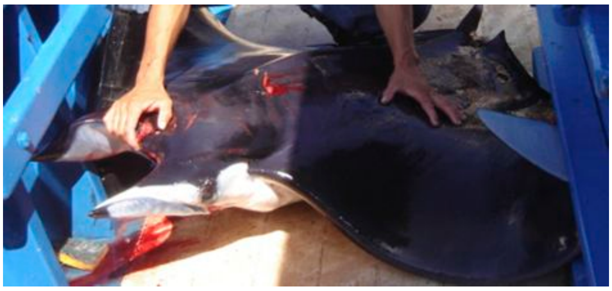

Figure S. 6. *Mobula japanica* from Montague seamount, photo by E. Mazzei.

Anguilliformes

Moringuidae

Moringua edwardsi (Jordan & Bollman 1889)

RS; WA; LC; L (—); O (3); Islands: TRI [18].

Chlopsidae

Chlopsis bicolor Rafinesque 1810 ‡

RH; TA/M; L (80-365), O (60); NA; Islands: TRI [19].

Kaupichthys hyoproroides (Strömman 1896)

RH; CT; NA; L (1-95); O (30); Islands: TRI (CIUFES 2320, 2335, 2419) [20]; Figure S. 7.

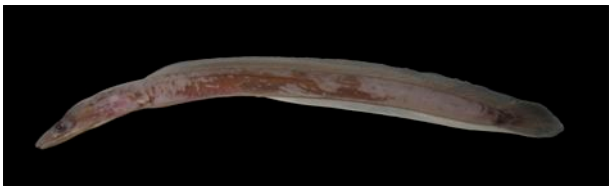

Figure S. 7. *Kaupichthys hyoproroides*, CIUFES 2205, 60mm TL, from Trindade Island, photo by T. Simon.

Muraenidae

Channomuraena vittata (Richardson 1845) §

RH; CT; NA; L (5-100); O (63); Seamounts-Islands: DOG [19], TRI (PHO); Figure S. 8.

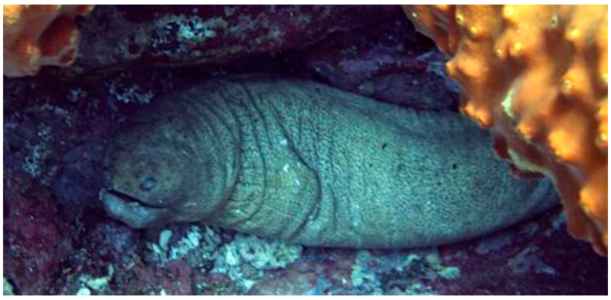

Figure S. 8. *Channomuraena vittata*, underwater photo at Trindade Island, photo by J. B. Mendonça Junior.

Echidna catenata (Bloch 1795)

RS; TA; NA; L (0-20); O (0-18); Islands: TRI (CIUFES 1256, 2637; MZUSP 55034.0; ZUEC-PIS 2699, 2831) [21]; Figure S. 9.

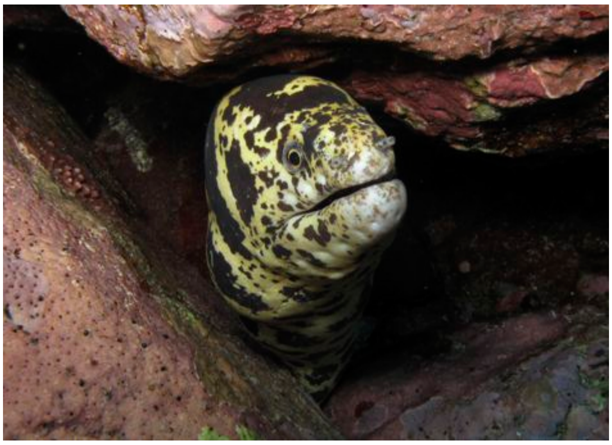

Figure S. 9. *Echidna catenata*, underwater photo at Trindade Island, by L. B. da C. Xavier.

Enchelycore carychroa Böhlke & Böhlke 1976 §‡

RS; TA; NA; L (1-54), O (1-65); Seamounts-Islands: DOG (CIUFES 2051), TRI (CIUFES 2241, 2243) [10].

Enchelycore nigricans (Bonnaterre 1788)

RS; TA; NA; L (0-100); O (0-35); Islands: TRI (CIUFES 3135; ZUEC-PIS 2656, 2698) [11]; Figure S. 10.

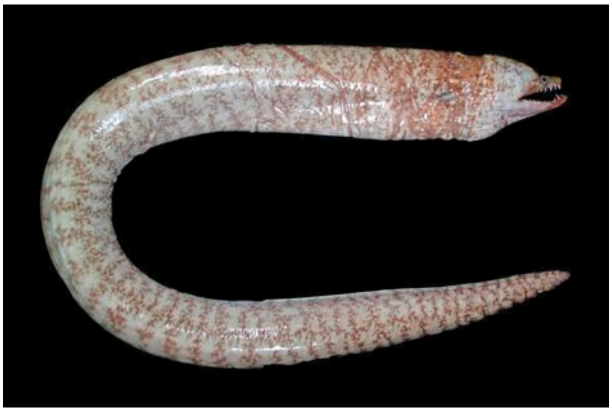

Figure S. 10. *Enchelycore nigricans*, CIUFES 3135, 76 cm TL, from Trindade Island, photo by H. T. Pinheiro.

Gymnothorax funebris Ranzani 1839 §

RS/RH; CT; NA; L (0-383); O (45-81); Seamounts: VIT (VID), JAS [19], MONT [19], JAE (VID), DAV [19], DOG [19].

This species is present along the Brazilian coast and at all oceanic islands of the Brazilian Province [22] except for Trindade and Martin Vaz. Its absence there probably is not due to its dispersal potential.

Gymnothorax maderensis (Johnson 1862) §

RS/RH; TA; NA; L (85-357); O (85-285); Seamounts: BESN (ZEE), DOG (ZEE), COL (ZEE).

Gymnothorax miliaris (Kaup 1856) §

RS/RH; TA; NA; L (0-260), O (0-71); Seamounts-Islands: ECL (PHO), DOG (ZUEC-PIS 8281), TRI (ZUEC-PIS 2693, 2801) [18], MAR [20]; Figure S. 11.

At Trindade, this species display three color morphs: the common dark color 'miliaris' and two other pale morphs, 'xavopicta' and 'irregularis' [18].

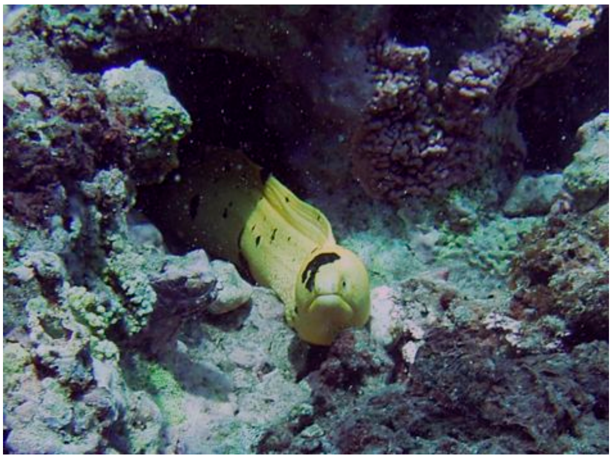

Figure S. 11. *Gymnothorax miliaris*, underwater photo at Trindade Island, by T. Simon.

Gymnothorax moringa (Cuvier 1829) §

RS/RH; TA; NA; L (0-200); O (85-189); Seamounts-Islands: VIT (ZEE), BESN (ZEE), ECL (ZEE), JAS (VID); MONT (ZEE), DAV (ZEE), DOG (ZEE), COL [19], TRI (CIUFES 2242, ZUEC-PIS 2832) [11], MAR [13]; Figure S. 12.

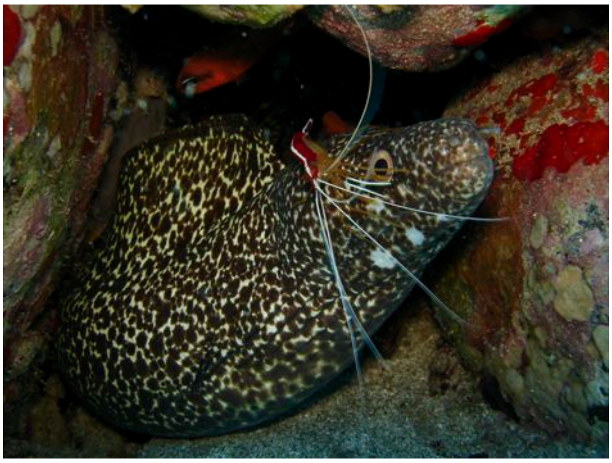

Figure S. 12. *Gymnothorax moringa*, underwater photo at Trindade Island, by R. M. Macieira. A scarlet-striped cleaning shrimp *Lysmata grabhami* (Gordon 1935) is cleaning the moray.

Gymnothorax ocellatus Agassiz 1831 §

RS/RH; WA; NA; L (1-160); O (37-105); Seamounts: VIT [19], BESN (ZEE), DOG [19], COL [19].

Gymnothorax polygonius Poey 1875 §‡

RS/RH; TA; NA; L (10-256), O (50-354); Seamounts-Islands: BESN (ZEE), ECL (ZEE), DAV (ZEE), DOG (ZEE), COL (ZEE), TRI [18].

Gymnothorax vicinus (Castelnau 1855) §‡

RS/RH; TA; NA; L (0-145), O (97-186); Seamounts: BESN (ZEE), JAS (ZEE), MONT (ZEE).

Monopenchelys acuta (Parr 1930) ‡

RH; CT; LC; L (13-45), O (30-81); Seamounts-Islands: MONT [19], TRI (CIUFES 2204, 2336) [20]; Figure S. 13.

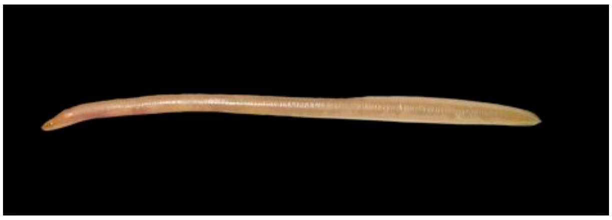

Figure S. 13. *Monopenchelys acuta*, CIUFES 2204, 84mm TL, from Trindade Island, photo by T. Simon.

Ophichthidae

Myrichthys breviceps (Richardson 1848)

RS/RH/SD; WA; NA; L (0-30); O (3-18); Islands: TRI [11], MAR [13]; Figure S. 14.

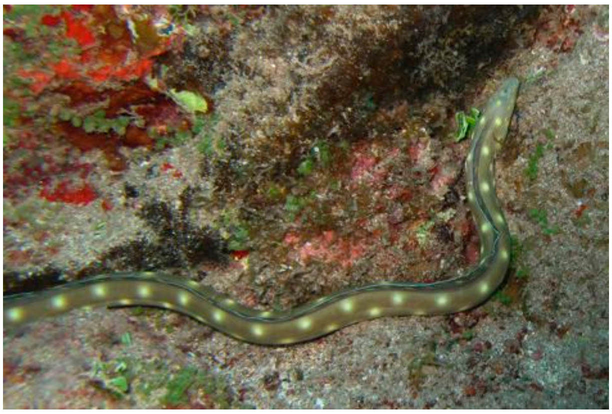

Figure S. 14. *Myrichthys breviceps*, underwater photo at Trindade Island, by H. T. Pinheiro.

Myrophis sp.

SD; Islands: O (—); TRI [18].

Ophichthus gomesi (Castelnau 1855)

RH/SD; WA; NA; L (1-450); O (50-100); Seamounts: JAE [19], COL [19].

Ophichthus ophis (Linnaeus 1758) §‡

RH/SD; TA; NA; L (3-50 m), O (5-66 m); Seamounts-Islands: ALSAL (PHO), TRI [18]; Figure S. 15.

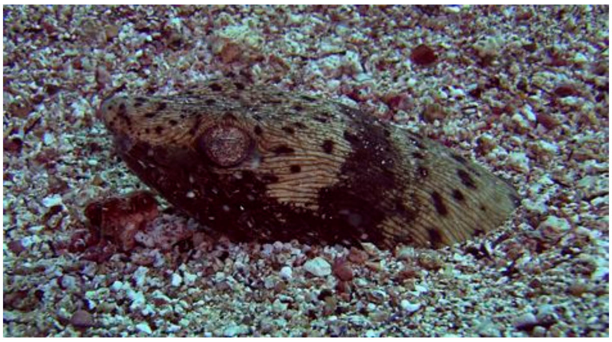

Figure S. 15. *Ophichthus ophis*, underwater photo at Trindade Island, by T. Simon.

Pseudomyrophis frio (Jordan & Davis 1891)

SD; Br; NA; L (108); O (100); Seamounts: COL [19].

Muraenesocidae

Cynoponticus savanna (Bancroft 1831)

RH/SD; WA; NA; L (0-100); O (50-63); Seamounts: DOG [19].

Congridae

Ariosoma opisthophthalmum (Ranzani 1839)

RH/SD; Br; NA; L (110-600), O (50); Seamounts: MONT [19].

Heteroconger sp.§

SD; NA; L (—), O (84); Islands: TRI (PHO).

Clupeiformes

Clupeidae

Harengula jaguana Poey 1865

WC; WA; NA; L (1-22); O (1-5); Islands: TRI (CIUFES 1320, 2658; ZUEC-PIS 2701, 2760, 2761, 6314) [12]; Figure S. 16.

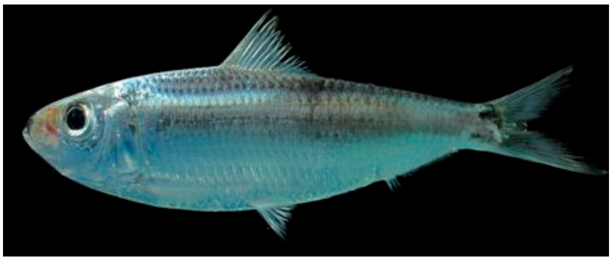

Figure S. 16. *Harengula jaguana*, CIUFES 3113, 155mm TL, from Trindade Island, photo by H. T. Pinheiro.

Stomiiformes

Gonostomatidae

Diplophos taenia Günther 1873 §

WC; CT; NA; L (15-1594); O (49-68); Seamounts: MONT (ZEE), DAV (ZEE).

Manducus maderensis (Johnson 1890) §

WC; TA; NA; L (10-800); O (49); Seamounts: DAV (ZEE).

Sternoptychidae

Maurolicus stehmanni Parin & Kobyliansky 1993 §

WC; SW; NA; L (0-200); O (72); Seamounts: VIT (ZEE).

Phosichthyidae

Pollichthys mauli (Poll 1953) §

WC; CG; NA; L (18-1105), O (49-68); Seamounts: MONT (ZEE), DAV (ZEE).

Vinciguerria nimbaria (Jordan & Williams 1895) §

WC; CG; NA; L (20-5000); O (68); Seamounts: MONT (ZEE).

Stomiidae

Astronesthes similus Parr 1927 §

WC; WC; NA; L (0-850); O (42-49); Seamounts: DAV (ZEE).

Aulopiformes

Synodontidae

Saurida sp.

WC; O (29-72); Seamounts: VIT (ZEE), DAV (ZEE).

Synodus foetens (Linnaeus 1766)

RS; WA; NA; L (0-200); O (33-105); Seamounts: VIT [19].

Synodus intermedius (Spix & Agassiz 1829) §

RS/RH; WA; NA; L (3-320); O (85-97); Seamounts: VIT [19], DOG (VIS), COL [19].

Synodus synodus (Linnaeus 1758) §

RS/RH; TA; NA; L (0-90); O (2-35); Seamounts-Islands: DAV (PHO), TRI (ZUEC-PIS 2770) [18]; Figure S. 17.

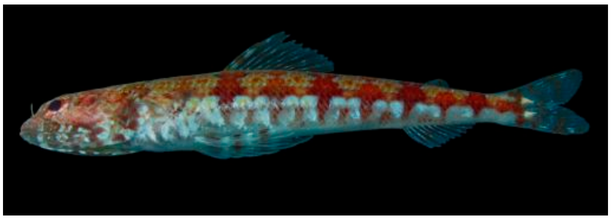

Figure S. 17. *Synodus synodus*, CIUFES 3121, 81.5mm TL, from Trindade Island, photo by H. T. Pinheiro

Trachinocephalus myops (Forster 1801)

RH/SD; CG; NA; L (0-400); O (10-35); Islands: TRI [18].

Myctophiformes

Myctophidae

Ceratoscopelus warmingii (Lütken 1892) §

WC; CG; NA; L (20-2014); O (68); Seamounts: MONT (ZEE).

Diaphus brachycephalus Tåning 1928 §

WC; CG; NA; L (35-1480), O (68); Seamounts: MONT (ZEE).

Diaphus dumerilii (Bleeker 1856) §‡

WC; TA; NA; L (50-1141); O (49-68); Seamounts: MONT (ZEE), DAV (ZEE).

Diaphus fragilis Tåning 1928 §

WC; CT; NA; L (15-1313); O (68); Seamounts: MONT (ZEE).

Diaphus garmani Gilbert 1906 §‡

WC; CT; NA; L (100-2091); O (49); Seamounts: DAV (ZEE).

Diaphus lucidus (Goode & Bean 1896) §

WC; CT; NA; L (20-2999); O (68); Seamounts: MONT (ZEE).

Diaphus perspicillatus (Ogilby 1898) §

WC; CT; NA; L (0-1500); O (49-68); Seamounts: MONT (ZEE), DAV (ZEE).

Diaphus problematicus Parr 1928 §

WC; CT; NA; L (40-820); O (68); Seamounts: MONT (ZEE).

Diaphus splendidus (Brauer 1904) §

WC; CT; NA; L (0-4000); O (68); Seamounts: MONT (ZEE).

Hygophum reinhardtii (Lütken 1892) §

WC; CG; NA; L (10-1105); O (68); Seamounts: MONT (ZEE).

Lepidophanes guentheri (Goode & Bean 1896) §

WC; TA; NA; L (40-750); O (49-68); Seamounts: MONT (ZEE), DAV (ZEE).

Myctophum affine (Lütken 1892) §

WC; TA; NA; L (10-1105); O (68); Seamounts: MONT (ZEE).

Myctophum obtusirostre Tåning 1928 §

WC; CT; NA; L (1-700); O (49-68); Seamounts: MONT (ZEE), DAV (ZEE).

Notoscopelus caudispinosus (Johnson 1863) §‡

WC; CG; NA; L (175-1000), O (68); Seamounts: MONT (ZEE).

Polymixiiformes

Polymixiidae

Polymixia lowei Günther 1859 §

RH/SD; WA; NA; L (50-600); O (103-437); Seamounts: DAV (ZEE), DOG (ZEE).

Ophidiiformes

Carapidae

Carapus bermudensis (Jones 1874)

RS/RH; WA; NA; L (1-235); O (20); Islands: TRI (CIUFES 2107, 2108, 2109, 214, 2298) [20]; Figure S. 18.

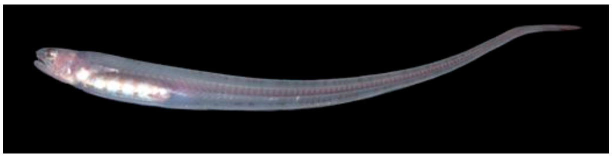

Figure S. 18. *Carapus bermudensis*, CIUFES 2114, 155.5mm TL, from Trindade Island, photo by T. Simon.

Batracoidiformes

Batrachoididae

Porichthys porosissimus (Cuvier 1829)

SD; SW; NA; L (30-200), O (34-217); Seamounts: VIT [19].

Mugiliformes

Mugilidae

Mugil curvidens Valenciennes 1836

RS/WC; WA; NA; L (0-2); O (0-1); Islands: TRI (CIUFES 1185, 1559, 1589, 2704, 131399; ZUEC-PIS 2814) [18]; Figure S. 19.

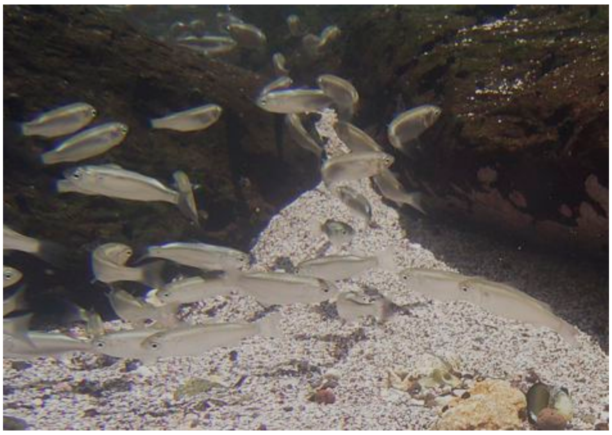

Figure S. 19. *Mugil curvidens*, underwater photo at Trindade Island, by J.-C. Joyeux. A school of juveniles in tidepools.

Beloniformes

Exocoetidae

Cheilopogon cyanopterus (Valenciennes 1847) §

WC; CG; NA; L (0-20); O (0-1); Seamounts: JAE (CIUFES 2132).

Cheilopogon furcatus (Mitchill 1815)

WC; CT; NA; L (0-20); O (—); Islands: TRI [12].

Cheilopogon heterurus (Rafinesque 1810)

WC; CG; NA; L (—); O (—); Islands: TRI [11].

Exocoetus volitans Linnaeus 1758 §

WC; CG; NA; L (0-20); O (0-1); Seamounts-Islands: JAE (CIUFES 2131), TRI [11].

Hirundichthys affinis (Günther 1866)

WC; TA/IP; NA; L (—); O (—); Islands: TRI [12].

Parexocoetus hillianus (Gosse 1851) §

WC; WA; NA; L (—); O (0-1); Seamounts: JAE (CIUFES 2130).

Hemiramphidae

Hemiramphus brasiliensis (Linnaeus 1758) §

WC; TA; NA; L (0-5); O (0-2); Islands: TRI (ZUEC-PIS 2813) [8], MAR (VIS); Figure S. 20.

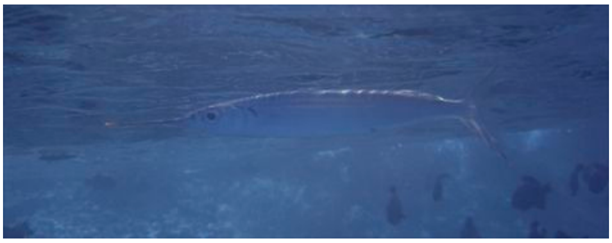

Figure S. 20. *Hemiramphus brasiliensis*, underwater photo at Trindade Island, by H. T. Pinheiro.

Belonidae

Platybelone argalus (Lesueur 1821) §

WC; WA; NA; L (0-2); O (0-2); Seamounts-Islands: JAE (CIUFES 2133), TRI [18]; Figure S. 21.

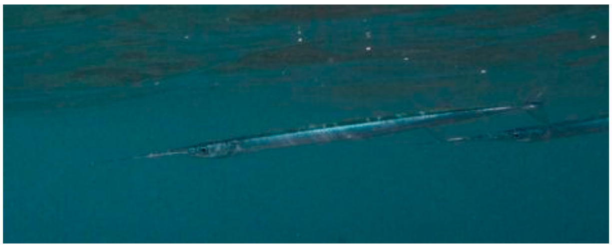

Figure S. 21. *Platybelone argalus* at Trindade Island, photo by H. T. Pinheiro.

Beryciformes

Holocentridae

Holocentrus adscensionis (Osbeck 1771) §

RS/RH; TA; NA; L (0-274); O (3-228); Seamounts-Islands: VIT (CIUFES 2035; ZUEC-PIS 8283), ALSAL (PHO), BESN (ZEE), ECL (PHO), JAS (VID), MONT (ZEE), JAE (PHO), DAV (PHO), DOG (VID), COL (ZEE), TRI (CIUFES 843, 1878, 2640, 131373, 131394; MZUSP 69010.0) [8] MAR [13]; Figure S. 22.

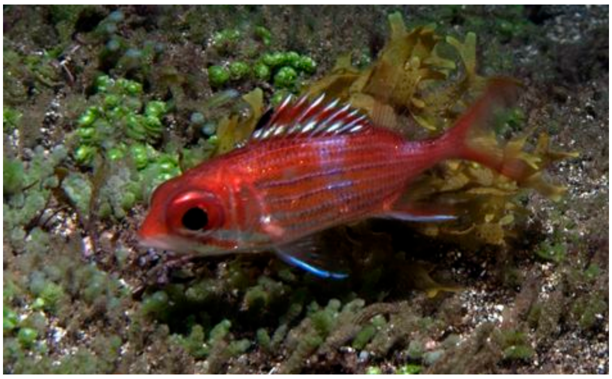

Figure S. 22. *Holocentrus adscensionis*, underwater photo at Trindade Island, by R. Francini-Filho.

Myripristis jacobus Cuvier 1829 §

RS/RH; TA; NA; L (0-100); O (2-71); Seamounts-Islands: VIT (PHO), ECL (VIS), JAE (PHO), DAV (PHO), TRI (ZUEC-PIS 2681) [18], MAR [20]; Figure S. 23.

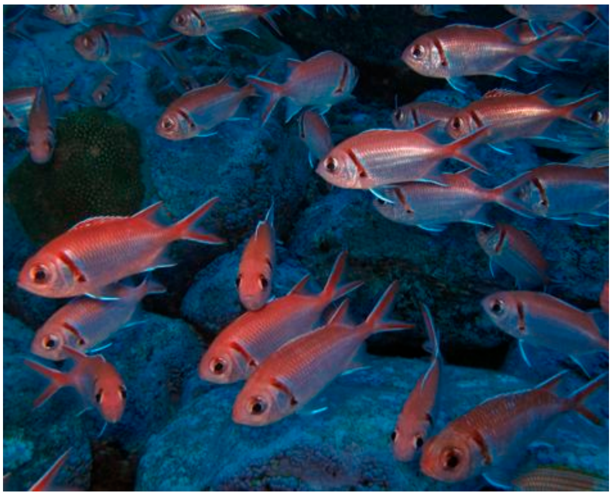

Figure S. 23. *Myripristis jacobus*, underwater photo at Trindade Island, by H. T. Pinheiro.

Plectrypops retrospinis (Guichenot 1853) §

RS; WA; NA; L (0-185); O (45); Seamounts-Islands: DAV (VIS), TRI (CIUFES 2380, 2834) [10].

Sargocentron bullisi (Woods 1955) §‡

RS; WA; NA; L (33-110); O (0-45); Seamounts-Islands: VIT (VIS), DAV (VIS), TRI (CIUFES 1600, 2381, 2398, 2431) [10], MAR [20].

Zeiformes

Zenionidae

Zenion hololepis (Goode & Bean 1896)

SD; CG; NA; L (180-650), O (52); Seamounts: VIT [19].

Gasterosteiformes

Syngnathidae

Hippocampus reidi Ginsburg 1933

RS/RH; WA; DD/OT; L (0-55), O (65); Seamounts: VIT [19].

Aulostomidae

Aulostomus strigosus Wheeler 1955 §‡

RS; TA; NA; L (5-25), O (10-45); Seamounts-Islands: VIT (PHO), TRI (CIUFES 2339) [20], MAR (CIUFES 0849) [10].

Fistulariidae

Fistularia petimba Lacepède 1803 §

RS, CT, NA; L (10-200); O (—); Island: TRI (PHO).

Scorpaeniformes

Dactylopteridae

Dactylopterus volitans (Linnaeus 1758) §

RH/SD; TA/M; NA; L (1-100); O (6-65); Seamounts-Islands: BESN (ZEE), DOG (VIS), TRI [10]; Figure S. 24.

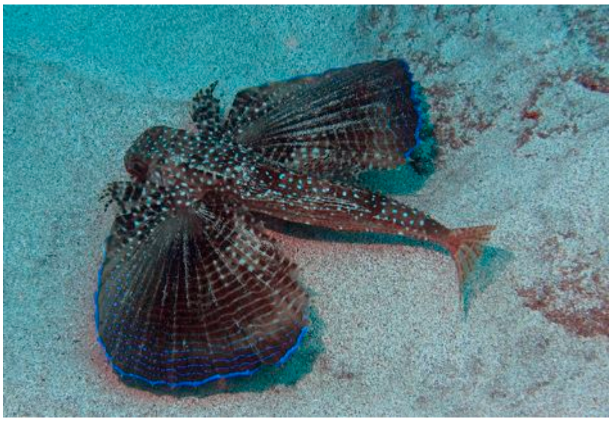

Figure S. 24. *Dactylopterus volitans*, underwater photo at Trindade Island, by H. T. Pinheiro.

Scorpaenidae

Pontinus longispinis Goode & Bean 1896 §

RS/RH; WA; NA; L (75-440); O (283-362); Seamounts-Islands: COL (ZEE), TRI (ZEE).

Pontinus rathbuni Goode & Bean 1896 §

RS/RH; WA; NA; L (0-366); O 52-315); Seamounts: VIT [19], DOG (ZEE).

Scorpaena brachyptera Eschmeyer 1965 §

RH; WA; NA; L (1-50); O (45); Seamounts-Islands: VIT (CIUFES 2185), TRI (CIUFES 2197, 2222, 2337, 2417) [20]; Figure S. 25.

Recorded as *Scorpaena albifimbria* Evermann & Marsh, 1900 in [20].

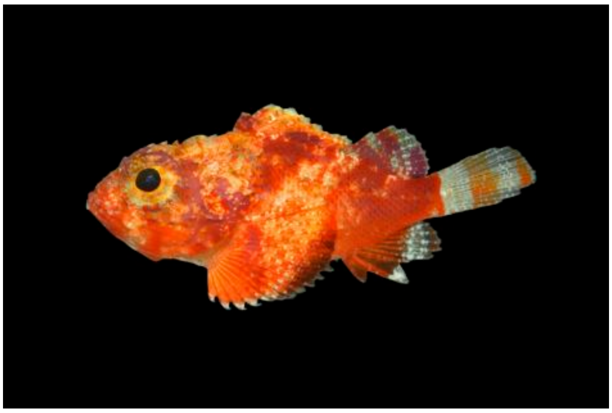

Figure S. 25. *Scorpaena brachyptera*, CIUFES 2185, 26.86mm TL, from Vitória Seamount, photo by R. M. Macieira.

Scorpaena brasiliensis Cuvier 1829

RS/RH; WA; NA; L (1-163); O (82); Seamounts: JAE [19].

Scorpaena dispar Longley & Hildebrand 1940

RS/RH; WA; NA; L (36-172); O (60-105); Seamounts: MONT [19], JAE [19], DOG [19], COL [19].

Scorpaena isthmensis Meek & Hildebrand 1928

RS/RH; WA; NA; L (8-100); O (20); Islands: TRI (CIUFES 2342, 2349) [20]; Figure S. 26.

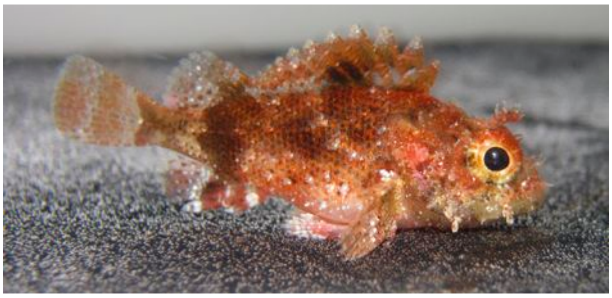

Figure S. 26. *Scorpaena isthmensis*, CIUFES 2342, 35mm TL, from Trindade Island, photo by T. Simon.

Scorpaena plumieri Bloch 1789

RS/RH; WA/CA; NA; L (1-60); O (0-35); Islands: TRI [18].

Scorpaenodes caribbaeus Meek & Hildebrand 1928

RS; WA; NA; L (0-35); O (15); Islands: TRI (CIUFES 2200, 2223) [10]; Figure S. 27.

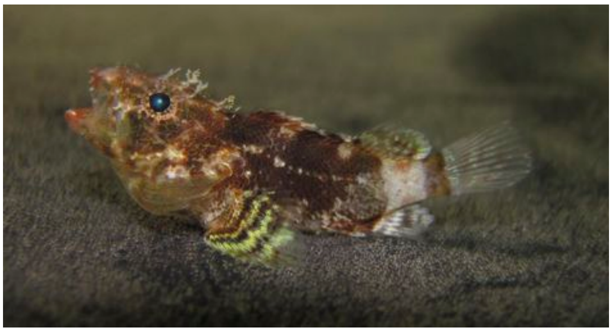

Figure S. 27. *Scorpaenodes caribbaeus*, CIUFES 2223, 23.5mm TL, from Trindade Island, photo by T. Simon.

Scorpaenodes tredecimspinosus (Metzelaar 1919) §

RS; WA; NA; L (82); O (65); Seamounts: DOG (CIUFES 2202); Figure S. 28.

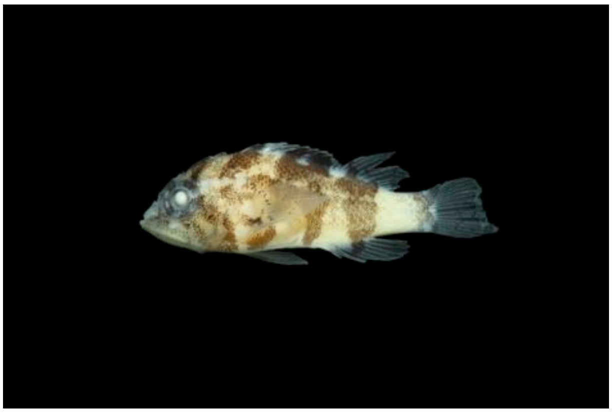

Figure S. 28. *Scorpaenodes tredecimspinosus*, CIUFES 2202, 13.65mm TL, from Dogaressa Seamount, photo by R. M. Macieira.

Perciformes

Acropomatidae

Synagrops sp. §

WC; O (42-49); Seamounts: DAV (ZEE).

Serranidae

Anthias menezesi Anderson & Heemstra 1980 §‡

RH; Br; NA; L (160-260), O (120); Seamounts: VIT (VID).

Pronotogrammus martinicensis (Guichenot 1868) §

RS/RH/WC; WA; NA; L (8-610); O (120); Seamounts: VIT (VID).

Serranus annularis (Günther 1880) §

RH; WA; NA; L (10-86); O (55-71); Seamounts-Islands: ECL (VIS), JAS (PHO), DOG (VID), JAE (CIUFES 2135), TRI (VIS); Figure S. 29.

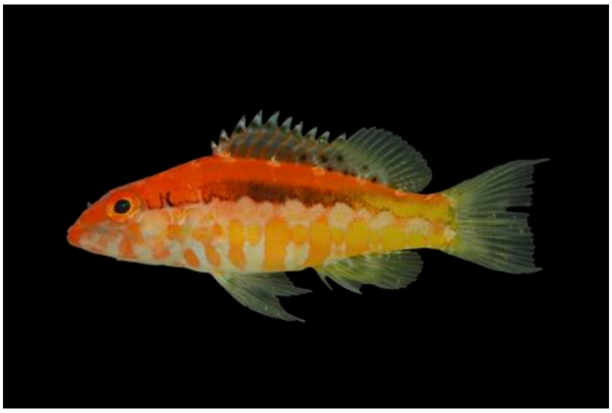

Figure S. 29. *Serranus annularis*, CIUFES 2135, 45.47mm TL, from Dogaressa seamount, photo by R. Macieira.

Serranus baldwini (Evermann & Marsch 1900) §

RS/RH; WA; NA; L (1-80); O (45-65); Seamounts: VIT (ZUEC-PIS 8291), JAS (VID), DOG (VIS).

Serranus phoebe Poey 1851 §

RH; WA; NA; L (27-400); O (82-120); Seamounts-Islands: VIT (VID), JAS (CIUFES 1887) [19], TRI (CIUFES 131401) [19], MAR [19].

Epinephelidae

Cephalopholis fulva (Linnaeus 1758) §

RS/RH; WA; LC; L (0-260), O (0-218); Seamounts-Islands: VIT (CIUFES 2061, 2125), ALSAL (PHO), BESN (ZEE), ECL (PHO), JAS (PHO), MONT (ZEE), JAE (PHO), DAV (ZUEC-PIS 8276); DOG (VID), COL (ZEE), TRI (CIUFES 1621; ZUEC-PIS 2700, 2822) [11], MAR [13]; Figure S. 30.

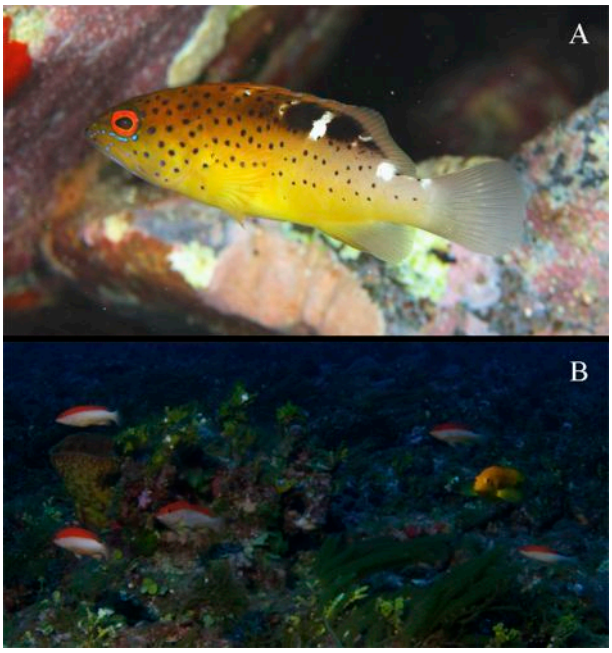

Figure S. 30. *Cephalopholis fulva*, underwater photos of different color morphs, by R. M. Macieira (A - Trindade Island) and R. L. Moura (B - Vitória seamount).

This grouper occurs at all surveyed sites, being common and abundant on islands reefs [23] and seamounts. Yellow morph adults were commonly found at VTC seamounts. Hybrids between *C. fulva* and *Paranthias furcifer* (described as *Menephorus dubius* by Poey 1860) (Figure S. 31) are recorded at Trindade [10] as well as at other Brazilian oceanic islands [24].

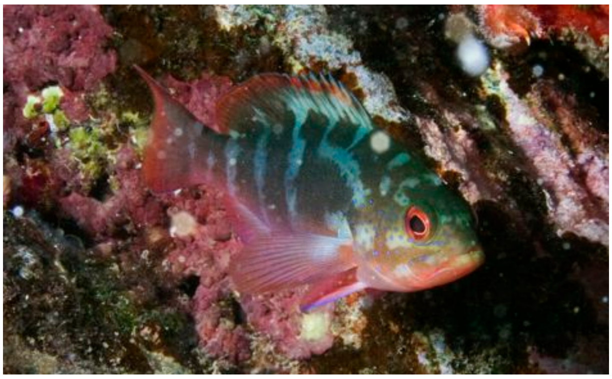

Figure S. 31. Hybrid between *Cephalopholis fulva* and *Paranthias furcifer*, underwater photo at Trindade Island, by R. Francini-Filho.

Dermatolepis inermis (Valenciennes 1833) §‡

RS/RH; WA; NT; L (3-213), O (3-315); Seamounts-Islands: VIT (ZEE), BESN (ZEE), ECL (ZEE), JAS (ZEE), JAE (VID), DAV (PHO), DOG (ZEE), COL (VID), TRI [18], MAR [20]; Figure S. 32.

The marbled grouper was the second-most abundant Epinephelidae recorded during dive surveys on seamounts. This abundance was unexpected, since the species is considered rare in the adjacent continental platform shelf.

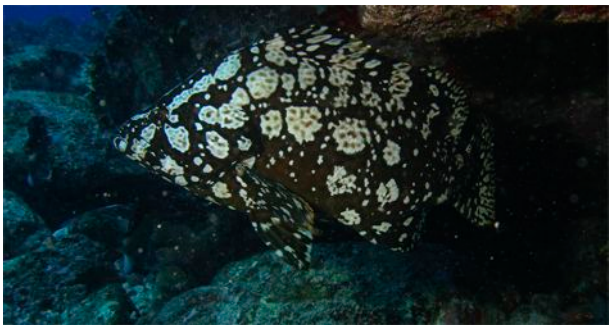

Figure S. 32. *Dermatolepis inermis*, underwater photo at Trindade Island, by T. Simon.

Epinephelus adscensionis (Osbeck 1765) ‡

RS; TA; LC; L (0-120), O (0-189); Islands: TRI (CIUFES 1851, 2639, 131387; ZUEC-PIS 2820) [21], MAR [13]; Figure S. 33.

This species is abundant in Brazilian northeastern continental shelf reefs and at Trindade [25], where it can reach up to 65cm TL.

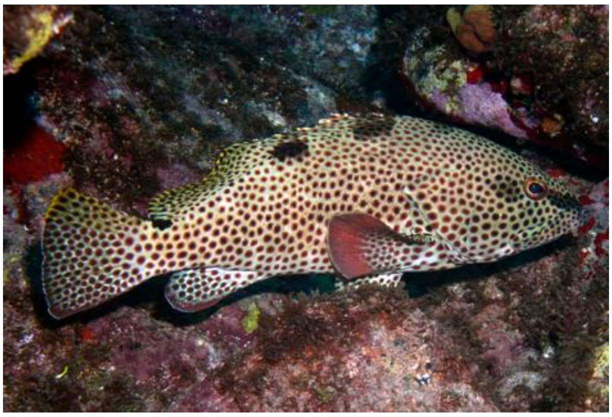

Figure S. 33. *Epinephelus adscensionis*, underwater photo at Trindade Island, by H. T. Pinheiro.

Epinephelus itajara (Lichtenstein 1822) §

RS; CG; CR/ET; L (0-100); O (55); Seamounts: JAE (PHO).

Epinephelus morio (Valenciennes 1828) §

RS/RH; WA; NT/OT; L: (5-330); O (—); Seamounts: ECL (ZEE).

Gonioplectrus hispanus (Cuvier 1828) §

RS; WA; LC; L (3-365); O (35-155); Seamounts-Islands: COL (ZEE), TRI (MBML-Peixes 274) [18].

Hyporthodus mystacinus (Poey 1852)

RS/RH; CG; LC; L (30-400); O (100-354); Seamounts-Islands: DOG [15], COL [15], TRI [15].

Hyporthodus nigritus (Holbrook 1855) §

RH; WA; CR; L (55-525); O (111); Seamounts: JAS (ZEE).

Hyporthodus niveatus (Valenciennes 1828)

RS; WA; VU/OT; L (30-525); O (90-260); Seamounts: BESN [15], ECL [15].

Liopropoma carmabi (Randall 1963) §

RS/RH; WA; NA; L (15-70); O (62); Seamounts: JAS (PHO).

Mycteroperca bonaci (Poey, 1860) §

RS; WA; NT/OT; L (1-140); O (5-55); Seamounts-Islands: VIT (VID), DAV (VIS), TRI [12].

Mycteroperca interstitialis (Poey 1860) §

RS; WA; VU; L (2-150); O (2-136); Seamounts-Islands: VIT (VID), JAS (FIS), DAV (PHO), DOG (ZEE), COL (ZEE), TRI [18].

Mycteroperca tigris (Valenciennes 1833) §

RS; WA; LC/ET; L (10-40); O (90-135); Seamounts: BESN (ZEE).

Mycteroperca venenosa (Linnaeus 1758) §‡

RS/RH; WA; NT; L (2-137), O (5-262); Seamounts-Islands: VIT (VID), JAS (ZEE), JAE (VID), DAV (PHO), DOG (FIS), COL (ZEE), TRI [12], MAR [20].

Paranthias furcifer (Valenciennes 1828) §

RS/RH/WC; TA; LC; L (8-100); O (5-71); Seamounts-Islands: VIT (PHO), ALSAL (PHO), ECL (PHO), JAS (PHO), JAE (CIUFES 1886), DAV (PHO), DOG (VID), TRI (CIUFES 2379) [10], MAR [20]; Figure S. 34.

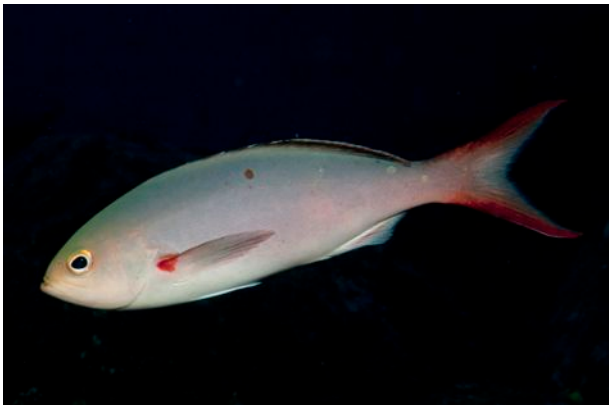

Figure S. 34. *Paranthias furcifer*, underwater photo at Trindade Island, by R. Francini-Filho.

Pseudogramma gregoryi (Breder 1927) §

RS/RH; WA; NA; L (1-260), O (45-85); Seamounts-Islands: VIT (CIUFES 2151, 2209, 2442, 2312; ZUEC-PIS 8290), JAE (CIUFES 2134, 2155), DOG (CIUFES 2154; ZUEC-PIS 8289), COL (CIUFES 2152; ZUEC-PIS 8288), TRI (CIUFES 2113, 2203) [20]; Figure S. 35.

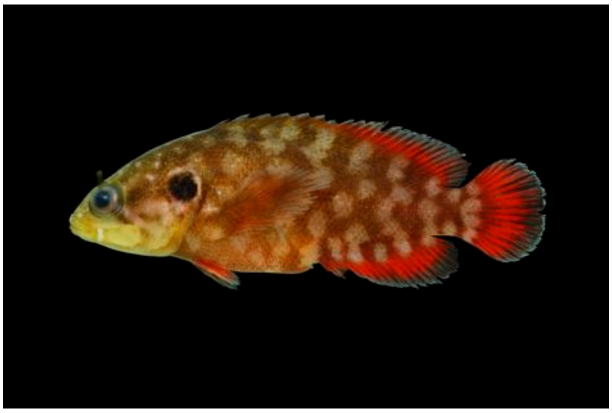

Figure S. 35. *Pseudogramma gregoryi*, CIUFES 2134, 37.24mm TL, from Columbia Bank, photo by R. M. Macieira.

Rypticus saponaceus (Schneider 1801) §

RS/RH; TA; NA; L (0-62); O (0-55); Seamounts-Islands: JAE (VID), DAV (PHO), TRI [11], MAR [20]; Figure S. 36.

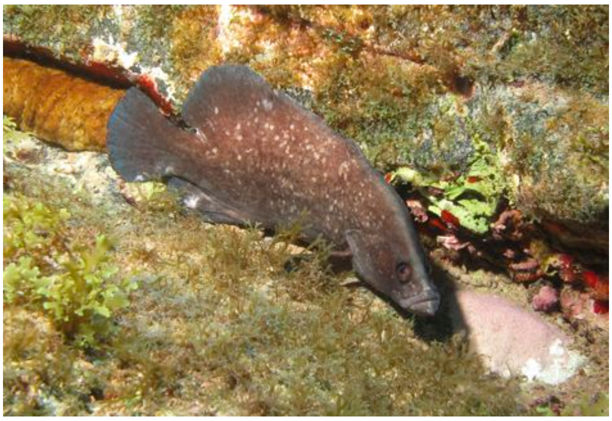

Figure S. 36. *Rypticus saponaceus*, underwater photo at Trindade Island, photo by T. Simon.

Opistognathidae

Opistognathus aff. *aurifrons* (Jordan & Thompson 1905) §‡

RH; Br; NA; L (59), O (15-65); Seamounts-Islands: JAE (CIUFES 2158), DAV (VIS), DOG (VID), COL (VIS), TRI (CIUFES 1548, 1549, 1584, 2306, 2334, 2441) [18]; Figure S. 37.

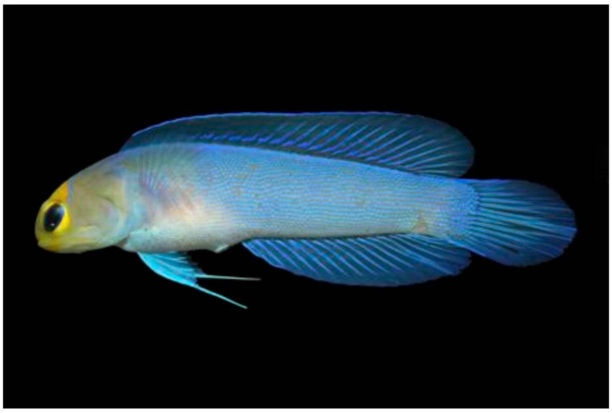

Figure S. 37. *Opistognathus* aff. *aurifrons*, CIUFES 1548, 71mm TL, from Trindade Island, photo by R. M. Macieira.

Opistognathus sp. §

RH; VTC; NA; L (—), O (10-65); Seamounts-Islands: DOG (CIUFES 2054), TRI (CIUFES 2345) [20]; Figure S. 38.

A new species under description found at VTC and at Fernando de Noronha, closely related to *Opistognathus maxillosus* Poey 1860 (W. F. Smith-Vaniz, unpublished data).

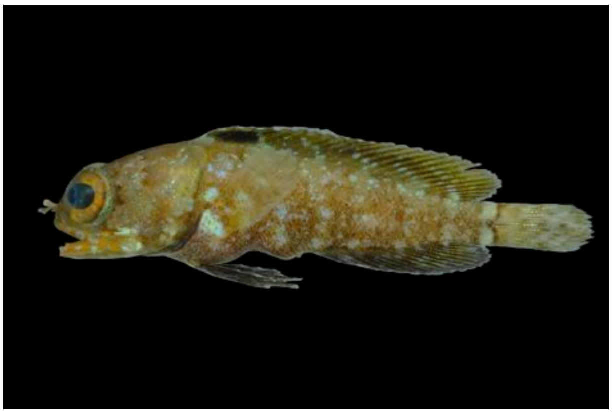

Figure S. 38. *Opistognathus* sp., CIUFES 2054, 35.34mm TL, from Dogaressa seamount, photo by R. M. Macieira.

Priacanthidae

Heteropriacanthus cruentatus (Lacépède 1801) §

RS; CG; NA; L (3-300); O (3-65); Seamounts-Islands: MONT (ZEE), JAE (VID), DAV (VIS), TRI (ZUEC-PIS 2839) [18], MAR [20]; Figure S. 39.

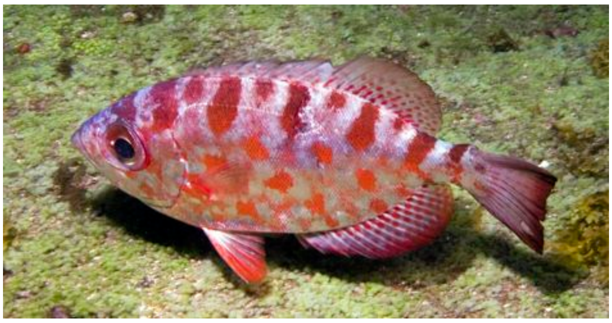

Figure S. 39. *Heteropriacanthus cruentatus*, underwater photo at Trindade Island, by R. Francini-Filho.

Priacanthus arenatus Cuvier, 1829 §

RS; TA/M; NA; L (10-200); O (20); Islands: MAR (VIS).

Apogonidae

Apogon americanus Castelnau 1855 §‡

RS; WA; NA; L (0-50), O (0-63); Seamounts-Islands: VIT (VIS), JAE (CIUFES 2053), DAV (CIUFES 2034; ZUEC-PIS 8273), TRI (CIUFES 1184, 1255, 1262, 1469, 1591, 1546, 2321, 2661) [18]; Figure S. 40.

This species is very common at VTC, but uncommon along the Brazilian coast.

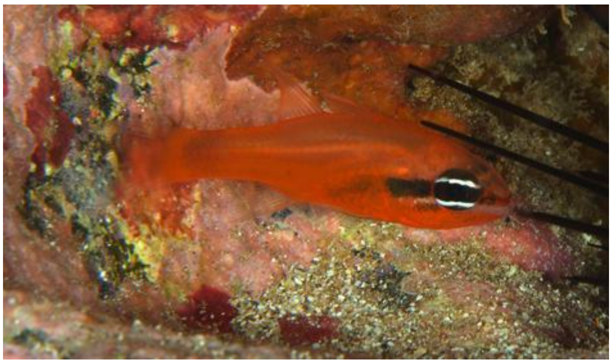

Figure S. 40. *Apogon americanus*, underwater photo at Trindade Island, by R. M. Macieira.

Apogon planifrons Longley & Hildebrand 1940 §

RS; WA; NA; L (3-30), O (5-10); Islands: TRI (PHO); Figure S. 41.

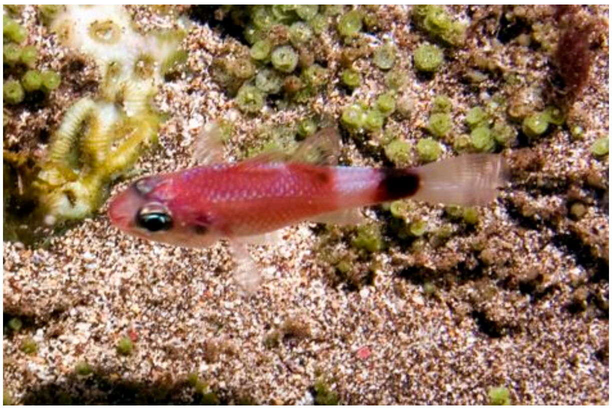

Figure S. 41. *Apogon planifrons*, underwater photo at Trindade Island, by R. Francini-Filho.

Apogon pseudomaculatus Longley 1932 §

RS; WA; NA; L (1-100); O (20-45); Seamounts: VIT [19], DAV (CIUFES 2044).

Astrapogon puncticulatus (Poey 1867)

RS/RH; WA; NA; L (0-15); O (0-10); Islands: TRI (CIUFES 1551) [10]; Figure S. 42.

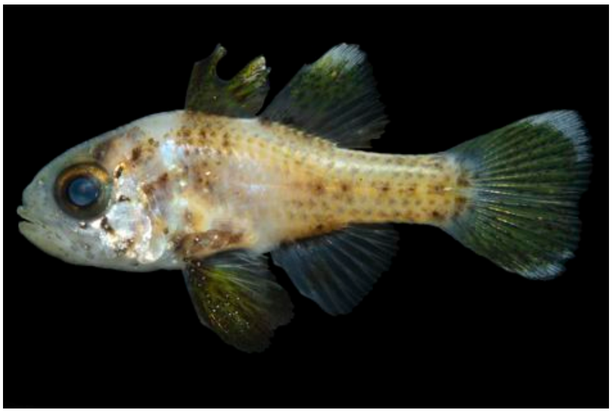

Figure S. 42. *Astrapogon puncticulatus*, CIUFES 1551, 26.00mm TL, from Trindade Island, photo by R. M. Macieira.

Phaeoptyx pigmentaria (Poey 1860) §‡

RS; TA; NA; L (13-50), O (2-55); Seamounts-Islands: VIT (CIUFES 2160; ZUEC-PIS 8285), JAE (CIUFES 2047), DAV (CIUFES 3979; ZUEC-PIS 8284),

TRI (CIUFES 844, 1556, 1594, 2418, 2660) [10]; Figure S. 43.

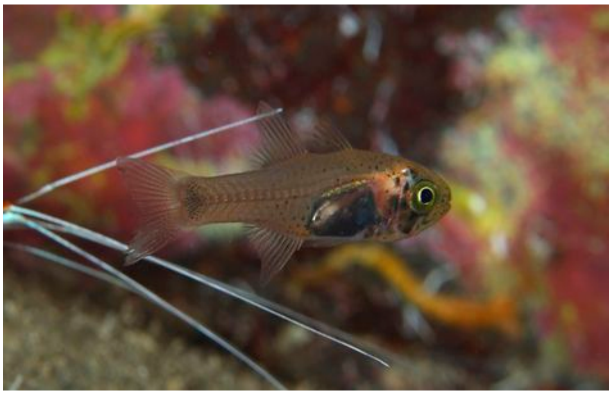

Figure S. 43. *Phaeoptyx pigmentaria*, underwater photo at Trindade Island, by R. M. Macieira.

Malacanthidae

Lopholatilus villarii Miranda Ribeiro 1915 §

RH; SW; NA; L (100-500), O (100-500); Seamounts: VIT (ZEE), BESN (ZEE), ECL (ZEE).

Malacanthus plumieri (Bloch 1786) §

RH; WA; NA; L (2-153); O (5-131); Seamounts-Islands: VIT (VID), ALSAL (PHO), ECL (PHO), JAS (FIS), MONT (ZEE), JAE (CIUFES 1888), DAV (VID), DOG (ZEE), COL (VID), TRI (CIUFES 1539) [11], MAR [20]; Figure S. 44.

The sand tilefish is common on VTC rhodolith beds, building nests composed of piles of rhodoliths and others material (*e.g.*, bivalve shells). This tilefish is an ecosystem engineer species, as its nests increase the structural complexity of the rhodolith beds, sheltering many associate species. This process seems to promote an increase in the fish biodiversity at the rhodolith beds.

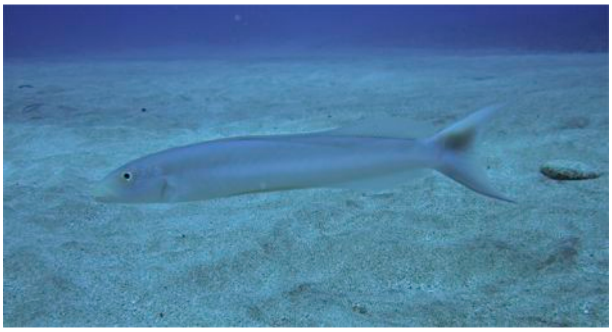

Figure S. 44. *Malacanthus plumieri*, underwater photo at Trindade Island, by R. M. Macieira.

Coryphaenidae

Coryphaena hippurus Linnaeus 1758 §‡

WC; CT; LC; L (5-85), O (5-91); Seamounts-Islands: VIT [14], ALSAL [14], BESN [14], MONT (FIS), TRI [8].

Echeneidae

Echeneis naucrates Linnaeus 1758

WC; CT; NA; L (0-50); O (1-35); Islands: TRI [11].

Remora remora (Linnaeus, 1758) §

WC; CG; NA; L (0-100); O (10); Seamounts-Islands: VIT (PHO); TRI [10].

Carangidae

Alectis ciliaris (Bloch 1787) §

WC; CT; LC; L (60-100); O (3-10); Seamounts-Islands: VIT (ZEE), COL (ZEE), TRI [10].

Carangoides bartholomaei (Cuvier 1833) §

RS/RH/WC; TA; NA; L (0-70); O (3-55); Seamounts-Islands: JAE (VID), TRI [12].

Caranx crysos (Mitchill 1815) §

RS/RH/WC; TA; LC; L (0-130); O (3-84); Seamounts-Islands: VIT (PHO), BESN (ZEE), ECL (PHO), JAS (VID), MONT (ZEE), JAE (VIS), DAV (VID), DOG (FIS), COL (ZEE), TRI [10], MAR [10]; Figure S. 45.

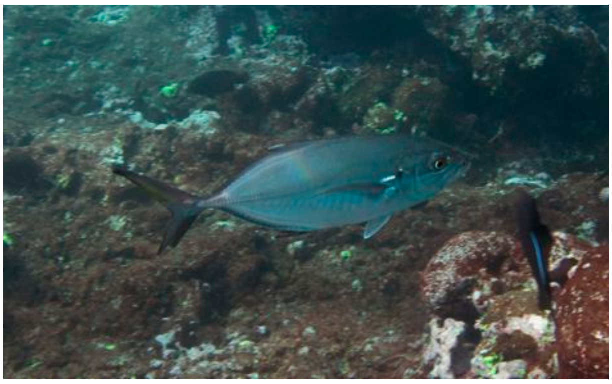

Figure S. 45. *Caranx crysos*, underwater photo at Trindade Island, by H. T. Pinheiro.

Caranx latus Agassiz 1831 §

RS/RH/WC; TA; NA; L (0-140); O (0-125); Seamounts-Islands: VIT (PHO), JAS (ZEE), JAE (PHO), DAV (PHO), DOG (PHO), COL (ZEE), TRI (ZUEC-PIS 2762, 2763) [18].

Caranx lugubris Poey 1860 §‡

RS/RH/WC; CT; NA; L (12-365); O (0-85); Seamounts-Islands: VIT (VID), JAS (FIS), DAV (PHO), DOG (PHO), COL (VID), TRI (VID), TRI (ZUEC-PIS 2829) [8], MAR [13]; Figure S. 46.

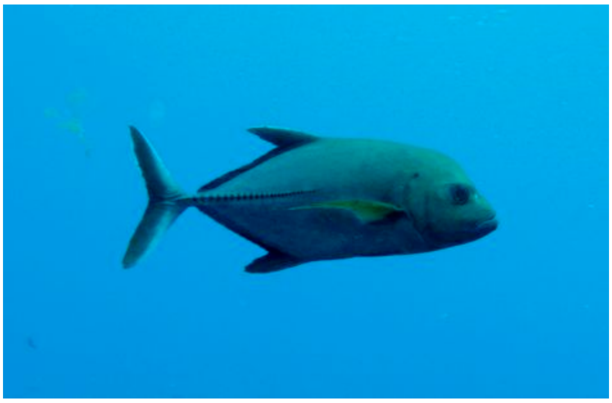

Figure S. 46. *Caranx lugubris*, underwater photo at Trindade Island, by H. T. Pinheiro.

Caranx ruber (Bloch 1793) §‡

RS/RH/WC; CT; NA; L (1-70), O (3-106); Seamounts-Islands: VIT (PHO), JAS (ZEE), MONT (ZEE), JAE (PHO), DAV (PHO), DOG (ZEE), COL (ZEE), TRI (ZUEC-PIS 2821) [18], MAR [20].

Decapterus macarellus (Cuvier 1833) §

WC; CG; NA; L (0-400); O (2-65); Seamounts-Islands: VIT (VIS), DAV (VIS), DOG (VIS), TRI [12]; Figure S. 47.

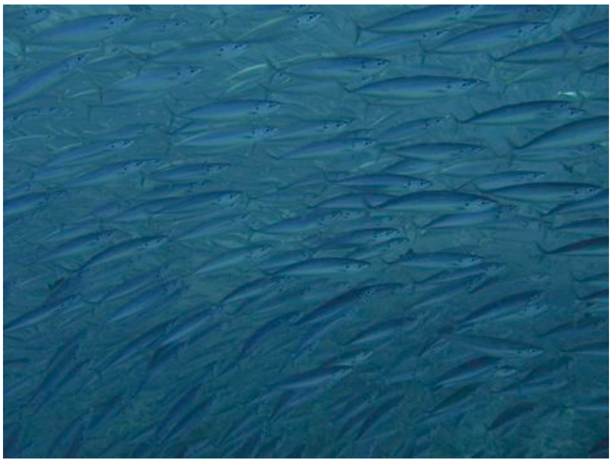

Figure S. 47. *Decapterus macarellus*, underwater photo at Trindade Island, by H. T. Pinheiro.

Decapterus tabl Berry 1968 §

WC; CG; NA; L (7-400); O (29-68); Seamounts: BESN (ZEE), JAS (ZEE), MONT (ZEE), DAV (ZEE).

Elagatis bipinnulata (Quoy & Gaimard 1825) §

WC; CG; NA; L (0-150); O (2-59); Seamounts-Islands: VIT (PHO), JAE (VID), DAV (VID), DOG (VIS), TRI [18].

Selar crumenophthalmus (Bloch 1793)

WC; CT; NA; L (0-170); O (2-10); Islands: TRI (CIUFES 842, 1486) [12], MAR [20].

Seriola dumerili (Risso 1810) §

RS/RH/WC; CG; NA; L (1-360); O (59-124); Seamounts-Islands: VIT (PHO), ECL (ZEE), JAS (ZEE), DAV (ZEE), DOG (ZEE), COL (ZEE), TRI (ZEE).

Seriola fasciata (Bloch 1793) §‡

RS/RH/WC; WA; NA; L (55-150), O (49-94); Seamounts: ECL (ZEE), DAV (ZEE).

Seriola lalandi Valenciennes 1833 §

RS/RH/WC; CG; NA; L (3-825); O (59); Seamounts: VIT (ZEE), COL (ZEE).

Seriola rivoliana Valenciennes 1833 §

RS/RH/WC; CG; NA; L (5-320); O (5-118); Seamounts-Islands: VIT (VID), ALSAL (PHO), JAS (PHO), MONT (FIS), DAV (VID), DOG (VIS), COL (ZEE), TRI [18], MAR [20]; Figure S. 48.

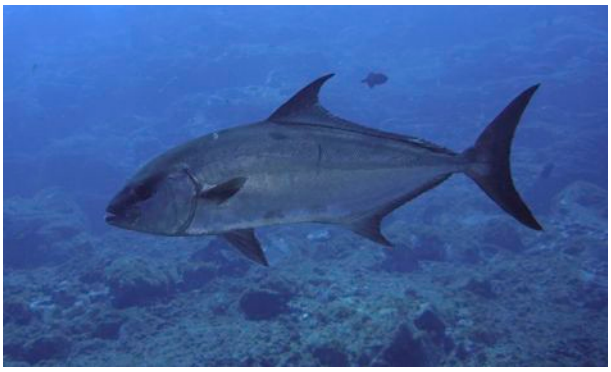

Figure S. 48. *Seriola rivoliana*, underwater photo at Trindade Island, by T. Simon.

Seriola zonata (Mitchill 1815) §

WC; WA; NA; L (—); O (35); Seamounts: BESN (ZEE).

Uraspis secunda (Poey 1860)

RS/WC; CG; NA; L (1-36); O (2-15); Islands: TRI [18].

Emmelichthyidae

Erythrocles monodi Poll & Cadenat 1954 §‡

WC; TA; NA; L (90-300), O (45); Seamounts: DAV (ZEE).

Lutjanidae

Etelis oculatus (Valenciennes 1828)

RS/RH/WC; WA; NA; L (100-450); O (122-287); Seamounts-Islands: BESN [15], DAV [15], COL [15], TRI [15].

Lutjanus analis (Cuvier 1828) §

RS/RH/SD; WA; VU/OT; L (25-95); O (58-100); Seamounts: JAS (ZEE), DAV (ZEE).

Lutjanus jocu (Bloch & Schneider 1801) §

RS; TA; NA; L (2-64); O (55); Seamounts-Islands: JAE (VID), TRI (PHO).

Lutjanus vivanus (Cuvier 1828) §

RS/RH; WA; NA; Seamounts-Islands: VIT (ZEE), BESN (ZEE), ECL (ZEE), JAS (ZEE), DAV (PHO), DOG (ZEE), TRI [18].

Ocyurus chrysurus (Bloch 1791) §

RS/WC; WA; OT; L (0-188); O (50-59); Seamounts: VIT (ZEE), DAV (VID), COL (ZEE).

Rhomboplites aurorubens (Cuvier 1829) §‡

RS/RH/WC; WA; OT; L (40-300), O (20-112); Seamounts: BESN (ZEE), ECL (ZEE), JAS (FIS).

Haemulidae

Despite their high abundance and richness in reefs along the continental shelf and in other Brazilian oceanic islands (Rocas Atoll and Fernando de Noronha Archipelago), only one haemulid was found on the VTC, where it is only known from Trindade Island reefs.

Anisotremus surinamensis (Bloch 1791)

RS; WA; NA; L (0-70); O (0-44); Islands: TRI (ZUEC-PIS 2994) [8]; Figure S. 49.

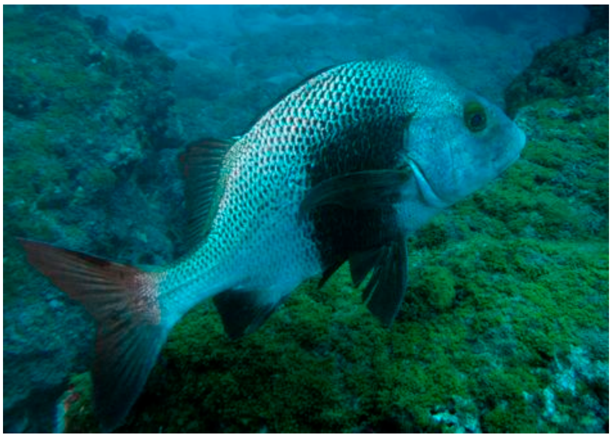

Figure S. 49. *Anisotremus surinamensis*, underwater photo at Trindade Island, by H. T. Pinheiro.

Sparidae

Diplodus argenteus (Valenciennes 1830)

RS; WA; NA; L (0-28); O (0-15); Islands: TRI (ZUEC-PIS 2821) [11], MAR [13]; Figure S. 50.

Absent from the seamounts of the VTC, probably due to ecological requirements (rocky reefs exposed to waves).

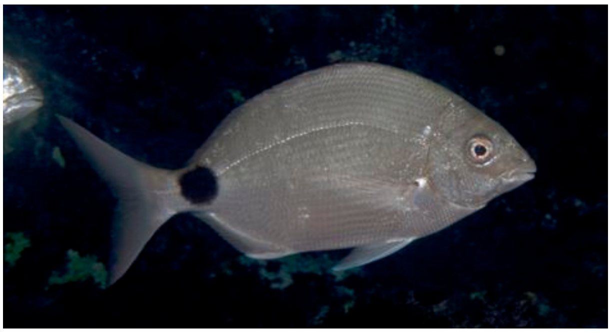

Figure S. 50. *Diplodus argenteus*, underwater photo at Trindade Island, by R. Francini-Filho.

Pagrus pagrus (Linnaeus 1758) §

RH; TA/M; ED/OT; L (0-250); O (59-63); Seamounts: VIT (ZEE), COL (ZEE).

Sciaenidae

Equetus lanceolatus (Linnaeus 1758) §

RS; WA; NA; L (10-230); O (45-55); Seamounts: VIT [19], JAE (CIUFES 2060), DAV (VID).

This species is common at VTC, but uncommon along the Brazilian coast.

Pareques acuminatus (Bloch & Schneider 1801) §

RS; WA; NA; L (0-110); O (45); Seamounts: VIT (PHO), JAE (VID).

Mullidae

Mulloidichthys martinicus (Cuvier 1829) §

RS/RH/SD; TA; NA; L (0-135); O (0-55); Seamounts-Islands: VIT (PHO), JAE (VID), DAV (PHO), TRI (ZUEC-PIS 2812) [18], MAR [20].

Pseudupeneus maculatus (Bloch 1793) §

RS/RH; WA; NA; L (0-90); O (0-65); Seamounts-Islands: VIT [19], JAE (VID), DAV (PHO), TRI [18], MAR [20]; Figure S. 51.

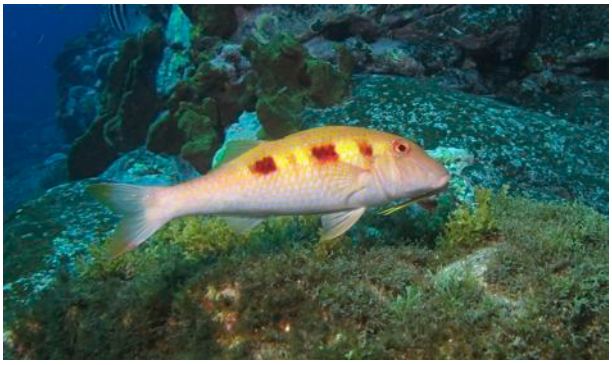

Figure S. 51. *Pseudopeneus maculatus*, underwater photo at Trindade Island, by H. T. Pinheiro.

Pempheridae

Pempheris poeyi Bean 1885

RS; WC; NA; L (—); O (0-1); Islands: TRI (CIUFES 2423, 2432) [11], Figure S. 52.

Trindade Island is the only known locality of occurrence of *P. poeyi* within the Brazilian Province, characterizing a large disjunctive distribution between VTC and the Caribbean [10].

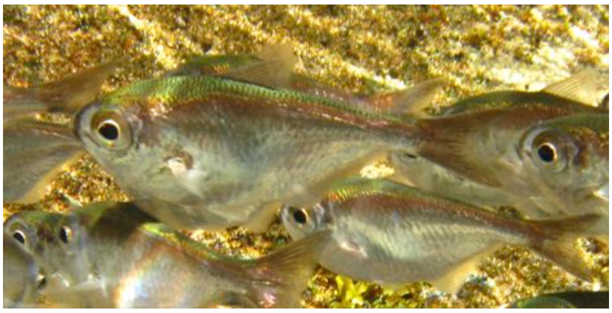

Figure S. 52. *Pempheris poeyi*, underwater photo at Trindade Island, by J. L. Gasparini.

Pempheris schomburgki Müller & Troschel 1848

RS; WA; NA; L (3-30); O (3-6); Islands: TRI (CIUFES 850) [10], Figure S. 53.

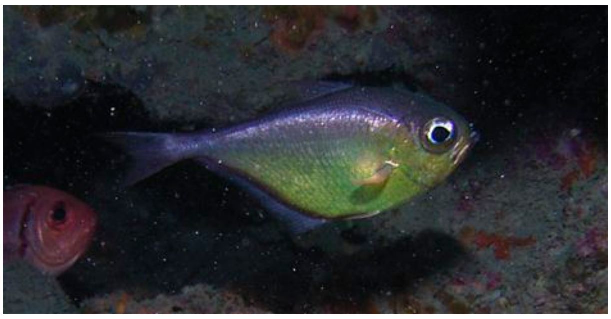

Figure S. 53. *Pempheris schomburgki*, underwater photo at Trindade Island, by T. Simon.

Kyphosidae

Kyphosus sectatrix (Linnaeus 1758), following [26] §‡

RS; TA; NA; L (1-40 m), O (1-55 m); Seamounts-Islands: VIT (VID), JAE (PHO), DAV (PHO), TRI (CIUFES 1620; ZUEC-PIS 2830) [11], MAR [13].

The individuals collected at Trindade Island (CIUFES 1620; ZUEC-PIS 2830) match *K. sectatrix* following [26], but match *K. bosquii* following [27]. We adopt here the name *K. sectatrix* for our specimens. Video and photo analyses indicate that other two species may occur at Trindade Island, *Kyphosus cinerascens* (Forsskål 1775) and *Kyphosus vaigiensis* (Quoy & Gaimard 1825) [26]. Actual sampling of individuals of this genus in the VTC is needed for an accurate identification of these two supposedly additional species.

Chaetodontidae

Chaetodon sedentarius Poey 1860 §

RS/RH; WA; LC; L (5-100); O (45-58); Seamounts-Islands: VIT (PHO), DAV (PHO), DOG (VID), TRI (VID).

Chaetodon striatus Linnaeus 1758 §‡

RS/RH; WA/CA; LC; L (2-60), O (2-65); Seamounts-Islands: VIT (VID), JAE (VID), DAV (CIUFES 2127), DOG (VID), TRI (ZUEC-PIS 2683) [11], MAR [20].

Prognathodes brasiliensis Burgess 2001 §‡

RS; Br; LC; L (50), O (5-65); Seamounts-Islands: VIT [19], ALSAL (VID), JAS (PHO), JAE (CIUFES 2031), DAV (ZUEC-PIS 8287), DOG (CIUFES 2056), TRI (CIUFES 1550) [18], MAR [13]; Figure S. 54.

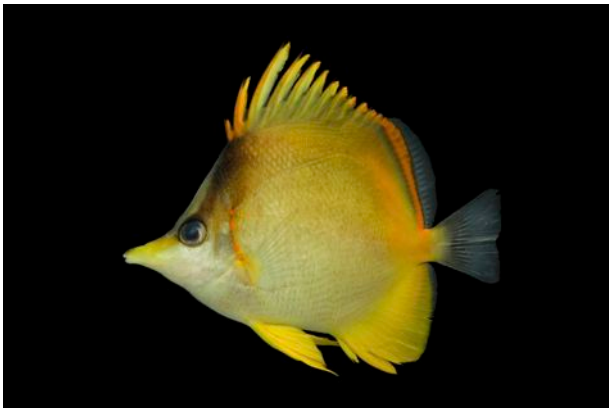

Figure S. 54. *Prognathodes brasiliensis*, CIUFES 1550, 90.00mm TL, from Trindade Island, photo by R. M. Macieira.

Prognathodes guyanensis (Durand 1960)

RS/RH; WA; LC; L (60-250); O (65); Seamounts: VIT [19].

Pomacanthidae

Centropyge aurantonotus Burgess 1974 §

RS/RH; WA; LC; L (2-300); O (15-85); Seamounts-Islands: VIT (PHO), ECL (PHO), JAS (PHO), MONT [19], JAE (VIS), DAV (CIUFES 2041), DOG (CIUFES 2037; ZUEC-PIS 8263) [19], COL (CIUFES 2036, 2194; ZUEC-PIS 8275), TRI (CIUFES 2208) [18], MAR (VIS); Figure S. 55.

This species is very common at VTC, but is uncommon in Brazilian coast. Its spatial distribution is associated with rubble mounds of *M. plumieri*.

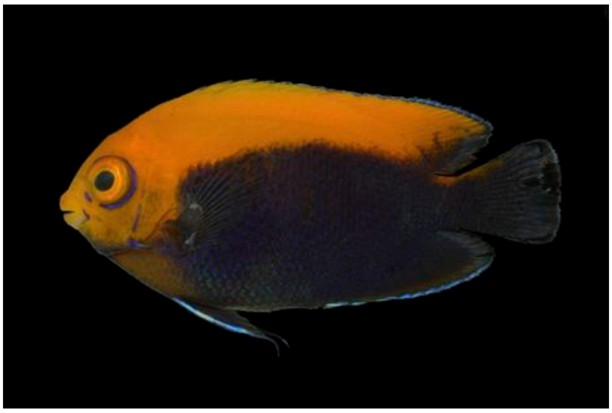

Figure S. 55. *Centropyge aurantonotus*, CIUFES 2037, 45.25mm TL, from Dogaressa seamount, photo by R. M. Macieira.

Holacanthus ciliaris (Linnaeus 1758) §‡

RS/RH; WA/CA; LC; L (1-70), O (45-120); Seamounts: VIT (PHO), ECL (VIS), JAE (VID), DAV (VID).

This species is found along the Brazilian shelf and oceanic islands, except for Trindade and Martin Vaz.

Holacanthus tricolor (Bloch 1795) §

RS/RH; WA; LC; L (3-135); O (3-71); Seamounts-Islands: VIT (CIUFES 2128), ALSAL (PHO), ECL (VIS), JAS (PHO), JAE (PHO), DAV (PHO), DOG (CIUFES 2046), TRI (CIUFES 1576, 2307, 2338, 2340; ZUEC-PIS 2835) [18], MAR [20]; Figure S. 56.

This species is absent from all other Brazilian oceanic islands, and Joyeux *et al.* [28] suggested that this scenario is a case of inter-specific exclusion with *Pomacanthus paru* (Bloch, 1787). The gray angelfish *Pomacanthus arcuatus* (Linnaeus, 1758) and the French angelfish *P. paru* are commonly found along the Brazilian continental shelf and oceanic islands, but both are absent from the VTC [18].

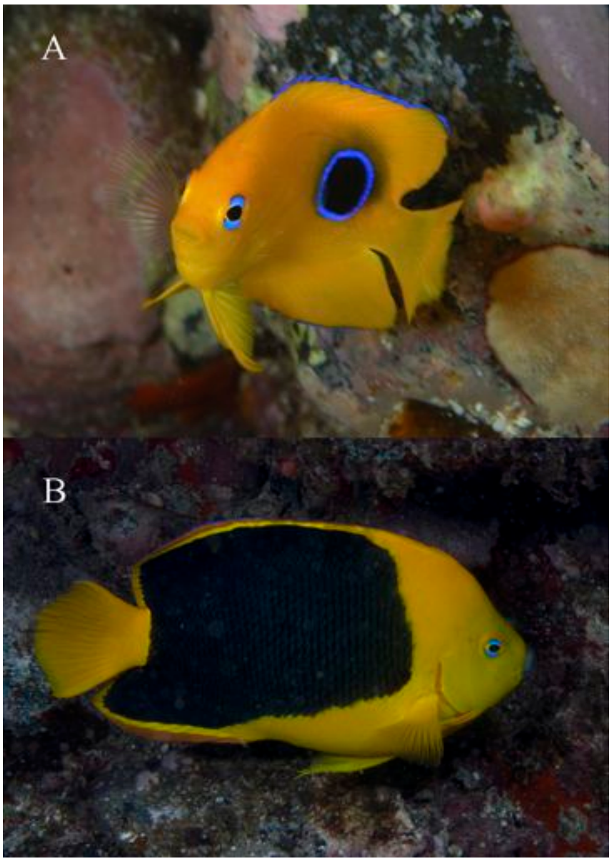

Figure S. 56. *Holacanthus tricolor*, underwater photos at Trindade Island. (A) Juvenile by R. M. Macieira and (B) adult phase by R. Francini-Filho.

Cirrhitidae

Amblycirrhitus pinos (Mowbray 1927) §‡

RS/RH; WA; NA; L (2-46), O (12-66); Seamounts-Islands: VIT (PHO), ALSAL (VID), JAE (VIS), DAV (PHO), DOG (ZUEC-PIS 8262), TRI (CIUFES 2210) [10].

Pomacentridae

Abudefduf saxatilis (Linnaeus 1758)

RS; TA; NA; L (0-41); O (3-31); Islands: TRI (CIUFES 1254, 1583, 1638, 1856, 2707, 131372, 131397) [21], MAR [13]; Figure S. 57.

Absent from the VTC, probably due to ecological requirements of the juvenile phase (shallow-water dwellers).

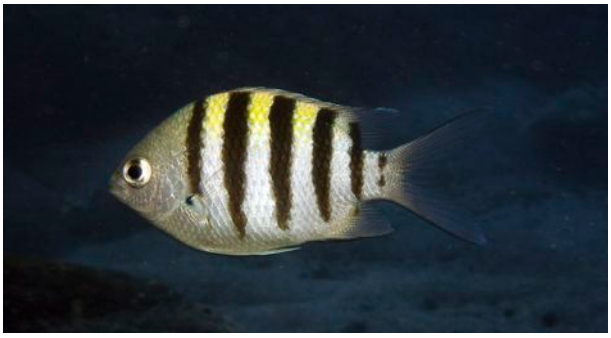

Figure S. 57. *Abudefduf saxatilis*, underwater photo at Trindade Island, by J.-C. Joyeux.

Chromis aff. *enchrysura* Jordan & Gilbert 1882 §

RS/RH; TA; NA; L (4-124); O (50-120); Seamounts: VIT (PHO), ECL (VIS).

Chromis flavicauda (Günther 1880) §‡

RS/RH; WA; DD; L (50-60), O (50-120); Seamounts-Islands: VIT (PHO), JAS (VID), JAE (VID), DAV (VID), DOG (ZUEC-PIS 8264), COL (CIUFES 2043), TRI (CIUFES 2207, 2212) [20], MAR [19]; Figure S. 58.

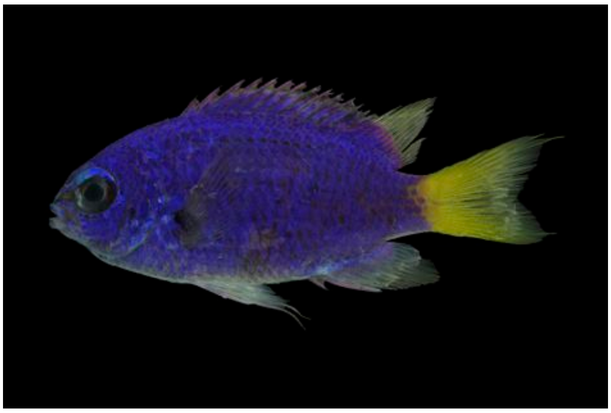

Figure S. 58. *Chromis flavicauda*, CIUFES 2045, 50.45mm TL, from Dogaressa seamount, photo by R. M. Macieira.

Chromis jubauna Moura 1995 §‡

RS/RH; Br; NA; L (10-54), O (40-71); Seamounts-Islands: VIT (CIUFES 2126), ECL (VIS), JAE (CIUFES 2039; ZUEC-PIS 8277), DAV (VID), TRI [13].

Chromis multilineata (Guichenot 1853) §‡

RS/WC; TA; NA; L (0-60), O (3-84); Seamounts-Islands: VIT (PHO), JAE (VID), DAV (VIS), TRI (CIUFES 1464, 1587, 131388) [18], MAR [13].

Microspathodon chrysurus (Cuvier 1830) §

RS/RH; WA; NA; L (0-120); O (3-62); Seamounts-Islands: JAE (VIS), DAV (CIUFES 2124), TRI (CIUFES 1258, 1466, 1498; ZUEC-PIS 2696) [18], MAR [13]; Figure S. 59.

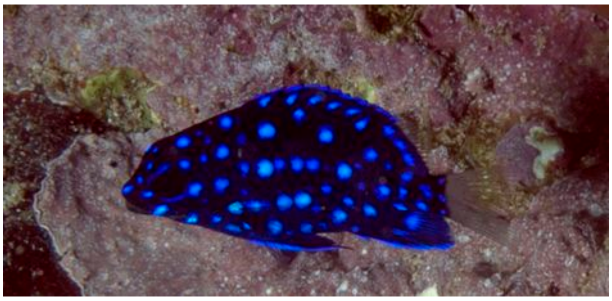

Figure S. 59. *Microspathodon chrysurus*, underwater photo at Trindade Island, by R. Francini-Filho.

Stegastes fuscus trindadensis (Cuvier 1830) §‡

RS; Br; LC; L (0-12), O (0-55); Seamounts-Islands: JAE (CIUFES 2124), DAV (CIUFES 2033), TRI (CIUFES 345, 576, 853, 1404, 1540, 2211, 2703, 131390; ZUEC-PIS 2688, 2689, 3031, 33155, 3156 - five paratypes of *Stegastes trindadensis*, plus ZUEC-PIS 2650, 2690, 2691, 3185, 3186, 3030) [11], MAR [13]; Figure S. 60.

The population found at Trindade Island was described as a distinct species, *Stegastes trindadensis* (Gasparini, Moura & Sazima 1999), but is currently considered a subspecies of *S. fuscus*.

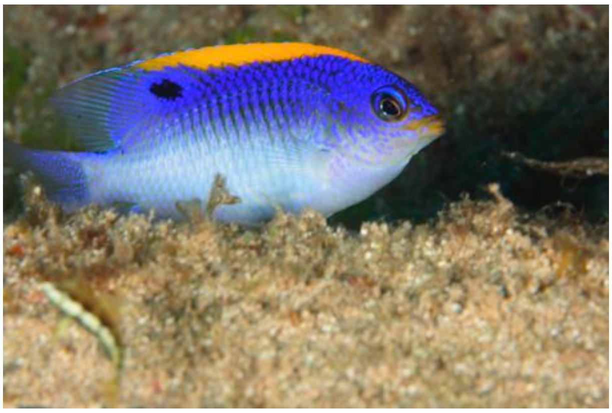

Figure S. 60. *Stegastes fuscus trindadensis*, underwater photo at Trindade Island, by R. M. Macieira.

Stegastes pictus (Castelnau 1855) §‡

RS/RH; WA; NA; L (6-70), O (5-85); Seamounts-Islands: VIT (CIUFES 2040), ALSAL (PHO), ECL (PHO), JAS (PHO), JAE (CIUFES 2048), DAV (ZUEC-PIS 8272), DOG (VID), COL (CIUFES 2042), TRI (CIUFES 857, 1580, 2206, 2242) [10], MAR [13]; Figure S. 61.

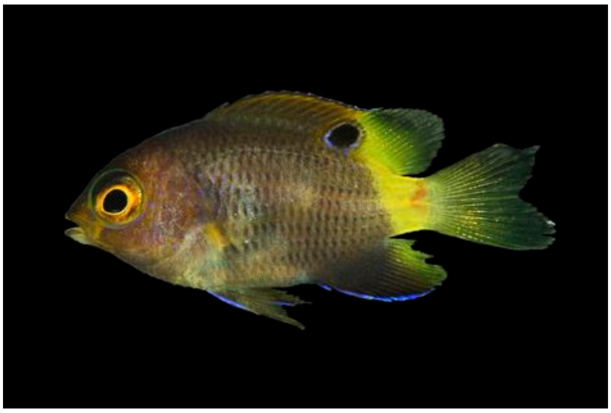

Figure S. 61. *Stegastes pictus*, CIUFES 2146, 35mm TL, from at Davis seamount, photo by R. M. Macieira.

Labridae

Thirteen labrid species had their depth range extended at the VTC.

Bodianus pulchellus (Poey 1860) §

RS/RH; TA; LC; L (1-120); O (10-84); Seamounts-Islands: VIT (PHO), JAE (PHO), DAV (PHO), TRI [18], MAR [20].

Bodianus rufus (Linnaeus 1758) §

RS; WA; LC; L (1-70); O (5-45); Seamounts-Islands: VIT (VIS), DAV (VIS), TRI [12], MAR [20].

Clepticus brasiliensis Heiser, Moura & Robertson 2001 §‡

RS/WC; Br; LC; L (5-54), O (6-62); Seamounts-Islands: VIT (PHO), JAS (VIS), DAV (PHO), TRI [10]; Figure S. 62.

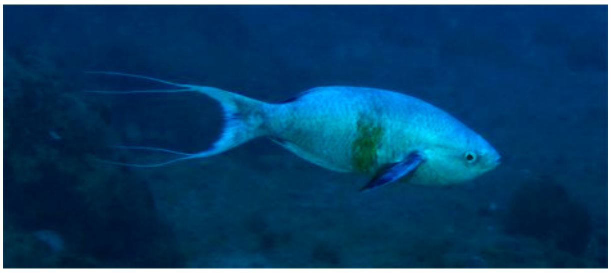

Figure S. 62. *Clepticus brasiliensis*, underwater photo at Trindade Island, by H. T. Pinheiro.

Cryptotomus roseus Cope 1871 §‡

RS/RH; WA; LC; L (0-60), O (12-66); Seamounts-Islands: VIT (PHO), ALSAL (VID), DAV (VID), TRI (CIUFES 1494) [10]; Figure S. 63.

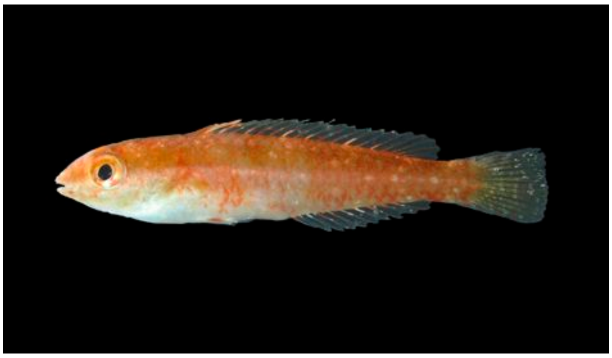

Figure S. 63. *Cryptotomus roseus*, CIUFES 3131, 35.5mm TL, from Trindade Island, photo by H. T. Pinheiro.

Decodon puellaris (Poey 1860) §

RS; WA; DD; L (18-275); O (—); Seamounts: DAV (PHO).

Doratonotus megalepis Günther 1862 ‡

RS/RH; TA; LC; L (0-15); O (16); Island: TRI (CIUFES 1541) [20].

Halichoeres brasiliensis (Bloch 1791) ‡

RS, Br, DD, L (0-35), O (0-44); Islands: TRI (CIUFES 1542, 1569, 2825, 1398) [21], MAR [13]; Figure S. 64.

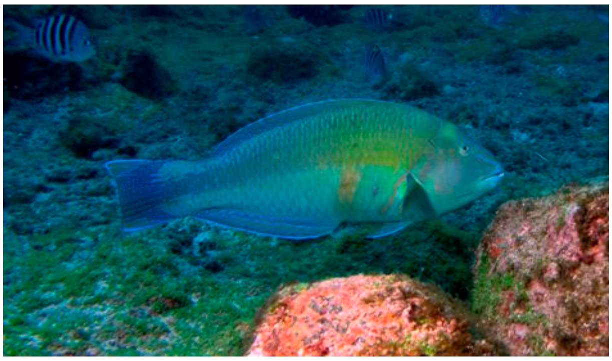

Figure S. 64. *Halichoeres brasiliensis*, underwater photo at Trindade Island, by H. T. Pinheiro.

Halichoeres dimidiatus (Agassiz 1831) §‡

RS/RH; Br; LC; L (3-70); O (45-71); Seamounts: VIT (PHO), ALSAL (VIS), ECL (PHO), JAS (VIS), JAE (VID), DAV (PHO), DOG (CIUFES 2189).

Halichoeres penrosei (Starks 1913) §‡

RS/RH; Br; LC; L (0-40), O (0-66); Seamounts-Islands: ALSAL (VIS), DAV (VIS), DOG (VID), TRI (CIUFES 566, 839, 1259, 1286, 1543, 2700, 2826, 131366, 131386; ZUEC-PIS 3135) [18], MAR [20].

Halichoeres poeyi (Steindachner 1867) §‡

RS/RH; WA; LC; L (1-54), O (0-71); Seamounts-Islands: ECL (VIS), JAE (VIS), TRI (CIUFES 840, 1261, 1396) [18], MAR [13]; Figure S. 65.

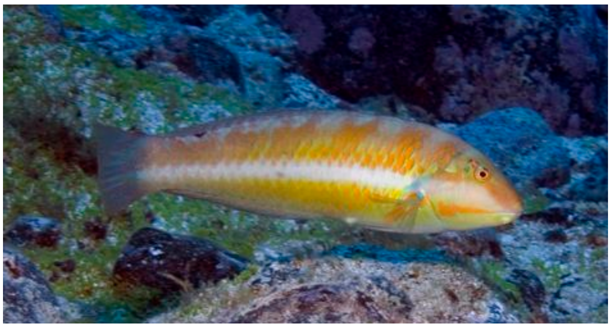

Figure S. 65. *Halichoeres poeyi*, underwater photo at Martin Vaz, by R. Francini-Filho.

Halichoeres rubrovirens Rocha, Pinheiro & Gasparini 2010 §‡

RS/RH; VTC; NA; L (25), O (5-84); Seamounts-Islands: VIT (CIUFES 2137), ALSAL (VID), JAS (VID), JAE (PHO), DAV (PHO), DOG (ZUEC-PIS 8282), TRI (CIUFES 1279, 1440, 1470, 1471, 1472, 1473, 1474, 1475, 2284, 2711, 2710, 2819; ZUEC-PIS 3299) [10], MAR [10]; Figure S. 66.

The Brazilian-endemic *H. brasiliensis,* as well as *H. poeyi* are very abundant and common on the continental shelf and at VTC islands, but are absent from its seamounts. On the other hand, *H. dimidiatus*, despite recorded on seven VTC seamounts, is not found at the islands. *H. rubrovirens,* previously believed to be endemic from Trindade and Martin Vaz [29], actually is endemic to all VTC volcanic buildings, including the southern-most and isolated Almirante Saldanha. This species is considered as a relict and its closest relative is known from the eastern Pacific [29].

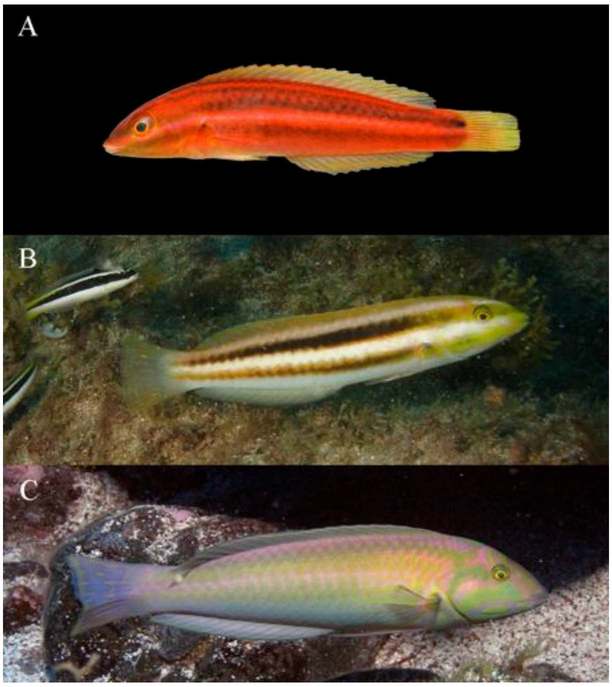

Figure S. 66. (A) *Halichoeres rubrovirens*, CIUFES 2131, 65.09mm TL, from Vitória seamount, photo by R. M. Macieira, (B) underwater photo at Trindade Island, by T. Simon and (C) underwater photo at Martin Vaz, by R. Francini-Filho.

Scarus zelindae Moura, Figueiredo & Sazima 2001 §‡

RS/RH; Br; DD; L (1-54); O (30-55); Seamounts: DAV (PHO).

The first record of this genus at a Brazilian oceanic locality.

Sparisoma amplum (Ranzani 1841) §‡

RS/RH; Br; LC; L (1-54), O (3-57); Seamounts-Islands: VIT (PHO), DAV (PHO), TRI (CIUFES1585; ZUEC-PIS 2858) [18], MAR [20]; Figure S. 67.

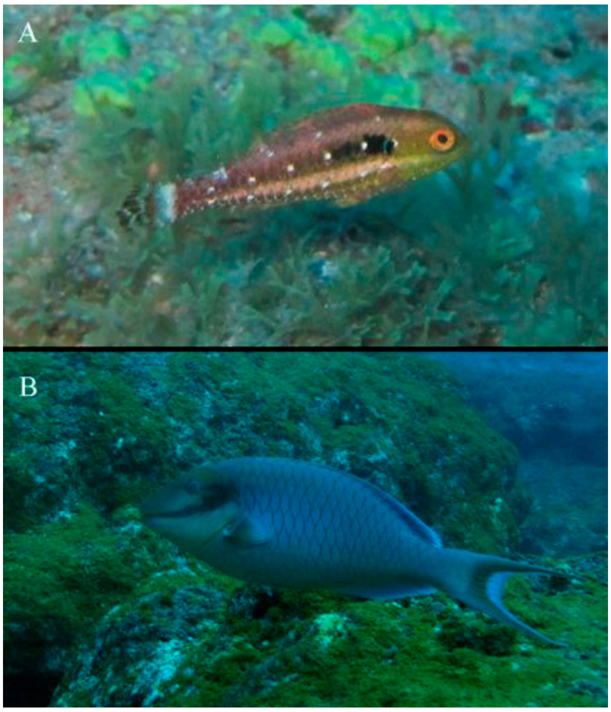

Figure S. 67. *Sparisoma amplum*, underwater photo at Trindade Island, by H. T. Pinheiro. (A) Juvenile and (B) Terminal phase.

Sparisoma axillare (Steindachner 1878) ‡

RS; Br; DD; L (1-35), O (2-45); Islands: TRI (CIUFES 2425, 2831) [18], MAR [20].

Sparisoma frondosum (Agassiz 1831) §

RS; Br; DD; L (5-45); O (20-45); Seamounts: VIT (VIS), DAV (VIS).

Sparisoma radians (Valenciennes 1840) §

RS/RH; WA; LC; L (1-77); O (62-71); Seamounts: VIT (VID), ECL (VIS).

Sparisoma rocha Pinheiro, Gasparini & Sazima 2010 §‡

RS/RH; VTC; NA; L (10-25), O (5-85); Seamounts-Islands: DAV (PHO), DOG (VIS), COL (CIUFES 2142; ZUEC-PIS 8292), TRI (CIUFES 0848 – holotype, 1529 – paratype; ZUEC-PIS 6349, 6350 – two paratypes) [12]; Figure S. 68.

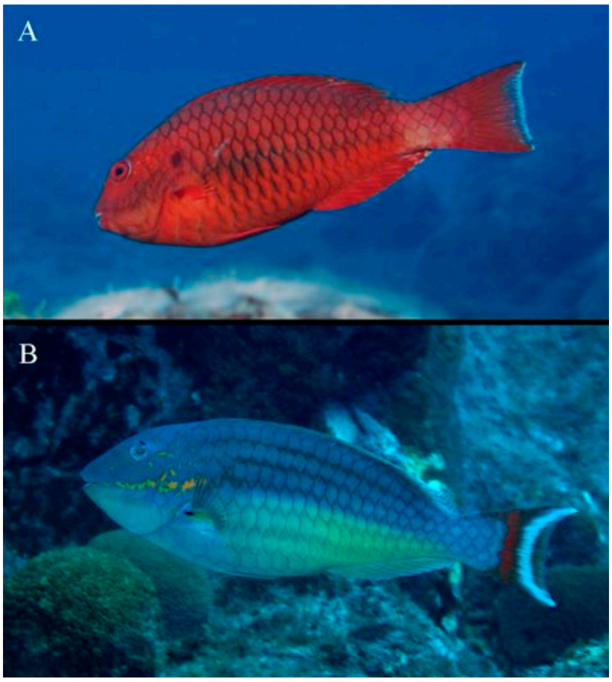

Figure S. 68. *Sparisoma rocha*, underwater photo at Trindade Island, by H. T. Pinheiro. (A) Female, intermediary phase, and (B) terminal phase.

Sparisoma tuiupiranga Gasparini, Joyeux & Floeter 2003§‡

RS/RH; Br; LC; L (5-20), O (45-71); Seamounts-Islands: VIT (VID), ALSAL (VID), ECL (VIS), JAS (PHO), DAV (PHO), DOG (ZUEC-PIS 8271), TRI [18].

Thalassoma noronhanum (Boulenger 1890) §‡

RS/RH; Br; LC; L (0-70), O (0-85); Seamounts-Islands: VIT (PHO), ALSAL (VID), JAS (ZEE), JAE (PHO), DAV (CIUFES 2055), DOG (VID), COL (VID), TRI (CIUFES 2659; ZUEC-PIS 2651, 2653, 2654, 2684, 3131, 3132, 3133, 3298) [18], MAR [13]; Figure S. 69.

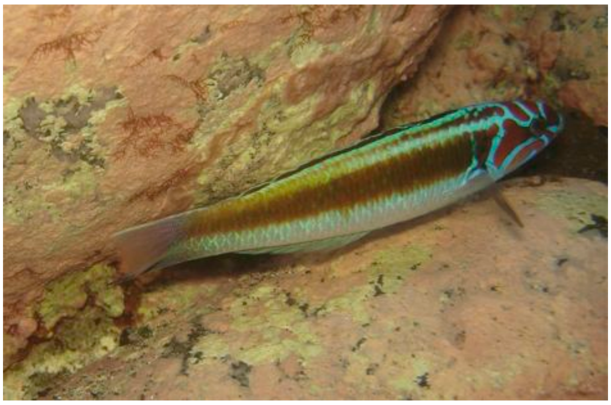

Figure S. 69. *Thalassoma noronhanum* underwater photo at Trindade Island, by J. C. Joyeux.

Xyrichtys novacula (Linnaeus 1758)

RH/SD; TA/M; LC; L (1-110); O (15-48); Seamount-Islands: JAS [19], TRI (CIUFES 3007) [12], MAR [19]; Figure S. 70.

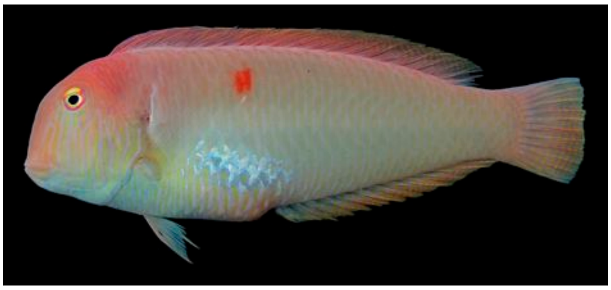

Figure S. 70. *Xyrichthys novacula*, CIUFES 3007, 155.5mm TL, from Trindade Island, photo by H.T. Pinheiro.

Xyrichtys splendens Castelnau 1855

RH/SD; WA; LC; L (3-90); O (10-20); Islands: TRI (CIUFES 1008, 3006) [10]; Figure S. 71.

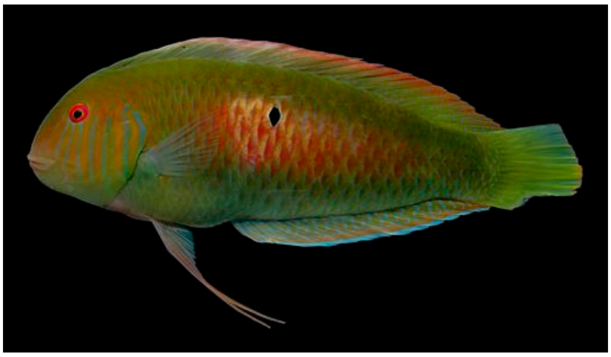

Figure S. 71. *Xyrichthys splendens*, CIUFES 3006, 160mm TL, from Trindade Island, photo by H. T. Pinheiro.

Xyrichtys sp. §

RH; NA; O (65-84); Seamounts: DOG (VID), COL (CIUFES 2186).

Apparently a new species of razorfish found on the VTC seamounts.

Tripterygiidae

Enneanectes altivelis Rosenblatt 1960 §‡

RS; WA; NA; L (3-25), O (3-45); Seamounts-Islands: DAV (CIUFES 2141, 2149; ZUEC-PIS 8267), TRI (CIUFES 1561, 1592, 2315, 2236, 2303, 2430, 2453, 2912) [18]; Figure S. 72.

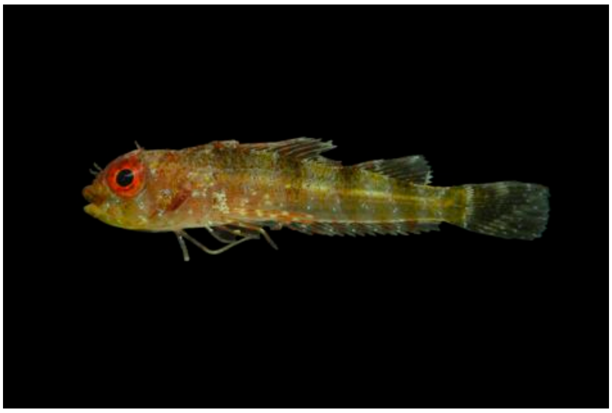

Figure S. 72. *Enneanectes altivelis*, CIUFES 2141, 17.02mm TL, from Davis seamount, photo by R. M. Macieira.

Blenniidae

Entomacrodus sp.

RS; TE; NA; L (—); O (0-6); Islands: TRI (CIUFES 1181, 1265, 1384, 1391, 1392, 1395, 1597 2427, 2926, 131376, 131421; ZUEC-PIS 2655, 6219) [11], MAR [20]; Figure S. 73.

An undescribed species.

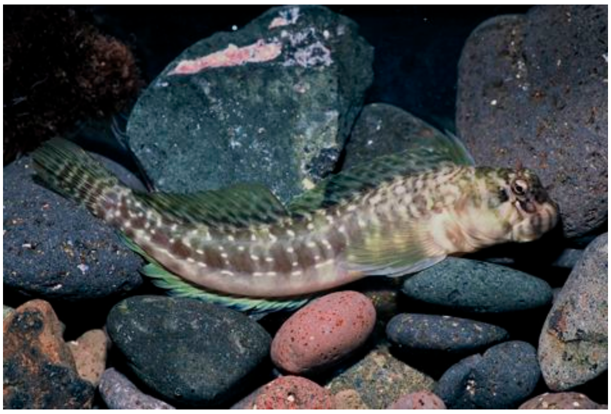

Figure S. 73. *Entomacrodus* sp., from Trindade Island, photo by J. L. Gasparini.

Hypleurochilus brasil Pinheiro, Gasparini & Rangel 2013 §‡

RS; VTC; NA; L (3-15); O (4-45); Seamounts-Islands: DAV (ZUEC-PIS 8269), TRI (CIUFES 1925, 1938, 1942, 1944, 1945, 1946, 1555, 1901, 1918, 1924, 2215; ZUEC-PIS 6353) [18], MAR [13]; Figure S. 74.

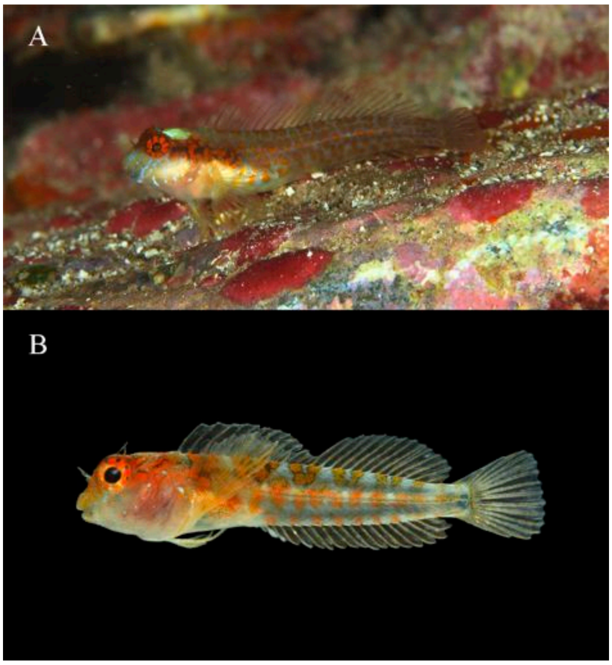

Figure S. 74. (A) *Hypleurochilus brasil*, underwater photo at Trindade Island, and (B) CIUFES 2136, 17.78mm TL, from Davis seamount, photos by R. M. Macieira.

Ophioblennius trinitatis Miranda-Ribeiro 1919 §

RS; Br; NA; L (0-53); O (0-20); Seamounts-Islands: DAV (VIS), TRI (CIUFES 570, 1578, 2662, 2708, 131377; ZUEC-PIS 2657, 2685, 2686) [11], MAR [13]; Figure S. 75.

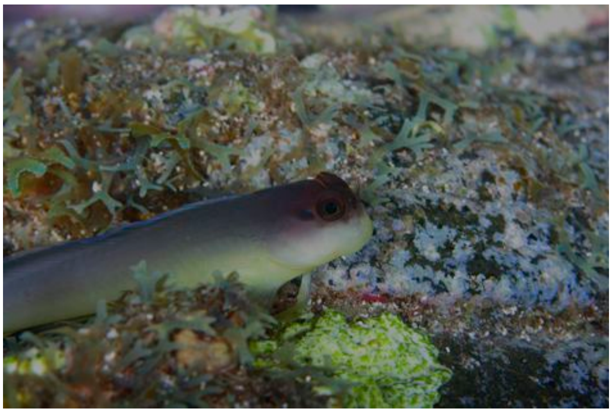

Figure S. 75. *Ophioblennius trinitatis*, underwater photo at Trindade Island, photo by R. M. Macieira.

Scartella poiti Rangel, Gasparini & Guimarães 2004

RS; TE; NA; L (0-1); O (0-1); Islands: TRI (CIUFES 1257, 2305, 2308, 2310, 2318, 2343, 2350, 2696, 2932, 131422; ZUEC-PIS 5413 - paratype, 6220) [11], MAR [13]; Figure S. 76.

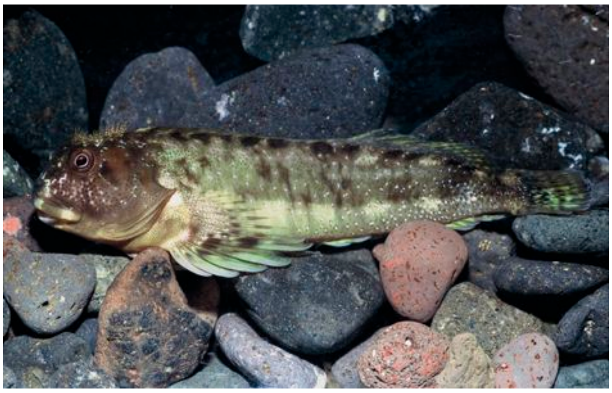

Figure S. 76. *Scartella poiti*, from Trindade Island, photo by J. L. Gasparini.

Labrisomidae

Labrisomus nuchipinnis (Quoy & Gaimard 1824)

RS; TA; NA; L (0-21); O (0-5); Islands: TRI (CIUFES 1253, 1263, 1285, 1465, 1537, 2638, 2697, 131369; ZUEC-PIS 2649, 2682) [21], MAR [13]; Figure S. 77.

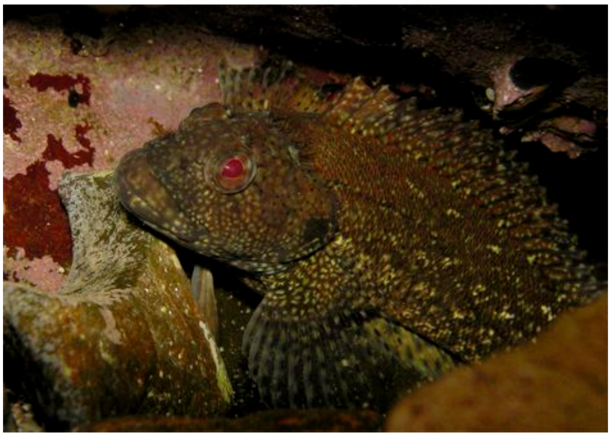

Figure S. 77. *Labrisomus nuchipinnis*, underwater photo at Trindade Island, photo by R. M. Macieira.

Malacoctenus brunoi Guimarães, Nunan & Gasparini 2010 ‡

RS; TE; NA; L (0-8), O (0-10); Islands: TRI (CIUFES 1316, 1317, 2666, 2695; ZUEC-PIS 2652) [18]; MAR [13]; Figure S. 78.

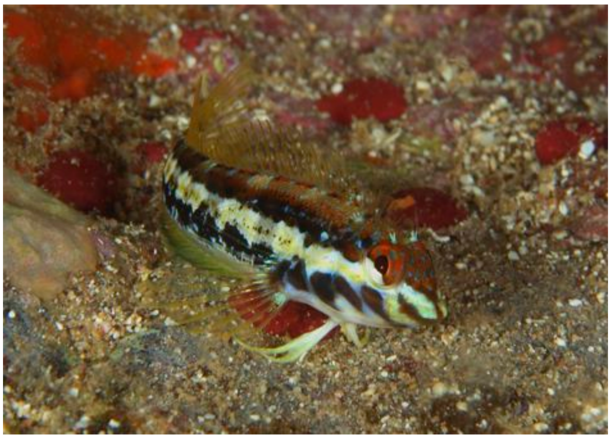

Figure S. 78. *Malacoctenus brunoi*, underwater photo at Trindade Island, photo by R. M. Macieira.

Chaenopsidae

Emblemariopsis signifer (Ginsburg 1942) §

RS; WA; LC; L (2-55); O (3-55); Seamounts-Islands: VIT (CIUFES 2188; ZUEC-PIS 8279), DAV (CIUFES 2195; ZUEC-PIS 8266), TRI (CIUFES 1565, 2199, 2221, 2294, 2301, 2311, 2316, 2319, 2331, 2353, 2422, 2445, 2450, 2455, 2913) [20]; Figure S. 79.

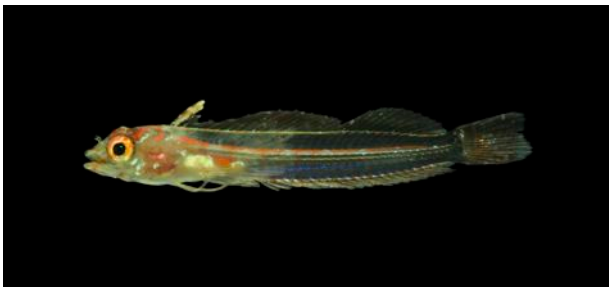

Figure S. 79. *Emblemariopsis signifer*, CIUFES 2143, 12.50mm TL, from Davis seamount, photo by R. M. Macieira.

Gobiesocidae

Acyrtus sp. ‡

RS; TE; NA; L (0-1); O (0-15); Islands: TRI (CIUFES 1566, 1570, 2292, 2299, 2309, 2323, 2439, 2665; ZUEC-PIS 2764) [18]; Figure S. 80.

An undescribed species, misidentified as *Arcos* sp. in Gasparini & Floeter [18] and Simon *et al.* [20].

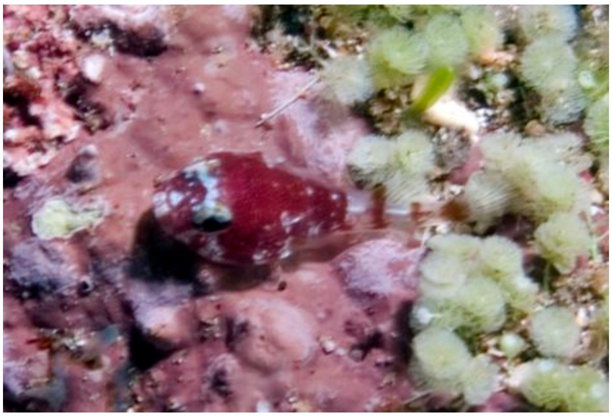

Figure S. 80. *Acyrtus* sp., underwater photo at Trindade Island, by R. Francini-Filho.

Tomicodon sp.

RS; TE; LC; L (0-1); O (0-1); Islands: TRI (CIUFES 1492, 1571, 2288, 2428, 2440, 2698, 2907, 2908, 2911) [10].

An undescribed species.

Callionymidae

Callionymus bairdi Jordan 1888

RH/SD; WA; NA; L (1-90); O (7-18); Islands: TRI (CIUFES 1495, 1560, 1588, 2328) [10]; Figure S. 81.

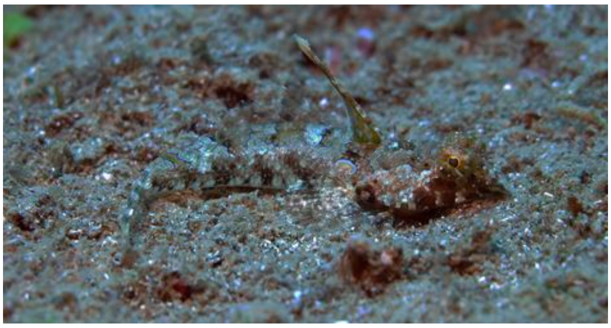

Figure S. 81. *Callionymus bairdi*, underwater photo at Trindade Island, by T. Simon.

Gobiidae

Chriolepis fisheri Herre 1942 ‡

RH/SD; WA; NA; L (82), O (15); Islands: TRI (CIUFES 1568, 1586, 2324) [20]; Figure S. 82.

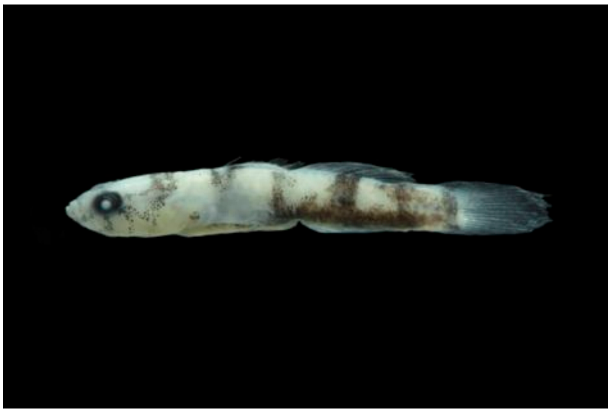

Figure S. 82. *Chriolepis fisheri*, CIUFES 2324, 11.15mm TL, from Trindade Island, photo by R. M. Macieira.

Coryphopterus thrix Böhlke & Robins 1960 §‡

RH/SD; WA; NA; L (10-54), O (6-85); Seamounts-Islands: VIT (CIUFES 2138, 2140; ZUEC-PIS 8278) [19], ALSAL (VIS), ECL (VIS), JAS [19], JAE (CIUFES 2192), DAV (CIUFES 2139; ZUEC-PIS 8265) [19], DOG [19], COL (CIUFES 2187), TRI (CIUFES 1496, 1574, 2217, 2228 2235, 2420, 2443, 2448, 2664) [19], MAR [20]; Figure S. 83.

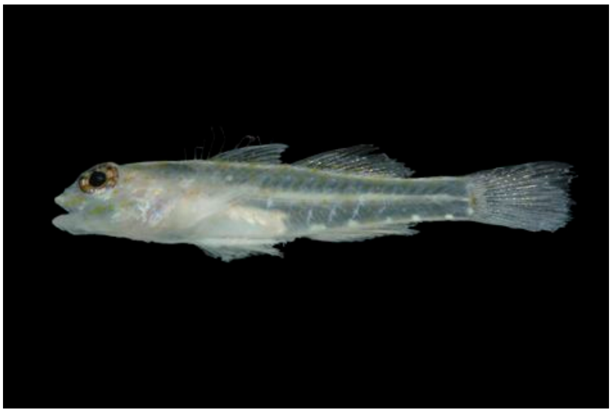

Figure S. 83. *Coryphopterus thrix*, CIUFES 2139, 22.29mm TL, from Davis seamount, photo by R. M. Macieira.

Elacatinus pridisi Guimarães, Gasparini & Rocha 2004 §‡

RS; TE; ET; L (30), O (84); Seamounts-Islands: VIT (CIUFES 2147), ALSAL (VID), ECL (VID), JAE (CIUFES 2190, 2191), DAV (VIS), DOG (CIUFES 2144), TRI (CIUFES 378, 1267, 1567, 2234, 2240, 2950, 131424; ZUEC-PIS 5412 - paratype) (Gasparini and Floeter [18], as *Elacatinus* sp.), MAR (CIUFES 2317) [13]; Figure S. 84.

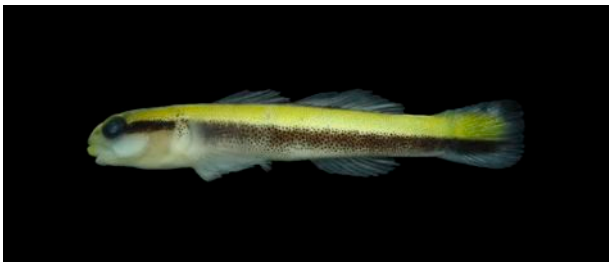

Figure S. 84. *Elacatinus pridisi*, CIUFES 2240, 21.43mm TL, from Trindade Island, photo by R. M. Macieira.

Gnatholepis thompsoni Jordan 1904 §‡

RH/SD; TA; NA; L (0-50), O (1-85); Seamounts-Islands: VIT (VIS), JAE (CIUFES 2150; ZUEC-PIS 8280), DOG (VID), COL (CIUFES 2159), TRI (CIUFES 1572, 2220, 2663, 2946) [18]; Figure S. 85.

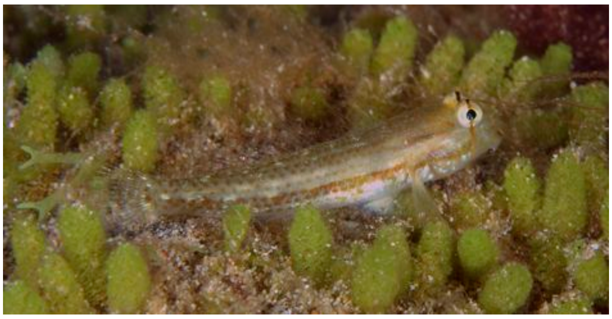

Figure S. 85. *Gnatholepis thompsoni*, underwater photo at Trindade Island, by R. M. Macieira.

Lythrypnus species 2 in Maxfield et al. 2012 §‡

RS/RH; VTC; NA; L (2-10); O (4-65); Seamounts-Islands: VIT (CIUFES2181), JAE (CIUFES 2169), DAV (CIUFES 2166), DOG (CIUFES 2176), TRI (CIUFES 2201) [18]; Figure S. 86.

An undescribed species.

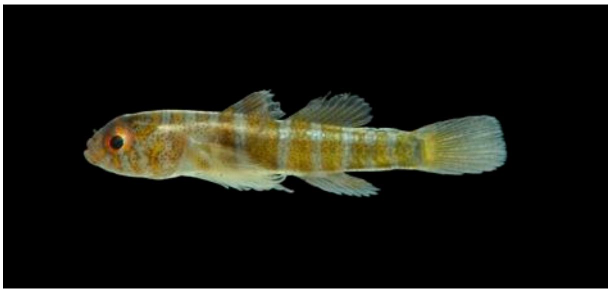

Figure S. 86. *Lythrypnus* species 2, CIUFES 2177, 9.54mm TL, from Dogaressa seamount, photo by R. M. Macieira.

Lythrypnus sp. §

RS/RH; VTC; NA; L (—); O (40-65); Seamounts-Islands: JAE (CIUFES 2172), DAV (CIUFES 2161), DOG (CIUFES 2178), TRI (CIUFES 2232) [20]; Figure S. 87.

An undescribed species.

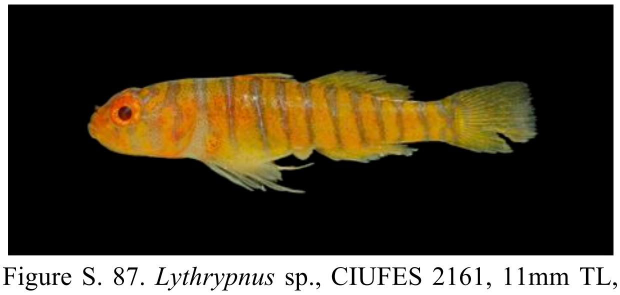

Figure S. 87. *Lythrypnus* sp., CIUFES 2161, 11mm TL, from Davis seamount, photo by R. M. Macieira.

Priolepis dawsoni Greenfield 1989 §

RS/RH; WA; NA; L (0-130); O (4-65); Seamounts-Islands: JAE (CIUFES 2193; ZUEC-PIS 8286), DOG (CIUFES 2175), TRI (CIUFES 1557, 1573); Figure S. 88.

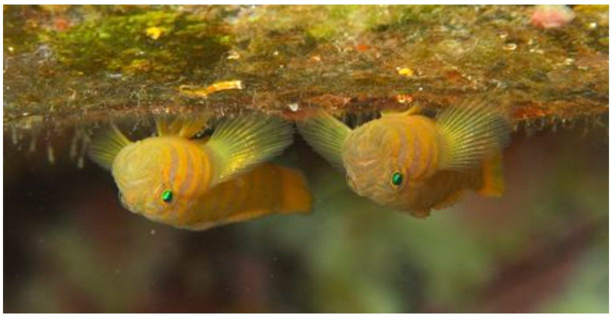

Figure S. 88. *Priolepis dawsoni*, CIUFES 1557 and 1573, both 36mm TL, underwater photo at Trindade Island, by R. M. Macieira.

Risor ruber (Rosén 1911) §

RH; WA; NA; L (30-180); O (65); Seamounts: DOG (CIUFES 2121; ZUEC-PIS 8270); Figure S. 89.

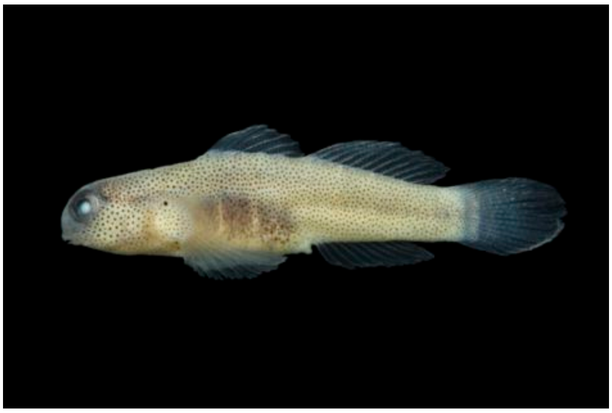

Figure S. 89. *Risor ruber*, CIUFES 2121, 11.60mm TL, from Dogaressa seamount, photo by R. M. Macieira.

Acanthuridae

Acanthurus bahianus Castelnau 1855 §‡

RS/RH; WA; NA; L (0-40), O (0-71); Seamounts-Islands: VIT (PHO), ECL (VIS), JAS (VIS), JAE (PHO), DAV (PHO), TRI (ZUEC-PIS 2826) [11], MAR [13]; Figure S. 90.

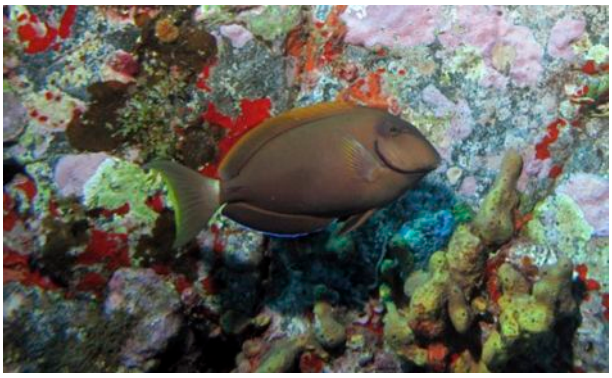

Figure S. 90. *Acanthurus bahianus*, underwater photo at Trindade Island, by H. T. Pinheiro.

Acanthurus chirurgus (Bloch 1787) §

RS/RH; TA; NA; L (2-70); O (20-62); Seamounts: VIT (PHO), JAS (VIS), JAE (VID), DAV (VID).

Acanthurus coeruleus Bloch & Schneider 1801 §‡

RS/RH; WA/CA; NA; L (2-70); O (0-71); Seamounts-Islands: VIT (VID), ECL (VIS), JAS (VIS), JAE (VID), DAV (PHO), DOG (VIS), TRI (FIS), TRI (ZUEC-PIS 2823) [18], MAR [13]; Figure S. 91.

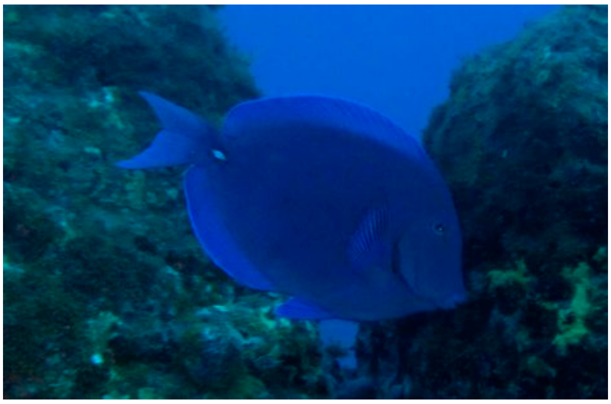

Figure S. 91. *Acanthurus coeruleus*, underwater photo at Trindade Island, by H. T. Pinheiro.

Sphyraenidae

Sphyraena barracuda (Walbaum 1792) §

RS/RH/WC; CG; NA; L (1-100); O (3-85); Seamounts-Islands: VIT (PHO), BESN (ZEE), JAE (VID), DAV (PHO), DOG (VIS), COL (VIS), TRI (CIUFES 1491) [12], MAR [13]; Figure S. 92.

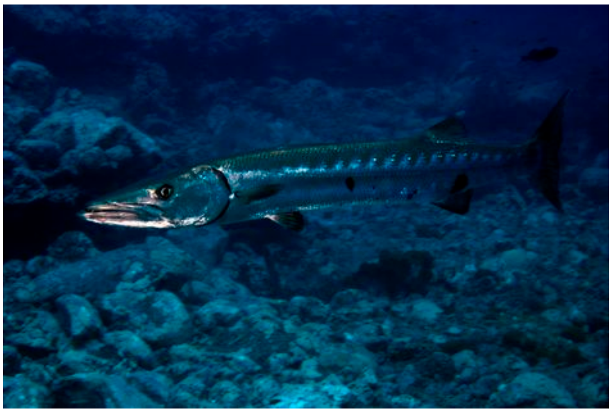

Figure S. 92. *Sphyraena barracuda*, underwater photo at Trindade island, by R. Francini-Filho.

Gempylidae

Gempylus serpens Cuvier 1829 §

WC; CG; NA; L (1-600); O (—); Seamounts: BESN (ZEE).

Lepidocybium flavobrunneum (Smith 1843) §‡

WC; CG; NA; L (200-1100), O (45-94); Seamounts: VIT [14], ALSAL [14], BESN [14], MONT (FIS).

Scombridae

Acanthocybium solandri (Cuvier 1832) §‡

WC; CG; NA; L (0-12), O (1-52); Seamounts-Islands: VIT (VIS), ALSAL (VIS), BESN (ZEE), ECL (VIS), MONT (FIS), JAE (VIS), DAV (VIS), TRI (VIS).

Katsuwonus pelamis (Linnaeus 1758) §

WC; CT; NA; L (0-260); O (0-10); Islands: MAR (PHO).

Scomberomorus cavalla (Cuvier 1829)

WC; WA/CA; NA; L (5-140); O (5-15); Islands: TRI [18].

Thunnus alalunga (Bonnaterre 1788) §

WC; CG; DD/CT; L (0-600); O (—); Seamounts-Islands: ALSAL (ZEE), TRI [12].

Thunnus albacares (Bonnaterre 1788) §

WC; CG; LC; L (1-250); O (—); Seamounts: VIT (FIS), ALSAL (ZEE).

Thunnus atlanticus (Lesson 1831) §

WC; WA; LC; L (50); O (10); Seamounts: ALSAL [14], BESN [14], MON [14], COL (PHO).

Thunnus obesus (Lowe 1839)

WC; CG; VU; L (0-250); O (18); Islands: TRI [10].

Xiphiidae

Xiphias gladius Linnaeus 1758

WC; CG; DD/CT; L (0-800); O (50); Seamounts-Islands: ALSAL [14], VIT [14], BESN [14], MONT [14], TRI [9].

Istiophoridae

Makaira nigricans Lacepède 1802 §

WC; TA; VU; L (0-200); O (56); Seamounts-Islands: VIT (FIS), BESN (ZEE), TRI (FIS).

Ariommatidae

Ariomma bondi Fowler, 1930 §‡

WC; TA; NA; L (50-500); O (49); Seamounts: DAV (ZEE).

Bothidae

Bothus lunatus (Linnaeus 1758) ‡

RH/SD; TA; NA; L (0-100), O (120); Islands: TRI (ZUEC-PIS 2695) [18], MAR [19]; Figure S. 93.

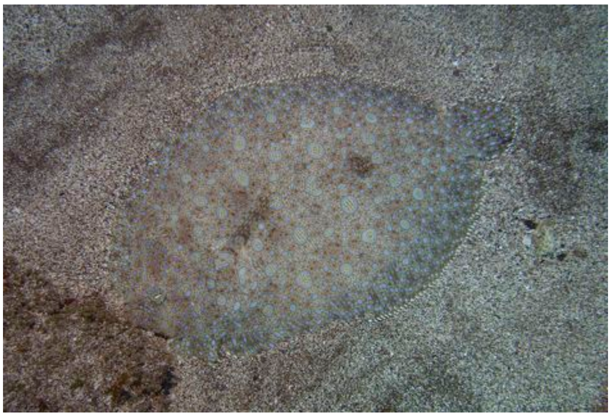

Figure S. 93. *Bothus lunatus*, underwater photo at Trindade Island, photo by J.-C. Joyeux.

Bothus maculiferus (Poey 1860) §

RH/SD; WA; NA; L (1-45); O (5-20); Islands: TRI (PHO); Figure S. 94.

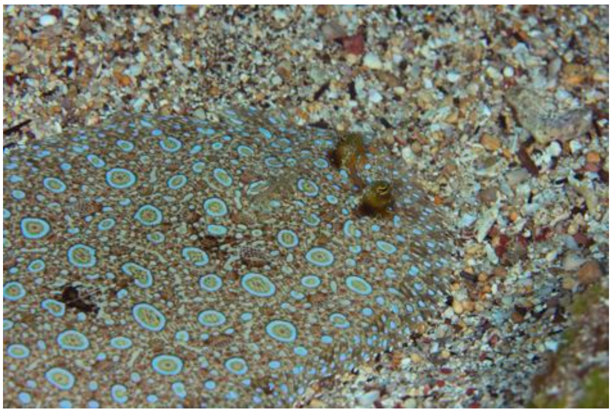

Figure S. 94. *Bothus maculiferus*, underwater photo at Trindade Island, by R. M. Macieira.

Cynoglossidae

Symphurus plagusia (Bloch & Schneider 1801)

SD; WA; NA; L (1-263); O (100); Seamounts: VIT [19].

Tetraodontiformes

Balistidae

Balistes capriscus Gmelin 1789 §

RH/WC; TA/M; NA; L (0-110); O (19-58); Seamounts-Islands: BESN (ZEE), JAS (ZEE), TRI [11].

Balistes vetula Linnaeus 1758 §‡

RS/RH/WC/SD; TA; VU; L (2-60), O (3-111); Seamounts-Islands: ALSAL (VIS), BESN (ZEE), ECL (PHO), JAS (PHO), MONT (ZEE), JAE (VIS), DAV (VID), DOG (VID), COL (ZEE), TRI (VID), TRI (ZUEC-PIS 2833) [8], MAR [13]; Figure S. 95.

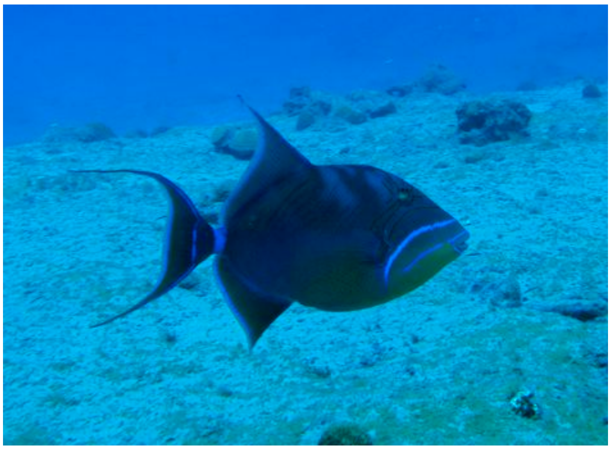

Figure S. 95. *Balistes vetula*, underwater photo at Trindade Island, by H. T. Pinheiro.

Canthidermis maculata (Bloch 1786)

WC; CG; NA; L (1-110); O (—); Islands: TRI [21].

Canthidermis sufflamen (Mitchill 1815) §‡

WC; TA; NA; L (5-75); O (5-300); Seamounts-Islands: VIT (PHO), ALSAL (VID), BESN (ZEE), ECL (VIS), JAE (VID), DAV (VID), DOG (ZEE), COL (VIS), TRI [18], MAR [13].

Melichthys niger (Bloch 1786) §

RS/RH/WC/SD; CT; NA; L (0-100); O (0-85); Seamounts-Islands: VIT (VID), JAS (VIS), JAE (PHO), DAV (PHO), DOG (VID), COL (VID), TRI (ZUEC-PIS 2702, 2825) [21], MAR [13]; Figure S. 96.

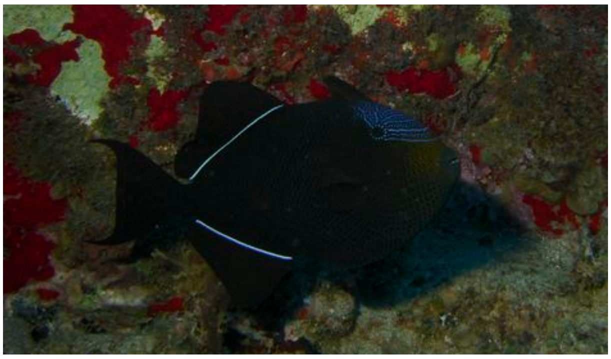

Figure S. 96. *Melichthys niger*, underwater photo at Trindade Island, by T. Simon.

Xanthichthys ringens (Linnaeus 1758) §

RH; WA; NA; L (25-130); O (62-66); Seamounts: ALSAL (PHO), JAS (PHO), DOG (CIUFES 2122, 2123) [19]; Figure S. 97.

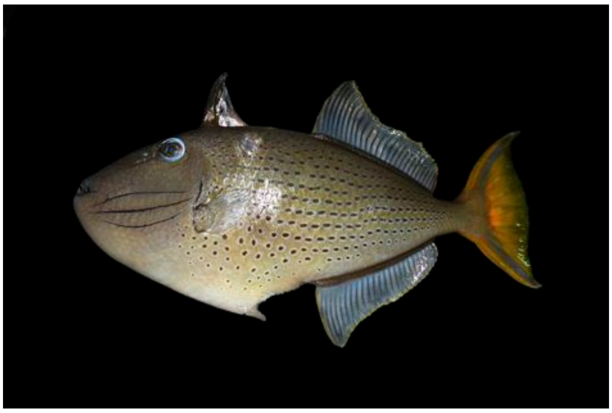

Figure S. 97. *Xanthichthys ringens*, CIUFES 2123, 197.17mm TL, from Dogaressa seamount, photo by R. M. Macieira.

Monacanthidae

Aluterus monoceros (Linnaeus 1758) §

WC; CT; NA; L (1-512), O (1-72); Seamounts: BESN (ZEE), VIT (ZEE).

Aluterus schoepfii (Walbaum 1792) §

RH; TA; NA; L (1-900); O (71); Seamounts: ECL (VIS).

Aluterus scriptus (Osbeck 1765) §

RS/RH; CT; NA; L (3-120); O (3-62); Seamounts-Islands: JAS (VIS), TRI (ZUEC-PIS 2828) [12], MAR [20]; Figure S. 98.

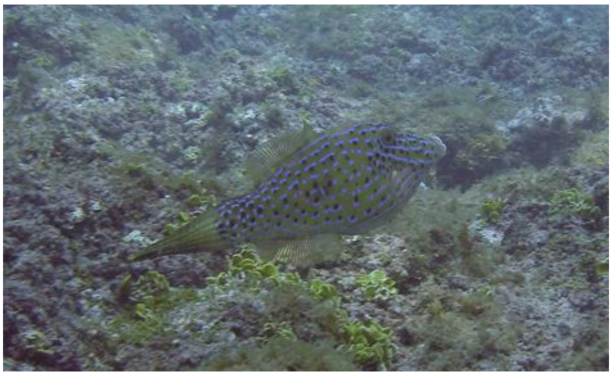

Figure S. 98. *Aluterus scriptus*, underwater photo at Trindade Island, by J.-C. Joyeux.

Cantherhines macrocerus (Hollard 1854) §

RS/RH; TA; NA; L (2-96), O (3-65); Seamounts-Islands: VIT (PHO), JAS (VIS), JAE (VID), DAV (VID), DOG (VIS), TRI (ZUEC-PIS 2824, 2692) [18], MAR [13]; Figure S. 99.

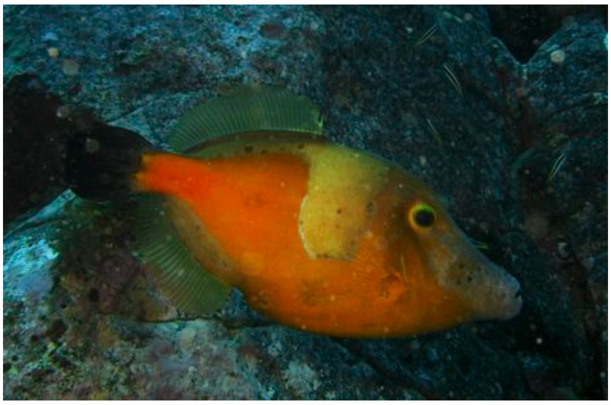

Figure S. 99. *Cantherhines macrocerus*, underwater photo at Trindade Island, by T. Simon.

Cantherhines pullus (Ranzani 1842) §‡

RS/RH; TA; NA; L (3-50), O (3-57); Seamounts-Islands: VIT [19], DAV (PHO), TRI [18], MAR [13]; Figure S. 100.

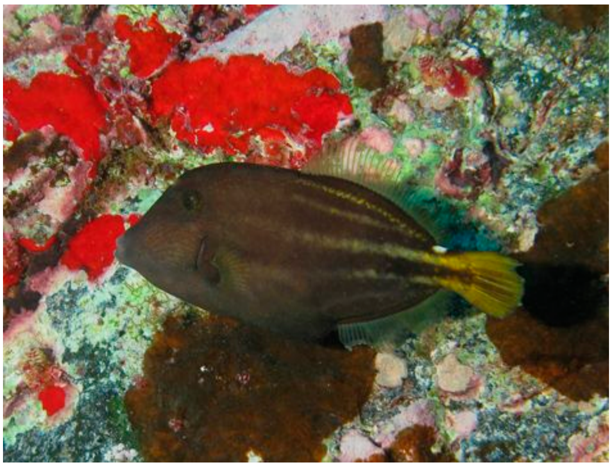

Figure S. 100. *Cantherhines pullus*, underwater photo at Trindade Island, by H. T. Pinheiro.

Stephanolepis hispidus (Linnaeus 1766)

RH; TA; NA; L (0-110); O (33-100); Seamounts: VIT [19].

Ostraciidae

Acanthostracion polygonius Poey 1876 §

RS; WA; NA; L (3-80); O (3-55); Seamounts-Islands: VIT (PHO), JAE (VID), TRI [13], MAR [20]; Figure S. 101.

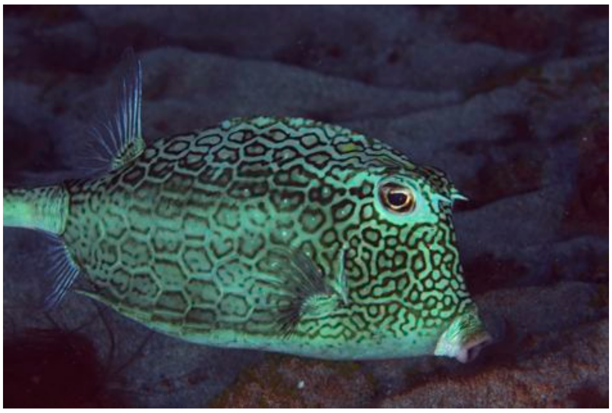

Figure S. 101. *Acanthostracion polygonius*, underwater photo at Trindade Island, by R. M. Macieira.

Acanthostracion quadricornis (Linnaeus 1758)

RS; TA; NA; L (4-80); O (7-18); Islands: TRI [18].

Tetraodontidae

Canthigaster figueiredoi Moura & Castro 2002 §‡

RS/RH; Br; NA; L (1-54), O (12-66); Seamounts-Islands; VIT (VIS), ALSAL (VIS), JAS (VIS), JAE (VIS), DAV (VIS), DOG (ZUEC-PIS 8274), TRI [10].

Sphoeroides spengleri (Bloch 1785)

RS; WA; NA; L (2-70); O (4-22); Islands: TRI (CIUFES 1545) [10], MAR [13]; Figure S. 102.

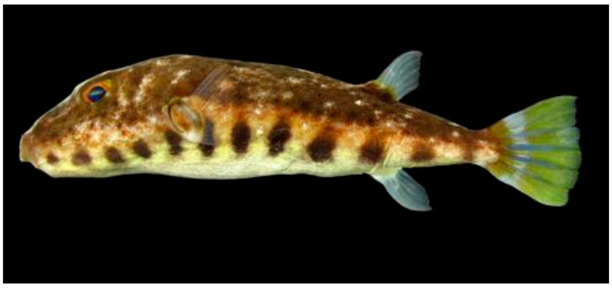

Figure S. 102. *Sphoeroides spengleri*, CIUFES 3116, 134.1mm TL, from Trindade Island, photo by H. T. Pinheiro.

Diodontidae

Chilomycterus reticulatus (Linnaeus 1758) §‡

RS; CG; NA; L (20-100); O (10-50); Seamounts-Islands: DAV (VIS), TRI [10]; Figure S. 103.

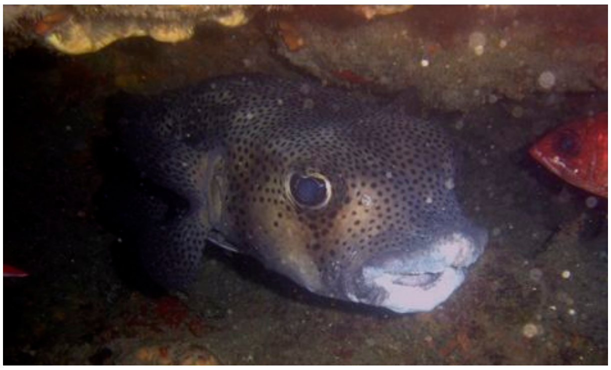

Figure S. 103. *Chilomycterus reticulatus*, underwater photo at Trindade Island, by H. T. Pinheiro.

Chilomycterus spinosus (Linnaeus 1758)

RS; SW; NA; L (190); O (10-20); Islands: TRI [10]; Figure S. 104.

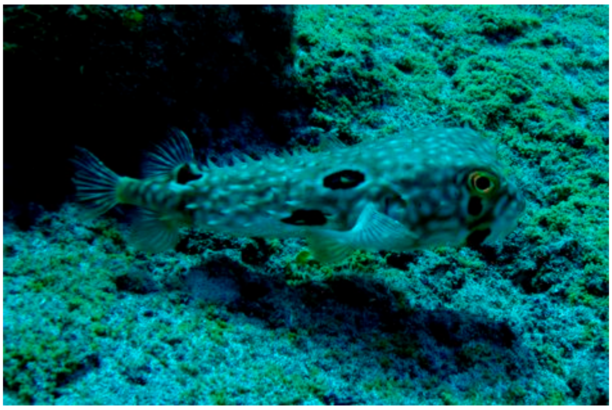

Figure S. 104. *Chilomycterus spinosus*, underwater photo at Trindade Island, by H. T. Pinheiro.

Diodon holocanthus Linnaeus 1758 §

RS; CT; NA; L (2-200); O (2-72); Seamounts-Islands: VIT [19], BESN (ZEE), VIT (ZEE), TRI (ZUEC-PIS 2819) [18], MAR [20]; Figure S. 105.

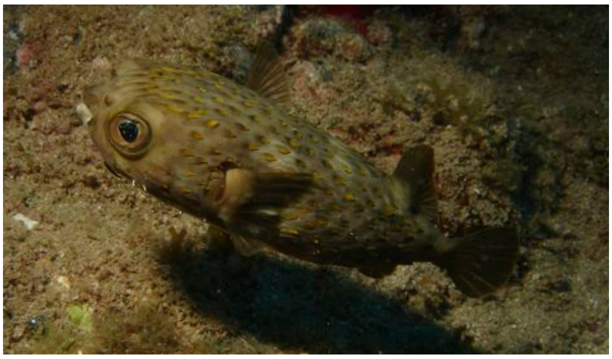

Figure S. 105. *Diodon holocanthus*, underwater photo at Trindade Island, by J.-C. Joyeux.

Diodon hystrix Linnaeus 1758

RS; CT; NA; L (2-135); O (3-20); Islands: TRI [12]; Figure S. 106.

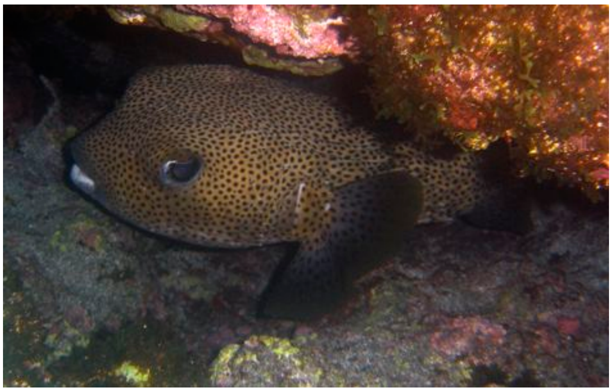

Figure S. 106. *Diodon hystrix*, underwater photo at Trindade Island, by H. T. Pinheiro.

Molidae

Masturus lanceolatus (Liénard 1840) §

WC; CT; NA; L (0-670); O (—); Seamounts: MON (FIS); Figure S. 107.

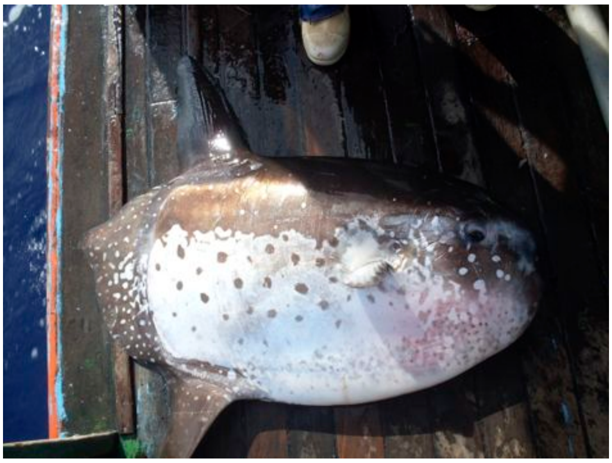

Figure S. 107. *Masturus lanceolatus*, from Montague seamount, photo by E. F. Mazzei.

Mola mola (Linnaeus 1758) §

WC; CG; NA; L (30-480); O (41-56); Seamounts: BESN (ZEE).

References

1. Nelson J (2006) Fishes of the world. Hoboken: John Wiley & Sons, Inc.
2. Craig MT, Hastings PA (2007) A molecular phylogeny of the groupers of the subfamily Epinephelinae (Serranidae) with a revised classification of the Epinephelini. Ichthyol Res 54: 1–17.
3. Craig MT, Mitcheson YJSD, Heemstra PC (2011) Groupers of the world: A field and market guide. Grahamstown: NISC.
4. Westneat MW, Alfaro ME (2005) Phylogenetic relationships and evolutionary history of the reef fish family Labridae. Mol Phylogenet Evol 36: 370–390. doi:10.1016/j.ympev.2005.02.001.
5. Eschmeyer WN (2013) Catalog of Fishes: Genera, Species, references. http://research.calacademy.org/research/ichthyology/catalog/fishcatmain.asp.
6. Briggs JC, Bowen BW (2012) A realignment of marine biogeographic provinces with particular reference to fish distributions. J Biogeogr 39: 12–30.
7. Motoki A, Motoki KF, Melo DP De (2012) Caracterização da morfologia submarina da cadeia Vitória-Trindade e áreas adjacentes-ES, com base na batimetria predita do topo versão 14.1. Rev Bras Geomorfol 13: 151–170.
8. Nichols JT, Murphy RC (1913) Fishes from South Trinidad Islet. Bull Am Museum Nat Hist 7: 261–266.
9. Pinheiro HT, Martins AS, Gasparini JL (2010) Impact of commercial fishing on Trindade Island and Martin Vaz Archipelago, Brazil: characteristics, conservation status of the species involved and prospects for preservation. Brazilian Arch Biol Technol 53: 1417–1423.
10. Pinheiro HT, Camilato V, Gasparini JL, Joyeux J-C (2009) New records of fishes for Trindade-Martin Vaz oceanic insular complex, Brazil. Zootaxa 2298: 45–54.
11. Miranda Ribeiro A (1919) A fauna vertebrada da Ilha da Trindade. Arch do Mus Nac 22: 171–194.
12. Carvalho J (1950) Resultados científicos do cruzeiro do “Baependí” e do “Vega” à I. da Trindade. Peixes. Bol do Inst Paul Oceanogr 1: 97–133.
13. Pereira-Filho G, Amado-Filho GM, Guimarães S, Moura RL, Sumida PY, et al. (2011) Reef fish and benthic assemblages of the Trindade and Martin Vaz island group, southwestern Atlantic. Brazilian J Oceanogr 59: 201–212.
14. Olavo G, Costa PAS, Martins AS (2005) Prospecção de grandes peixes pelágicos na região central da ZEE brasileira entre o Rio Real-BA e o Cabo de São Tomé-RJ. In: Costa PAS, Martins AS, Olavo G, editors. Pesca e potenciais de exploração de recursos vivos na região central da Zona Econômica Exclusiva brasileira. Rio de Janeiro: Museu Nacional, Vol. 202. pp. 167–202.
15. Martins A, Olavo G, Costa PAS (2005) Recursos demersais capturados com espinhel de fundo no talude superior da região entre Salvador (BA) e o Cabo de São Tomé. In: Costa P, Martins A, Olavo G, editors. Pesca e potenciais de exploração de recursos vivos na

região central da Zona Econômica Exclusiva brasileira. Rio de Janeiro: Museu Nacional, Vol. 128. pp. 109–128.

16. Gadig O, Sampaio CLS (2002) Ocorrência de *Mobula japanica* no Atlântico Ocidental e *Mobula tarapacana* em águas brasileiras, com comentários sobre a diversidade de raias-manta (Chondrichthyes: Mobulidae) no Brasil. Arq Ciência do Mar 35: 33–37.

17. Vaske Jr T, Lessa R, Nóbrega M, Montealegre-Quijano S, Marcante Santana F, et al. (2005) A checklist of fishes from Saint Peter and Saint Paul Archipelago, Brazil. J Appl Ichthyol 21: 75–79.

18. Gasparini JL, Floeter SR (2001) The shore fishes of Trindade Island, western South Atlantic. J Nat Hist 35: 1639–1656.

19. Andreata JV, Séret B (1995) Relação dos peixes coletados nos limites da plataforma continental e nas montanhas submarinas Vitória, Trindade e Martin Vaz, durante a campanha oceanográfica MD-55 Brasil. Rev Bras Zool 12: 579–594.

20. Simon T, Macieira RM, Joyeux J-C (2013) The shore fishes of the Trindade-Martin Vaz insular complex: an update. J Fish Biol 82: 2113–2127.

21. Murray G (1902) From Madeira to the Cape. Geogr J 19: 423–435.

22. Floeter SR, Rocha LA, Robertson DR, Joyeux JC, Smith-Vaniz WF, et al. (2008) Atlantic reef fish biogeography and evolution. J Biogeogr 35: 22–47. doi:10.1111/j.1365-2699.2007.01790.x.

23. Coelho N, Pinheiro HT, Guimarães R, Albuquerque CQ De, Martins AS (2012) Spatial distribution and diet of *Cephalopholis fulva* (Ephinephelidae) at Trindade Island, Brazil. 10: 383–388.

24. Batista H, Veras D, Oliveira P, Oliveira D, Tolotti M, et al. (2012) New records of reef fishes (Teleostei : Perciformes) in the Rocas Atoll Biological Reserve, off northeastern Brazil. Check List 8: 584–588.

25. Pinheiro HT, Ferreira CEL, Joyeux J-C, Santos RG, Horta PA (2011) Reef fish structure and distribution in a south-western Atlantic Ocean tropical island. J Fish Biol 79: 1984–2006.

26. Knudsen S, Clements KD (2013) Revision of the fish family Kyphosidae (Teleostei: Perciformes). Zootaxa 3751: 1–101.

27. Sakai K, Nakabo T (2014) Taxonomic review of *Kyphosus* (Pisces: Kyphosidae) in the Atlantic and eastern Pacific Oceans. Ichthyol Res 61: 265-292.

28. Joyeux J-C, Floeter SR, Ferreira CEL, Gasparini JL (2001) Biogeography of tropical reef fishes: the south Atlantic puzzle. J Biogeogr 28: 831–841.

29. Rocha L, Pinheiro HT, Gasparini JL (2010) Description of *Halichoeres rubrovirens*, a new species of wrasse (Labridae: Perciformes) from the Trindade and Martin Vaz Island group, southeastern Brazil, with a preliminary mtDNA molecular phylogeny of New World *Halichoeres*. Zootaxa 2422: 22–30.
